# At-admission prediction of mortality and pulmonary embolism in an international cohort of hospitalised patients with COVID-19 using statistical and machine learning methods

**DOI:** 10.1038/s41598-024-63212-7

**Published:** 2024-07-16

**Authors:** Munib Mesinovic, Xin Ci Wong, Giri Shan Rajahram, Barbara Wanjiru Citarella, Kalaiarasu M. Peariasamy, Frank van Someren Greve, Piero Olliaro, Laura Merson, Lei Clifton, Christiana Kartsonaki, ISARIC Characterisation Group, Sheryl Ann Abdukahil, Nurul Najmee Abdulkadir, Ryuzo Abe, Laurent Abel, Amal Abrous, Lara Absil, Andrew Acker, Shingo Adachi, Elisabeth Adam, Enrico Adriano, Diana Adrião, Saleh Al Ageel, Shakeel Ahmed, Marina Aiello, Kate Ainscough, Eka Airlangga, Tharwat Aisa, Ali Ait Hssain, Younes Ait Tamlihat, Takako Akimoto, Ernita Akmal, Eman Al Qasim, Razi Alalqam, Angela Alberti, Tala Al-dabbous, Senthilkumar Alegesan, Cynthia Alegre, Marta Alessi, Beatrice Alex, Kévin Alexandre, Abdulrahman Al-Fares, Huda Alfoudri, Adam Ali, Imran Ali, Kazali Enagnon Alidjnou, Jeffrey Aliudin, Qabas Alkhafajee, Clotilde Allavena, Nathalie Allou, João Alves, João Melo Alves, Rita Alves, Joana Alves Cabrita, Maria Amaral, Nur Amira, Heidi Ammerlaan, Phoebe Ampaw, Roberto Andini, Claire Andréjak, Andrea Angheben, François Angoulvant, Séverine Ansart, Sivanesen Anthonidass, Massimo Antonelli, Carlos Alexandre Antunes de Brito, Kazi Rubayet Anwar, Ardiyan Apriyana, Yaseen Arabi, Irene Aragao, Francisco Arancibia, Carolline Araujo, Antonio Arcadipane, Patrick Archambault, Lukas Arenz, Jean-Benoît Arlet, Christel Arnold-Day, Lovkesh Arora, Rakesh Arora, Elise Artaud-Macari, Diptesh Aryal, Motohiro Asaki, Angel Asensio, Elizabeth A. Ashley, Muhammad Ashraf, Jean Baptiste Assie, Amirul Asyraf, Anika Atique, AM Udara Lakshan Attanyake, Johann Auchabie, Hugues Aumaitre, Adrien Auvet, Laurène Azemar, Cecile Azoulay, Benjamin Bach, Delphine Bachelet, Claudine Badr, Nadia Baig, J. Kenneth Baillie, J Kevin Baird, Erica Bak, Agamemnon Bakakos, Nazreen Abu Bakar, Andriy Bal, Mohanaprasanth Balakrishnan, Valeria Balan, Firouzé Bani-Sadr, Renata Barbalho, Nicholas Yuri Barbosa, Wendy S. Barclay, Saef Umar Barnett, Michaela Barnikel, Helena Barrasa, Audrey Barrelet, Cleide Barrigoto, Marie Bartoli, Joaquín Baruch, Romain Basmaci, Muhammad Fadhli Hassin Basri, Denise Battaglini, Jules Bauer, Diego Fernando Bautista Rincon, Denisse Bazan Dow, Abigail Beane, Alexandra Bedossa, Ker Hong Bee, Husna Begum, Sylvie Behilill, Albertus Beishuizen, Aleksandr Beljantsev, David Bellemare, Anna Beltrame, Beatriz Amorim Beltrão, Marine Beluze, Nicolas Benech, Lionel Eric Benjiman, Dehbia Benkerrou, Suzanne Bennett, Luís Bento, Jan-Erik Berdal, Delphine Bergeaud, Hazel Bergin, Giulia Bertoli, Lorenzo Bertolino, Simon Bessis, Adam Betz, Sybille Bevilcaqua, Karine Bezulier, Amar Bhatt, Krishna Bhavsar, Claudia Bianco, Farah Nadiah Bidin, Moirangthem Bikram Singh, Felwa Bin Humaid, Mohd Nazlin Bin Kamarudin, Zeno Bisoffi, François Bissuel, Patrick Biston, Laurent Bitker, Jonathan Bitton, Pablo Blanco-Schweizer, Catherine Blier, Frank Bloos, Mathieu Blot, Lucille Blumberg, Filomena Boccia, Laetitia Bodenes, Debby Bogaert, Anne-Hélène Boivin, Isabela Bolaños, Pierre-Adrien Bolze, François Bompart, Patrizia Bonelli, Aurelius Bonfasius, Diogo Borges, Raphaël Borie, Hans Martin Bosse, Elisabeth Botelho-Nevers, Lila Bouadma, Olivier Bouchaud, Sabelline Bouchez, Dounia Bouhmani, Damien Bouhour, Kévin Bouiller, Laurence Bouillet, Camile Bouisse, Thipsavanh Bounphiengsy, Latsaniphone Bountthasavong, Anne-Sophie Boureau, John Bourke, Maude Bouscambert, Aurore Bousquet, Jason Bouziotis, Bianca Boxma, Marielle Boyer-Besseyre, Maria Boylan, Fernando Augusto Bozza, Axelle Braconnier, Cynthia Braga, Timo Brandenburger, Filipa Brás Monteiro, Luca Brazzi, Dorothy Breen, Patrick Breen, David Brewster, Kathy Brickell, Alex Browne, Shaunagh Browne, Nicolas Brozzi, Marjolein Brusse-Keizer, Nina Buchtele, Polina Bugaeva, Marielle Buisson, Danilo Buonsenso, Erlina Burhan, Aidan Burrell, Ingrid G. Bustos, Denis Butnaru, André Cabie, Susana Cabral, Eder Caceres, Cyril Cadoz, Rui Caetano Garcês, Mia Callahan, Kate Calligy, Jose Andres Calvache, Caterina Caminiti, João Camões, Valentine Campana, Paul Campbell, Josie Campisi, Cecilia Canepa, Mireia Cantero, Pauline Caraux-Paz, Sheila Cárcel, Chiara Simona Cardellino, Filipa Cardoso, Filipe Cardoso, Nelson Cardoso, Sofia Cardoso, Simone Carelli, Francesca Carlacci, Nicolas Carlier, Thierry Carmoi, Gayle Carney, Inês Carqueja, Marie-Christine Carret, François Martin Carrier, Ida Carroll, Gail Carson, Leonor Carvalho, Maire-Laure Casanova, Mariana Cascão, Siobhan Casey, José Casimiro, Bailey Cassandra, Silvia Castañeda, Nidyanara Castanheira, Guylaine Castor-Alexandre, Ivo Castro, Ana Catarino, François-Xavier Catherine, Paolo Cattaneo, Roberta Cavalin, Giulio Giovanni Cavalli, Alexandros Cavayas, Adrian Ceccato, Shelby Cerkovnik, Minerva Cervantes-Gonzalez, Anissa Chair, Catherine Chakveatze, Bounthavy Chaleunphon, Adrienne Chan, Meera Chand, Christelle Chantalat Auger, Jean-Marc Chapplain, Charlotte Charpentier, Julie Chas, Allegra Chatterjee, Jonathan Samuel Chávez Iñiguez, Anjellica Chen, Léo Chenard, Matthew Pellan Cheng, Antoine Cheret, Alfredo Antonio Chetta, Thibault Chiarabini, Julian Chica, Suresh Kumar Chidambaram, Leong Chin Tho, Catherine Chirouze, Davide Chiumello, Hwa Jin Cho, Sung-Min Cho, Bernard Cholley, Danoy Chommanam, Marie-Charlotte Chopin, Ting Soo Chow, Yock Ping Chow, Nathaniel Christy, Hiu Jian Chua, Jonathan Chua, Jose Pedro Cidade, José Miguel Cisneros Herreros, Barbara Wanjiru Citarella, Anna Ciullo, Emma Clarke, Jennifer Clarke, Rolando Claure-Del Granado, Sara Clohisey, Cassidy Codan, Caitriona Cody, Alexandra Coelho, Jennifer Coles, Megan Coles, Gwenhaël Colin, Michael Collins, Sebastiano Maria Colombo, Pamela Combs, Marie Connor, Anne Conrad, Sofía Contreras, Elaine Conway, Graham S. Cooke, Mary Copland, Hugues Cordel, Amanda Corley, Sabine Cornelis, Alexander Daniel Cornet, Arianne Joy Corpuz, Andrea Cortegiani, Grégory Corvaisier, Emma Costigan, Camille Couffignal, Sandrine Couffin-Cadiergues, Roxane Courtois, Stéphanie Cousse, Rachel Cregan, Charles Crepy D’Orleans, Cosimo Cristella, Sabine Croonen, Gloria Crowl, Jonathan Crump, Claudina Cruz, Marc Csete, Ailbhe Cullen, Matthew Cummings, Ger Curley, Elodie Curlier, Colleen Curran, Paula Custodio, Ana da Silva Filipe, Charlene Da Silveira, Al-Awwab Dabaliz, Andrew Dagens, Darren Dahly, Heidi Dalton, Jo Dalton, Seamus Daly, Juliana Damas, Nick Daneman, Corinne Daniel, Emmanuelle A Dankwa, Jorge Dantas, Frédérick D’Aragon, Menno de Jong, Gillian de Loughry, Diego de Mendoza, Etienne De Montmollin, Rafael Freitas de Oliveira França, Ana Isabel de Pinho Oliveira, Rosanna De Rosa, Cristina De Rose, Thushan de Silva, Peter de Vries, Jillian Deacon, David Dean, Alexa Debard, Marie-Pierre Debray, Nathalie DeCastro, William Dechert, Lauren Deconninck, Romain Decours, Eve Defous, Isabelle Delacroix, Eric Delaveuve, Karen Delavigne, Nathalie M. Delfos, Ionna Deligiannis, Andrea Dell’Amore, Christelle Delmas, Pierre Delobel, Corine Delsing, Elisa Demonchy, Emmanuelle Denis, Dominique Deplanque, Pieter Depuydt, Mehul Desai, Diane Descamps, Mathilde Desvallées, Santi Dewayanti, Pathik Dhanger, Alpha Diallo, Sylvain Diamantis, André Dias, Andrea Dias, Fernanda Dias Da Silva, Juan Jose Diaz, Priscila Diaz, Rodrigo Diaz, Kévin Didier, Jean-Luc Diehl, Wim Dieperink, Jérôme Dimet, Vincent Dinot, Fara Diop, Alphonsine Diouf, Yael Dishon, Félix Djossou, Annemarie B. Docherty, Helen Doherty, Arjen M Dondorp, Andy Dong, Christl A. Donnelly, Maria Donnelly, Chloe Donohue, Sean Donohue, Yoann Donohue, Peter Doran, Céline Dorival, Eric D’Ortenzio, Phouvieng Douangdala, James Joshua Douglas, Renee Douma, Nathalie Dournon, Triona Downer, Joanne Downey, Mark Downing, Tom Drake, Aoife Driscoll, Murray Dryden, Claudio Duarte Fonseca, Vincent Dubee, François Dubos, Audrey Dubot-Pérès, Alexandre Ducancelle, Toni Duculan, Susanne Dudman, Abhijit Duggal, Paul Dunand, Jake Dunning, Mathilde Duplaix, Emanuele Durante-Mangoni, Lucian Durham, Bertrand Dussol, Juliette Duthoit, Xavier Duval, Anne Margarita Dyrhol-Riise, Sim Choon Ean, Marco Echeverria-Villalobos, Giorgio Economopoulos, Siobhan Egan, Carla Eira, Mohamed El Sanharawi, Subbarao Elapavaluru, Brigitte Elharrar, Jacobien Ellerbroek, Philippine Eloy, Tarek Elshazly, Iqbal Elyazar, Isabelle Enderle, Tomoyuki Endo, Chan Chee Eng, Ilka Engelmann, Vincent Enouf, Olivier Epaulard, Martina Escher, Mariano Esperatti, Hélène Esperou, Catarina Espírito Santo, Marina Esposito-Farese, João Estevão, Manuel Etienne, Nadia Ettalhaoui, Anna Greti Everding, Mirjam Evers, Isabelle Fabre, Marc Fabre, Amna Faheem, Arabella Fahy, Cameron J. Fairfield, Zul Fakar, Pedro Faria, Hanan Fateena, Arie Zainul Fatoni, Karine Faure, Raphaël Favory, Mohamed Fayed, Niamh Feely, Laura Feeney, Jorge Fernandes, Marília Andreia Fernandes, Susana Fernandes, François-Xavier Ferrand, Eglantine Ferrand Devouge, Joana Ferrão, Carlo Ferrari, Mário Ferraz, Benigno Ferreira, Bernardo Ferreira, Isabel Ferreira, Sílvia Ferreira, Ricard Ferrer-Roca, Nicolas Ferriere, Céline Ficko, Claudia Figueiredo-Mello, William Finlayson, Juan Fiorda, Thomas Flament, Clara Flateau, Tom Fletcher, Aline-Marie Florence, Letizia Lucia Florio, Brigid Flynn, Deirdre Flynn, Federica Fogliazza, Claire Foley, Jean Foley, Victor Fomin, Tatiana Fonseca, Patricia Fontela, Simon Forsyth, Denise Foster, Giuseppe Foti, Erwan Fourn, Robert A. Fowler, Marianne Fraher, Diego Franch-Llasat, Christophe Fraser, John F Fraser, Marcela Vieira Freire, Ana Freitas Ribeiro, Caren Friedrich, Ricardo Fritz, Stéphanie Fry, Nora Fuentes, Masahiro Fukuda, Argin G, Valérie Gaborieau, Rostane Gaci, Massimo Gagliardi, Jean-Charles Gagnard, Amandine Gagneux-Brunon, Sérgio Gaião, Linda Gail Skeie, Phil Gallagher, Elena Gallego Curto, Carrol Gamble, Yasmin Gani, Arthur Garan, Rebekha Garcia, Julia Garcia-Diaz, Esteban Garcia-Gallo, Navya Garimella, Federica Garofalo, Denis Garot, Valérie Garrait, Nathalie Gault, Aisling Gavin, Anatoliy Gavrylov, Alexandre Gaymard, Johannes Gebauer, Eva Geraud, Louis Gerbaud Morlaes, Nuno Germano, praveen kumar ghisulal, Jade Ghosn, Marco Giani, Carlo Giaquinto, Jess Gibson, Tristan Gigante, Morgane Gilg, Elaine Gilroy, Guillermo Giordano, Michelle Girvan, Valérie Gissot, Daniel Glikman, Petr Glybochko, Eric Gnall, Geraldine Goco, François Goehringer, Siri Goepel, Jean-Christophe Goffard, Jin Yi Goh, Jonathan Golob, Rui Gomes, Kyle Gomez, Joan Gómez-Junyent, Marie Gominet, Alicia Gonzalez, Patricia Gordon, Isabelle Gorenne, Laure Goubert, Cécile Goujard, Tiphaine Goulenok, Margarite Grable, Jeronimo Graf, Edward Wilson Grandin, Pascal Granier, Giacomo Grasselli, Christopher A. Green, Courtney Greene, William Greenhalf, Segolène Greffe, Domenico Luca Grieco, Matthew Griffee, Fiona Griffiths, Ioana Grigoras, Albert Groenendijk, Anja Grosse Lordemann, Heidi Gruner, Yusing Gu, Jérémie Guedj, Martin Guego, Dewi Guellec, Anne-Marie Guerguerian, Daniela Guerreiro, Romain Guery, Anne Guillaumot, Laurent Guilleminault, Maisa Guimarães de Castro, Thomas Guimard, Marieke Haalboom, Daniel Haber, Hannah Habraken, Ali Hachemi, Nadir Hadri, Sheeba Hakak, Adam Hall, Matthew Hall, Sophie Halpin, Ansley Hamer, Rebecca Hamidfar, Naomi Hammond, Terese Hammond, Lim Yuen Han, Rashan Haniffa, Kok Wei Hao, Hayley Hardwick, Ewen M. Harrison, Janet Harrison, Samuel Bernard Ekow Harrison, Alan Hartman, Junaid Hashmi, Ailbhe Hayes, Leanne Hays, Jan Heerman, Lars Heggelund, Ross Hendry, Martina Hennessy, Aquiles Henriquez-Trujillo, Maxime Hentzien, Diana Hernandez, Jaime Hernandez-Montfort, Andrew Hershey, Liv Hesstvedt, Astarini Hidayah, Dawn Higgins, Eibhlin Higgins, Rupert Higgins, Rita Hinchion, Samuel Hinton, Hiroaki Hiraiwa, Haider Hirkani, Hikombo Hitoto, Antonia Ho, Yi Bin Ho, Alexandre Hoctin, Isabelle Hoffmann, Wei Han Hoh, Oscar Hoiting, Rebecca Holt, Jan Cato Holter, Peter Horby, Juan Pablo Horcajada, Koji Hoshino, Kota Hoshino, Ikram Houas, Catherine L. Hough, Stuart Houltham, Jimmy Ming-Yang Hsu, Jean-Sébastien Hulot, Stella Huo, Abby Hurd, Samreen Ijaz, M. Arfan Ikram, Carlos Cañada Illana, Hajnal-Gabriela Illes, Patrick Imbert, Hugo Inácio, Carmen Infante Dominguez, Yun Sii Ing, Elias Iosifidis, Mariachiara Ippolito, Vera Irawany, Sarah Isgett, Tiago Isidoro, Nadiah Ismail, Margaux Isnard, Junji Itai, Asami Ito, Daniel Ivulich, Danielle Jaafar, Salma Jaafoura, Julien Jabot, Clare Jackson, Nina Jamieson, Victoria Janes, Pierre Jaquet, Waasila Jassat, Coline Jaud-Fischer, Stéphane Jaureguiberry, Jeffrey Javidfar, Denise Jaworsky, Florence Jego, Anilawati Mat Jelani, Synne Jenum, Ruth Jimbo-Sotomayor, Ong Yiaw Joe, Ruth N. Jorge García, Cédric Joseph, Mark Joseph, Swosti Joshi, Mercé Jourdain, Philippe Jouvet, Anna Jung, Hanna Jung, Dafsah Juzar, Ouifiya Kafif, Florentia Kaguelidou, Neerusha Kaisbain, Thavamany Kaleesvran, Sabina Kali, Alina Kalicinska, Smaragdi Kalomoiri, Muhammad Aisar Ayadi Kamaluddin, Zul Amali Che Kamaruddin, Nadiah Kamarudin, Darshana Hewa Kandamby, Chris Kandel, Kong Yeow Kang, Pratap Karpayah, Todd Karsies, Christiana Kartsonaki, Daisuke Kasugai, Anant Kataria, Kevin Katz, Aasmine Kaur, Christy Kay, Hannah Keane, Seán Keating, Pulak Kedia, Andrea Kelly, Aoife Kelly, Claire Kelly, Niamh Kelly, Sadie Kelly, Yvelynne Kelly, Maeve Kelsey, Ryan Kennedy, Kalynn Kennon, Sommay Keomany, Maeve Kernan, Younes Kerroumi, Sharma Keshav, Evelyne Kestelyn, Imrana Khalid, Antoine Khalil, Coralie Khan, Irfan Khan, Krish Kherajani, Michelle E Kho, Denisa Khoo, Ryan Khoo, Saye Khoo, Khor How Kiat, Yuri Kida, Peter Kiiza, Beathe Kiland Granerud, Anders Benjamin Kildal, Jae Burm Kim, Antoine Kimmoun, Detlef Kindgen-Milles, Alexander King, Nobuya Kitamura, Paul Klenerman, Rob Klont, Gry Kloumann Bekken, Stephen R Knight, Robin Kobbe, Chamira Kodippily, Malte Kohns Vasconcelos, Mamoru Komatsu, ISARIC Collaborator Korten, Caroline Kosgei, Arsène Kpangon, Karolina Krawczyk, Sudhir Krishnan, Vinothini Krishnan, Oksana Kruglova, Deepali Kumar, Ganesh Kumar, Pavan Kumar Vecham, Dinesh Kuriakose, Ethan Kurtzman, Neurinda Permata Kusumastuti, Demetrios Kutsogiannis, Galyna Kutsyna, Konstantinos Kyriakoulis, Raph L. Hamers, Marie Lachatre, Marie Lacoste, John G. Laffey, Nadhem Lafhej, Marie Lagrange, Fabrice Laine, Olivier Lairez, Marc Lambert, François Lamontagne, Marie Langelot-Richard, Vincent Langlois, Eka Yudha Lantang, Marina Lanza, Cédric Laouénan, Samira Laribi, Delphine Lariviere, Stéphane Lasry, Sakshi Lath, Odile Launay, Didier Laureillard, Yoan Lavie-Badie, Andrew Law, Cassie Lawrence, Teresa Lawrence, Minh Le, Clément Le Bihan, Cyril Le Bris, Georges Le Falher, Lucie Le Fevre, Quentin Le Hingrat, Marion Le Maréchal, Soizic Le Mestre, Gwenaël Le Moal, Vincent Le Moing, Hervé Le Nagard, Paul Le Turnier, Ema Leal, Marta Leal Santos, Biing Horng Lee, Heng Gee Lee, James Lee, Jennifer Lee, Su Hwan Lee, Todd C. Lee, Yi Lin Lee, Gary Leeming, Bénédicte Lefebvre, Laurent Lefebvre, Benjamin Lefèvre, Sylvie LeGac, Jean-Daniel Lelievre, François Lellouche, Adrien Lemaignen, Véronique Lemee, Anthony Lemeur, Gretchen Lemmink, Ha Sha Lene, Jenny Lennon, Rafael León, Marc Leone, Michela Leone, François-Xavier Lescure, Olivier Lesens, Mathieu Lesouhaitier, Amy Lester-Grant, Andrew Letizia, Sophie Letrou, Bruno Levy, Yves Levy, Claire Levy-Marchal, Katarzyna Lewandowska, Erwan L’Her, Gianluigi Li Bassi, Janet Liang, Geoffrey Liegeon, Kah Chuan Lim, Wei Shen Lim, Chantre Lima, Bruno Lina, Lim Lina, Andreas Lind, Maja Katherine Lingad, Guillaume Lingas, Sylvie Lion-Daolio, Samantha Lissauer, Keibun Liu, Marine Livrozet, Patricia Lizotte, Antonio Loforte, Navy Lolong, Leong Chee Loon, Diogo Lopes, Dalia Lopez-Colon, Jose W. Lopez-Revilla, Anthony L. Loschner, Paul Loubet, Bouchra Loufti, Guillame Louis, Silvia Lourenco, Lara Lovelace-Macon, Lee Lee Low, Marije Lowik, Jia Shyi Loy, Jean Christophe Lucet, Carlos M. Luna, Olguta Lungu, Liem Luong, Nestor Luque, Dominique Luton, Nilar Lwin, Ruth Lyons, Olavi Maasikas, Oryane Mabiala, Moïse Machado, Sara Machado, Gabriel Macheda, Hashmi Madiha, Giuseppe Maglietta, Rafael Mahieu, Sophie Mahy, Ana Raquel Maia, Lars S. Maier, Mylène Maillet, Thomas Maitre, Maria Majori, Maximilian Malfertheiner, Nadia Malik, Paddy Mallon, Fernando Maltez, Denis Malvy, Patrizia Mammi, Victoria Manda, Jose M. Mandei, Laurent Mandelbrot, Frank Manetta, Julie Mankikian, Edmund Manning, Aldric Manuel, Ceila Maria Sant‘Ana Malaque, Daniel Marino, Flávio Marino, Samuel Markowicz, Ana Marques, Catherine Marquis, Brian Marsh, Megan Marshal, John Marshall, Celina Turchi Martelli, Dori-Ann Martin, Emily Martin, Guillaume Martin-Blondel, Ignacio Martin-Loeches, Martin Martinot, Alejandro Martín-Quiros, F. Eduardo Martinez, Ana Martins, João Martins, Nuno Martins, Caroline Martins Rego, Gennaro Martucci, Olga Martynenko, Eva Miranda Marwali, Marsilla Marzukie, Juan Fernado Masa Jimenez, David Maslove, Sabina Mason, Basri Mat Nor, Moshe Matan, Daniel Mathieu, Mathieu Mattei, Romans Matulevics, Laurence Maulin, Michael Maxwell, Javier Maynar, Mayfong Mayxay, Thierry Mazzoni, Lisa Mc Sweeney, Peter McCanny, Colin McArthur, Aine McCarthy, Anne McCarthy, Colin McCloskey, Rachael McConnochie, Sherry McDermott, Sarah E. McDonald, Aine McElroy, Samuel McElwee, Victoria McEneany, Natalie McEvoy, Allison McGeer, Chris McKay, Johnny McKeown, Kenneth A. McLean, Paul McNally, Bairbre McNicholas, Elaine McPartlan, Edel Meaney, Cécile Mear-Passard, Maggie Mechlin, Omar Mehkri, Ferruccio Mele, Luis Melo, Joao Joao Mendes, Ogechukwu Menkiti, Kusum Menon, France Mentré, Alexander J. Mentzer, Emmanuelle Mercier, Noémie Mercier, Antoine Merckx, Mayka Mergeay-Fabre, Blake Mergler, Laura Merson, Tiziana Meschi, António Mesquita, Roberta Meta, Osama Metwally, Agnès Meybeck, Dan Meyer, Alison M. Meynert, Vanina Meysonnier, Amina Meziane, Mehdi Mezidi, Céline Michelanglei, Isabelle Michelet, Efstathia Mihelis, Vladislav Mihnovit, Jennene Miller, Hugo Miranda-Maldonado, Nor Arisah Misnan, Nik Nur Eliza Mohamed, Tahira Jamal Mohamed, Asma Moin, Elena Molinos, Brenda Molloy, Mary Mone, Agostinho Monteiro, Claudia Montes, Giorgia Montrucchio, Sarah Moore, Shona C. Moore, Lina Morales Cely, Lucia Moro, Ben Morton, Catherine Motherway, Ana Motos, Hugo Mouquet, Clara Mouton Perrot, Julien Moyet, Caroline Mudara, Ng Yong Muh, Dzawani Muhamad, Jimmy Mullaert, Fredrik Müller, Karl Erik Müller, Daniel Munblit, Aisling Murphy, Lorna Murphy, Marlène Murris, Srinivas Murthy, Himed Musaab, Carlotta Mutti, Himasha Muvindi, Gugapriyaa Muyandy, Dimitra Melia Myrodia, Farah Nadia Mohd-Hanafiah, Dave Nagpal, Alex Nagrebetsky, Mangala Narasimhan, Nageswaran Narayanan, Alasdair Nazerali-Maitland, Nadège Neant, Holger Neb, Coca Necsoi, Nikita Nekliudov, Erni Nelwan, Raul Neto, Emily Neumann, Pauline Yeung Ng, Anthony Nghi, Duc Nguyen, Orna Ni Choileain, Niamh Ni Leathlobhair, Alistair D Nichol, Prompak Nitayavardhana, Stephanie Nonas, Nurul Amani Mohd Noordin, Marion Noret, Nurul Faten Izzati Norharizam, Lisa Norman, Anita North, Alessandra Notari, Mahdad Noursadeghi, Karolina Nowicka, Adam Nowinski, Saad Nseir, Jose I Nunez, Nurnaningsih Nurnaningsih, Dwi Utomo Nusantara, Elsa Nyamankolly, Fionnuala O Brien, Annmarie O Callaghan, Annmarie O’Callaghan, Giovanna Occhipinti, Derbrenn OConnor, Max O’Donnell, Tawnya Ogston, Takayuki Ogura, Tak-Hyuk Oh, Sophie O’Halloran, Katie O’Hearn, Shinichiro Ohshimo, Agnieszka Oldakowska, João Oliveira, Larissa Oliveira, Piero L. Olliaro, Conar O’Neil, David S. Y. Ong, Jee Yan Ong, Wilna Oosthuyzen, Anne Opavsky, Peter Openshaw, Claudia Milena Orozco-Chamorro, Jamel Ortoleva, Javier Osatnik, Linda O’Shea, Miriam O’Sullivan, Siti Zubaidah Othman, Nadia Ouamara, Rachida Ouissa, Eric Oziol, Maïder Pagadoy, Justine Pages, Amanda Palacios, Massimo Palmarini, Giovanna Panarello, Prasan Kumar Panda, Lai Hui Pang, Mauro Panigada, Nathalie Pansu, Aurélie Papadopoulos, Paolo Parducci, Edwin Fernando Paredes Oña, Rachael Parke, Melissa Parker, Jérémie Pasquier, Bruno Pastene, Fabian Patauner, Drashti Patel, Mohan Dass Pathmanathan, Luís Patrão, Patricia Patricio, Juliette Patrier, Laura Patrizi, Lisa Patterson, Christelle Paul, Mical Paul, Jorge Paulos, William A. Paxton, Jean-François Payen, Sandra L Peake, Kalaiarasu Peariasamy, Giles J. Peek, Florent Peelman, Nathan Peiffer-Smadja, Vincent Peigne, Mare Pejkovska, Paolo Pelosi, Ithan D. Peltan, Rui Pereira, Daniel Perez, Luis Periel, Thomas Perpoint, Antonio Pesenti, Vincent Pestre, Lenka Petrou, Michele Petrovic, Ventzislava Petrov-Sanchez, Frank Olav Pettersen, Gilles Peytavin, Scott Pharand, Ooyanong Phonemixay, Soulichanya Phoutthavong, Michael Piagnerelli, Walter Picard, Olivier Picone, Maria de Piero, Carola Pierobon, Djura Piersma, Carlos Pimentel, Raquel Pinto, Valentine Piquard, Catarina Pires, Isabelle Pironneau, Lionel Piroth, Roberta Pisi, Ayodhia Pitaloka, Chiara Piubelli, Riinu Pius, Laurent Plantier, Hon Shen Png, Julien Poissy, Ryadh Pokeerbux, Maria Pokorska-Spiewak, Sergio Poli, Georgios Pollakis, Diane Ponscarme, Jolanta Popielska, Diego Bastos Porto, Andra-Maris Post, Douwe F. Postma, Pedro Povoa, Diana Póvoas, Jeff Powis, Sofia Prapa, Viladeth Praphasiri, Sébastien Preau, Christian Prebensen, Jean-Charles Preiser, Anton Prinssen, Mark G. Pritchard, Gamage Dona Dilanthi Priyadarshani, Lucia Proença, Sravya Pudota, Oriane Puéchal, Bambang Pujo Semedi, Matteo Puntoni, Gregory Purcell, Luisa Quesada, Vilmaris Quinones-Cardona, Else Quist-Paulsen, Mohammed Quraishi, Maia Rabaa, Christian Rabaud, Aldo Rafael, Marie Rafiq, Gabrielle Ragazzo, Mutia Rahardjani, Ahmad Kashfi Haji Ab Rahman, Rozanah Abd Rahman, Fernando Rainieri, Giri Shan Rajahram, Nagarajan Ramakrishnan, José Ramalho, Ahmad Afiq Ramli, Blandine Rammaert, Grazielle Viana Ramos, Anais Rampello, Rajavardhan Rangappa, Ritika Ranjan, Elena Ranza, Christophe Rapp, Aasiyah Rashan, Thalha Rashan, Menaldi Rasmin, Indrek Rätsep, Cornelius Rau, Francesco Rausa, Tharmini Ravi, Andre Real, Stanislas Rebaudet, Sarah Redl, Brenda Reeve, Liadain Reid, Dag Henrik Reikvam, Renato Reis, Jordi Rello, Jonathan Remppis, Martine Remy, Hongru Ren, Hanna Renk, Anne-Sophie Resseguier, Matthieu Revest, Oleksa Rewa, Luis Felipe Reyes, Tiago Reyes, Maria Ines Ribeiro, Antonia Ricchiuto, David Richardson, Denise Richardson, Laurent Richier, Siti Nurul Atikah Ahmad Ridzuan, Jordi Riera, Ana L Rios, Asgar Rishu, Patrick Rispal, Karine Risso, Maria Angelica Rivera Nuñez, Nicholas Rizer, Chiara Robba, André Roberto, David L. Robertson, Olivier Robineau, Ferran Roche-Campo, Paola Rodari, Simão Rodeia, Julia Rodriguez Abreu, Bernhard Roessler, Claire Roger, Pierre-Marie Roger, Emmanuel Roilides, Amanda Rojek, Juliette Romaru, Roberto Roncon-Albuquerque, Mélanie Roriz, Manuel Rosa-Calatrava, Michael Rose, Dorothea Rosenberger, Andrea Rossanese, Matteo Rossetti, Sandra Rossi, Bénédicte Rossignol, Patrick Rossignol, Stella Rousset, Carine Roy, Benoît Roze, Desy Rusmawatiningtyas, Clark D. Russell, Maeve Ryan, Maria Ryan, Steffi Ryckaert, Aleksander Rygh Holten, Isabela Saba, Luca Sacchelli, Musharaf Sadat, Valla Sahraei, Nadia Saidani, Maximilien Saint-Gilles, Pranya Sakiyalak, Leonardo Salazar, Gabriele Sales, Stéphane Sallaberry, Charlotte Salmon Gandonniere, Hélène Salvator, Olivier Sanchez, Angel Sanchez-Miralles, Vanessa Sancho-Shimizu, Gyan Sandhu, Zulfiqar Sandhu, Pierre-François Sandrine, Oana Sandulescu, Marlene Santos, Shirley Sarfo-Mensah, Bruno Sarmento Banheiro, Iam Claire E. Sarmiento, Benjamine Sarton, Ankana Satya, Sree Satyapriya, Rumaisah Satyawati, Egle Saviciute, Parthena Savvidou, Yen Tsen Saw, Justin Schaffer, Tjard Schermer, Arnaud Scherpereel, Marion Schneider, Stephan Schroll, Michael Schwameis, Gary Schwartz, Brendan Scicluna, Janet T. Scott, James Scott-Brown, Nicholas Sedillot, Tamara Seitz, Mageswari Selvarajoo, Caroline Semaille, Malcolm G. Semple, Rasidah Bt Senian, Eric Senneville, Claudia Sepulveda, Filipa Sequeira, Tânia Sequeira, Ary Serpa Neto, Ellen Shadowitz, Syamin Asyraf Shahidan, Mohammad Shamsah, Anuraj Shankar, Shaikh Sharjeel, Pratima Sharma, Catherine A. Shaw, Victoria Shaw, Rajesh Mohan Shetty, Rohan Shetty, Haixia Shi, Nisreen Shiban, Mohiuddin Shiekh, Takuya Shiga, Nobuaki Shime, Hiroaki Shimizu, Keiki Shimizu, Sally Shrapnel, Hoi Ping Shum, Nassima Si Mohammed, Ng Yong Siang, Jeanne Sibiude, Bountoy Sibounheuang, Atif Siddiqui, Louise Sigfrid, Piret Sillaots, Catarina Silva, Maria Joao Silva, Rogério Silva, Benedict Sim Lim Heng, Wai Ching Sin, Dario Sinatti, Budha Charan Singh, Punam Singh, Pompini Agustina Sitompul, Karisha Sivam, Vegard Skogen, Sue Smith, Benjamin Smood, Coilin Smyth, Michelle Smyth, Morgane Snacken, Dominic So, Tze Vee Soh, Joshua Solomon, Tom Solomon, Emily Somers, Agnès Sommet, Myung Jin Song, Rima Song, Tae Song, Jack Song Chia, Michael Sonntagbauer, Azlan Mat Soom, Albert Sotto, Edouard Soum, Ana Chora Sousa, Marta Sousa, Maria Sousa Uva, Vicente Souza-Dantas, Alexandra Sperry, Elisabetta Spinuzza, B. P. Sanka Ruwan Sri Darshana, Shiranee Sriskandan, Sarah Stabler, Thomas Staudinger, Stephanie-Susanne Stecher, Trude Steinsvik, Ymkje Stienstra, Birgitte Stiksrud, Eva Stolz, Amy Stone, Adrian Streinu-Cercel, Anca Streinu-Cercel, Ami Stuart, David Stuart, Richa Su, Decy Subekti, Gabriel Suen, Jacky Y. Suen, Asfia Sultana, Charlotte Summers, Dubravka Supic, Deepashankari Suppiah, Magdalena Surovcová, Atie Suwarti, Andrey Svistunov, Sarah Syahrin, Konstantinos Syrigos, Jaques Sztajnbok, Konstanty Szuldrzynski, Shirin Tabrizi, Fabio S. Taccone, Lysa Tagherset, Shahdattul Mawarni Taib, Ewa Talarek, Sara Taleb, Jelmer Talsma, Renaud Tamisier, Maria Lawrensia Tampubolon, Kim Keat Tan, Le Van Tan, Yan Chyi Tan, Clarice Tanaka, Hiroyuki Tanaka, Taku Tanaka, Hayato Taniguchi, Coralie Tardivon, Pierre Tattevin, M Azhari Taufik, Hassan Tawfik, Richard S. Tedder, Tze Yuan Tee, João Teixeira, Sofia Tejada, Marie-Capucine Tellier, Sze Kye Teoh, Vanessa Teotonio, François Téoulé, Pleun Terpstra, Olivier Terrier, Nicolas Terzi, Hubert Tessier-Grenier, Adrian Tey, Alif Adlan Mohd Thabit, Zhang Duan Tham, Suvintheran Thangavelu, Vincent Thibault, Simon-Djamel Thiberville, Benoît Thill, Jananee Thirumanickam, Shaun Thompson, David Thomson, Emma C. Thomson, Surain Raaj Thanga Thurai, Duong Bich Thuy, Ryan S. Thwaites, Andrea Ticinesi, Paul Tierney, Vadim Tieroshyn, Peter S Timashev, Jean-François Timsit, Noémie Tissot, Jordan Zhien Yang Toh, Maria Toki, Kristian Tonby, Sia Loong Tonnii, Marta Torre, Antoni Torres, Margarida Torres, Rosario Maria Torres Santos-Olmo, Hernando Torres-Zevallos, Michael Towers, Tony Trapani, Huynh Trung Trieu, Théo Trioux, Cécile Tromeur, Ioannis Trontzas, Tiffany Trouillon, Jeanne Truong, Christelle Tual, Sarah Tubiana, Helen Tuite, Alexis F. Turgeon, Jean-Marie Turmel, Lance C. W. Turtle, Anders Tveita, Pawel Twardowski, Makoto Uchiyama, PG Ishara Udayanga, Andrew Udy, Roman Ullrich, Alberto Uribe, Asad Usman, Timothy M. Uyeki, Cristinava Vajdovics, Piero Valentini, Luís Val-Flores, Amélie Valran, Ilaria Valzano, Stijn Van de Velde, Marcel van den Berge, Machteld Van der Feltz, Job van der Palen, Paul van der Valk, Nicky Van Der Vekens, Peter Van der Voort, Sylvie Van Der Werf, Laura van Gulik, Jarne Van Hattem, Carolien van Netten, Frank van Someren Greve, Gitte Van Twillert, Ilonka van Veen, Hugo Van Willigen, Noémie Vanel, Henk Vanoverschelde, Pooja Varghese, Michael Varrone, Shoban Raj Vasudayan, Charline Vauchy, Shaminee Veeran, Aurélie Veislinger, Sebastian Vencken, Sara Ventura, Annelies Verbon, James Vickers, José Ernesto Vidal, César Vieira, Joy Ann Villanueva, Judit Villar, Pierre-Marc Villeneuve, Andrea Villoldo, Nguyen Van Vinh Chau, Gayatri Vishwanathan, Benoit Visseaux, Hannah Visser, Chiara Vitiello, Manivanh Vongsouvath, Harald Vonkeman, Fanny Vuotto, Noor Hidayu Wahab, Suhaila Abdul Wahab, Nadirah Abdul Wahid, Marina Wainstein, Steve Webb, Jia Wei, Katharina Weil, Tan Pei Wen, Sanne Wesselius, T. Eoin West, Murray Wham, Bryan Whelan, Paul Henri Wicky, Aurélie Wiedemann, Surya Otto Wijaya, Keith Wille, Sue Willems, Patricia J Williams, Virginie Williams, Evert-Jan Wils, Ng Wing Yiu, Calvin Wong, Teck Fung Wong, Xin Ci Wong, Yew Sing Wong, Natalie Wright, Gan Ee Xian, Lim Saio Xian, Kuan Pei Xuan, Ioannis Xynogalas, Sophie Yacoub, Siti Rohani Binti Mohd Yakop, Masaki Yamazaki, Elizabeth Yarad, Yazdan Yazdanpanah, Nicholas Yee Liang Hing, Cécile Yelnik, Chian Hui Yeoh, Stephanie Yerkovich, Touxiong Yiaye, Toshiki Yokoyama, Hodane Yonis, Obada Yousif, Saptadi Yuliarto, Akram Zaaqoq, Marion Zabbe, Gustavo E Zabert, Kai Zacharowski, Masliza Zahid, Maram Zahran, Nor Zaila Binti Zaidan, Maria Zambon, Miguel Zambrano, Alberto Zanella, Konrad Zawadka, Nurul Zaynah, Hiba Zayyad, Alexander Zoufaly, David Zucman

**Affiliations:** 1 Department of Engineering Science, University of Oxford, Oxford, UK; 2 Digital Health Research and Innovation Unit, Institute for Clinical Research, National Institutes of Health (NIH), Shah Alam, Malaysia; 3 Queen Elizabeth II Hospital, Ministry of Health, Kota Kinabalu, Malaysia; 4 Pandemic Sciences Institute, ISARIC, University of Oxford, Oxford, UK; 5 Department of Medical Microbiology, Amsterdam University Medical Center, Amsterdam, The Netherlands; 6 Nuffield Department of Population Health, University of Oxford, Oxford, UK; 7 King Abdulaziz Medical City, Riyadh, Saudi Arabia; 8 Tuanku Fauziah Hospital, Perlis, Malaysia; 9 Chiba University Hospital, Chiba, Japan; 10 INSERM, Paris, France; 11 CUB-Hopital Erasme, Anderlecht, Belgium; 12 Perelman School of Medicine, University of Pennsylvania, Philadelphia, USA; 13 Rinku General Medical Center, Osaka, Japan; 14 Uniklinik University Hospital, Frankfurt, Germany; 15 University Hospital of Parma, Parma, Italy; 16 Centro Hospitalar Vila Nova de Gaia/Espinho, Espinho, Portugal; 17 King Faisal Hospital Research Center, Riyadh, Saudi Arabia; 18 University Hospital, Kerry, Ireland; 19 St Vincents University Hospital, Dublin, Ireland; 20 Murni Teguh Memorial Hospital, Bunda Thamrin Hospital, North Sumatera, Indonesia; 21 Our lady of Lourdes Drogheda, Drogheda, Ireland; 22 Hamad General Hospital, Doha, Qatar; 23 Centre Hospitalier de Saintonge, Saintes, France; 24 Teine Keijinkai Hospital, Sapporo, Japan; 25 Persahabatan Hospital, Jakarta, Indonesia; 26 Beaumont Hospital, Dublin, Ireland; 27 Northwell Health, New York, USA; 28 Al-Adan Hospital, Hadiya, Kuwait; 29 Galway University Hospital, Galway, Ireland; 30 Hospital Vall d’Hebron, Barcelona, Spain; 31 University Hospital Policlinico Paolo Giaccone, Palermo, Italy; 32 School of Informatics, University of Edinburgh, Edinburgh, UK; 33 Centre Hospitalier Universitaire Rouen (Center Hospitalier Universitaire de Rouen), Rouen, France; 34 Al-Amiri & Jaber Al-Ahmed Hospitals, Kuwait City, Kuwait; 35 St Bernard’s Hospital, Gibraltar, Gibraltar; 36 Sligo University Hospital (Saolta), Sligo, Ireland; 37 Centre Hospitalier Universitaire de Lille, Lille, France; 38 Centre Hospitalier Universitaire de Nantes (Hôpital femme-enfant-adolescent), Nantes, France; 39 Centre Hospitalier Félix-Guyon, Saint-Denis, Réunion; 40 Centro Hospital e Universitário de Coimbra, Coimbra, Portugal; 41 Hospital de São José -U.U.M, Lisbon, Portugal; 42 Hospital Curry Cabral - Intensive Care Unit - UCIP7, Lisbon, Portugal; 43 Sungai Buloh Hospital, Selangor, Malaysia; 44 Catharina Ziekenhuis, Eindhoven, The Netherlands; 45 The Ottawa Hospital, Ottawa, Canada; 46 University of Campania, Carseta, Italy; 47 Centre Hospitalier Universitaire Amiens-Picardie, Amiens, France; 48 Department of Infectious - Tropical Diseases and Microbiology, IRCCS Sacro Cuore Hospital - Negrar, Negrar di Valpolicella, Italy; 49 Centre Hospitalier Universitaire de Brest, Brest, France; 50 Kuala Lumpur Hospital, WPKL, Kuala Lumpur, Malaysia; 51 Fondazione Policlinico Universitario Agostino Gemelli IRCCS, Rome, Italy; 52 Centro de Pesquisa Aggeu Magalhães, Fiocruz, Recife, Brazil; 53 NICVD Dhaka, Dhaka, Bangladesh; 54 National Cardiovascular Center Harapan Kita Jakarta Indonesia, Jakarta, Indonesia; 55 Intensive Care Department, Ministry of National Guard Health Affairs, Riyadh, Saudi Arabia; 56 Centro Hospitalar Universitário do Porto (CHUP), Porto, Portugal; 57 Instituto Nacional Del Tórax, Santiago, Chile; 58 Istituto Mediterraneo per i Trapianti e Terapie ad Alta Specializzazione, Palermo, Italy; 59 CISSS Chaudière-Appalaches, Quèbec, Canada; 60 Medical Department II, LMU Hospital Munich, Campus Großhadern, Munich, Germany; 61 Hôpital Européen Georges-Pompidou AP-HP, Paris, France; 62 Groote Schuur Hospital, Cape Town, South Africa; 63 University of Iowa, Iowa City, USA; 64 St. Boniface Hospital, Winnipeg , Manitoba Canada; 65 CCA Network, Asia, Chiang Mai, Thailand; 66 Fujieda Municipal General Hospital, Fujieda, Japan; 67 Hospital Puerta de Hierro Majadahonda, Madrid, Spain; 68 Lao-Oxford-Mahosot Hospital-Wellcome Trust Research Unit, Vientiane, Laos Thailand; 69 Centre Hospitalier intercommunal de Créteil, Créteil, France; 70 McGill University Health Centre, Montreal, Canada; 71 Centre Hospitalier de Cholet, Cholet, France; 72 Centre Hospitalier de Perpignan, Perpignan, France; 73 Hôpital Lariboisière AP-HP, Paris, France; 74 Hôpital Cochin AP-HP, Paris, France; 75 Centre Hospitalier Intercommunal Villeneuve-Saint-Georges, Villeneuve-Saint-Georges, France; 76 Grande Prairie Queen Elizabeth II, Grande Prairie, Canada; 77 Roslin Institute, University of Edinburgh, Edinburgh, UK; 78 Rush University Medical Center, Chicago, USA; 79 Sotiria General Hospital, Athens, Greece; 80 Unidade Local de Saúde de Alto Minho, Viana Do Castelo, Portugal; 81 Centre Hospitalier Universitaire de Reims, Reims, France; 82 Instituto de Infectologia Emílio Ribas, Sao Paulo, Brazil; 83 Caja Nacional De Salud, Trinidad, Bolivia; 84 Section of Molecular Virology, Imperial College London, London, UK; 85 Hospital Universitario de Alava, Araba, Spain; 86 Grand Hôpital de l’Est Francilien (Site de Marne-la-Vallée), Jossigny, France; 87 San Martino Hospital, Genoa, Italy; 88 Clinica Valle de Lilli, Valle del Cauca, Colombia; 89 University of Washington Medical Center - Northwest, Seattle, USA; 90 Critical Care Asia, Bangkok, Thailand; 91 Raja Permaisuri Bainun Hospital, Perak, Malaysia; 92 WHO-ISARIC Clinical Characterisation Protocol & SPRINT-SARI, London, UK; 93 Medisch Spectrum Twente, Zutphen, The Netherlands; 94 Tartu University Hospital, Tartu, Estonia; 95 Hôpital de l’Enfant-Jésus, Quebec, Quebec Canada; 96 Follow Up Study Working Group, London, UK; 97 Sao Camilo Cura D’ars, Fortaleza, Brazil; 98 Centre Hospitalier Universitaire de Lyon - HCL, Lyon, France; 99 Sarawak General Hospital, Sarawak, Malaysia; 100 University of Cincinnati, Cincinnati, USA; 101 Akershus University Hospital, Nordbyhagen, Norway; 102 Ospedale Sacro Cuore Don Calabria, Negrar Di Valpolicella, Italy; 103 Hôpital Raymond-Poincaré, Garches, France; 104 Oklahoma Heart Institute, Oklahoma, USA; 105 Centre Hospitalier Régional et Universitaire de Nancy - Hôpitaux de Brabois, Nancy, France; 106 Hôpital de la Timone, Marseille, France; 107 Ohio State University, Columbus, USA; 108 All India Institute of Medical Sciences, Rishikesh, India; 109 Department of Infectious, Tropical Diseases and Microbiology, IRCCS Sacro Cuore Don Calabria Hospital, Negrar, Italy; 110 Thonon-les-Bains , Thonon-les-Bains, France; 111 Civil Hospital Marie Curie, Charleroi, Belgium; 112 Hôpital Lyon Sud - HCL, Lyon, France; 113 The Centre hospitalier universitaire Sainte-Justine, Montreal, Canada; 114 Rio Hortega University Hospital, Valladolid, Spain; 115 Jena University Hospital, Jena, Germany; 116 Centre Hospitalier Universitaire Mitterrand Dijon-Bourgogne, Dijon, France; 117 National Institute for Communicable Diseases, Johannesburg, South Africa; 118 Centre for Inflammation Research, The Queen’s Medical Research Institute, University of Edinburgh, 47 Little France Crescent, Edinburgh, UK; 119 Centre Hospitalier de Dax - Côte d’Argent, Dax, France; 120 Universidad del Cauca, Cauca, Colombia; 121 Pratama Rada Bolo Hospital, Karitas Hospital and Waikabubak Hospital, Sumba, Indonesia; 122 Hôpital Bichat Claude-Bernard AP-HP, Paris, France; 123 University Hospital Dusseldorf, Dusseldorf, Germany; 124 Centre Hospitalier Universitaire de Saint-Étienne, Saint-Étienne, France; 125 Hôpital Avicenne, Bobigny, France; 126 Centre hospitalier de l’université de Montréal, Montreal, Canada; 127 Centre Hospitalier de Bourg-en-Bresse, Bourg-en-Bresse, France; 128 Centre Hospitalier Universitaire de Besançon, Besançon, France; 129 Centre Hospitalier Universitaire Grenoble-Alpes, Grenoble, France; 130 Luang Namtha Provincial Hospital, Luang Namtha, Laos; 131 Xieng Khouang Provincial Hospital, Phonsavan, Laos; 132 Centre Hospitalier Universitaire de Nantes (Hôtel-Dieu), Nantes, France; 133 Hôpital d’Instruction des Armées Bégin, Saint-Mandé, France; 134 Franciscus Gasthuis, Rotterdam, The Netherlands; 135 Ministry of Health, and D’Or Institute of Research and Education (IDOR), National Institute of Infectious Disease Evandro Chagas, Oswaldo Cruz Foundation (INI-FIOCRUZ), Rio de Janeiro, Brazil; 136 Centre Hospitalier de Mayotte, Mamoudzou, Mayotte; 137 Hospital Egas Moniz, Lisboa, Portugal; 138 Ospedale Molinette, Torino, Italy; 139 Cork University Hospital, Cork, Ireland; 140 Beacon Hospital, Dublin, Ireland; 141 Monash University, Melbourne, Australia; 142 Nelson Hospital, Nelson, New Zealand; 143 Cleveland Clinic, Weston, USA; 144 Medical University of Vienna, Vienna, Austria; 145 Sechenov University, Moscow, Russia; 146 Clinica Universidad de La Sabana, Cundinamarca, Chia Colombia; 147 Centre Hospitalier Universitaire de Martinique, Fort-de-France, Saint Martin, France; 148 Centre Hospitalier Régional Metz-Thionville, Metz, France; 149 Emory University Healthcare System, Atlanta, USA; 150 Johns Hopkins, Baltimore, USA; 151 Comissão de Ética, Unidade Local de Saúde de Matosinhos, Porto, Portugal; 152 Presbyterian Hospital Services, Alberquerque, USA; 153 Hospital del Mar, Barcelona, Spain; 154 Reina Sofia University Hospital, Cordoba, Spain; 155 Hospital Espírito Santo de Évora, Évora, Portugal; 156 Hôpital Américain de Paris, Neuilly-sur-Seine, France; 157 Vancouver Island Health, Vancouver, Canada; 158 Centre Hospitalier Métropole Savoie, Chambéry, France; 159 Department of Anesthesiology, Centre hospitalier de l’Université de Montréal, Montreal, USA; 160 University Hospital - Limerick, Limerick, Ireland; 161 Centro Hospitalar e Universitário de Coimbra - Hospital Pediátrico, Coimbra, Portugal; 162 Centre Hospitalier de Béziers, Béziers, France; 163 Hospital São Francisco Xavier, Lisbon, Portugal; 164 Policlinicodi Orsola Universitàdi Bologna, Bologna, Italy; 165 Hospital du Sacre Coeur, Montreal, Canada; 166 Hospital Universitari Sagrat Cor, Barcelona, Spain; 167 Avera McKennan Hospital, Sioux Falls, USA; 168 Centre Hospitalier de Melun, Melun, France; 169 Attapeu Provincial Hospital, Attapeu, Laos; 170 Sunnybrook Health Sciences Centre, Toronto, Canada; 171 Antimicrobial Resistance and Hospital Acquired Infection Department, Public Health England, London, UK; 172 Hôpital Kremlin-Bicêtre, Le Kremlin-Bicêtre, France; 173 Centre Hospitalier Universitaire Rennes (Hôpital Pontchaillou), Rennes, France; 174 Hôpital Tenon AP-HP, Paris, France; 175 University of Guadalajara Health Sciences Center, Guadalajara, Mexico; 176 Höpital Saint-Antoine AP-HP, Paris, France; 177 National Institutes of Health (NIH), Ministry of Health Malaysia, Setia Alam, Malaysia; 178 Ospedale San Paolo, Milan, Italy; 179 Chonnam National University Hospital, Dong-gu, South Korea; 180 Salavan Provincial Hospital, Salavan, Laos; 181 Pulau Pinang Hospital, Pulau Pinang, Malaysia; 182 Sunway Medical Centre, Selangor, Malaysia; 183 University Hospital Virgen del Rocío / Institute of Biomedicine of Seville, Seville, Spain; 184 University of Utah, Salt Lake City, USA; 185 Children’s Health Ireland, Dublin, Ireland; 186 Roslin Institute, University of Edinburgh, Easter Bush, Edinburgh, UK; 187 Foothills Medical Centre, Calgary, Canada; 188 Connolly Hospital Blanchardstown, Dublin, Ireland; 189 Carilion Clinic, Roanoke, USA; 190 Centre Hospitalier Départemental Vendée, La Roche-sur-Yon, France; 191 Allegheny General Hospital, Pittsburgh, USA; 192 Fondazione IRCCS Ca, Milan, Italy; 193 University of Chicago, Chicago, USA; 194 Liverpool Clinical Trials Centre, University of Liverpool, Liverpool, UK; 195 Department of Infectious Disease, Imperial College London, London, UK; 196 Brantford General Hospital, Brantford, Canada; 197 University of Queensland, Brisbane, Australia; 198 Centre Hospitalier Bretagne Atlantique, Vannes, France; 199 Hôpital Jacques Monod, Le Havre, France; 200 PREPARE and RECOVER EU Consortium, Brussels, Belgium; 201 Tergooi Hospital, Hilversum, The Netherlands; 202 Michael Garron Hospital, Toronto, Canada; 203 Hospital de Curry Cabral - Infectious Diseases, Lisbon, Portugal; 204 Mount Sinai Medical Center, Miami, FL USA; 205 Columbia University, New York, USA; 206 Centre Hospitalier Universitaire de Guadeloupe, Pointe-à-Pitre, Guadeloupe; 207 MRC-University of Glasgow Centre for Virus Research, 464 Bearsden Road, Glasgow, UK; 208 UH Cleveland Hospital, Cleveland, USA; 209 University Hospital - Waterford, Waterford, Ireland; 210 Saint-Martin , Saint-Martin, Guadeloupe; 211 Department of Statistics, University of Oxford, Oxford, UK; 212 Centre hospitalier Universitaire de Sherbrooke, Sherbrooke, Canada; 213 Centro Hospitalar de Tondela-Viseu, Viseu, Portugal; 214 Monaldi Hospital, Napoli, Italy; 215 Università Cattolica del Sacro Cuore, Rome, Italy; 216 Department of Infection, Immunity and Cardiovascular Disease, The Florey Institute for Host-Pathogen Interactions, University of Sheffield, Sheffield, UK; 217 St Christopher’s Hospital for Children, Philadelphia, USA; 218 Piedmont Atlanta Hospital, Atlanta, Georgia USA; 219 Hôpital Purpan, Toulouse, France; 220 Hôpital Saint-Louis AP-HP, Paris, France; 221 Centre Hospitalier Emile Roux, Le Puy-en-Velay, France; 222 Hôpital Bel-Air, Thionville, France; 223 Centre Hospitalier Universitaire Toulouse (IUCT), Toulouse, France; 224 Alrijne Hospital, Leiden, The Netherlands; 225 Policlinico of Padova, Padova, Italy; 226 Centre Hospitalier Universitaire de Nice (Hôpital Archet), Nice, France; 227 ISARIC Global Support Centre, Oxford, UK; 228 Universitair Ziekenhuis, Gent, Belgium; 229 INOVA Fairfax Medical Center, Fairfax, Virginia USA; 230 Hospital Universitario Dr Negrín, Las Palmas, Spain; 231 Hospital Professor Doutor Fernando Fonseca, Amadora, Portugal; 232 Clinica Las Condes, Santiago, Chile; 233 University Medical Center Groningen, Groningen, The Netherlands; 234 Centre Hospitalier Mont-de-Marsan, Mont-de-Marsan, France; 235 Rambam Hospital, Haifa, Israel; 236 Centre Hospitalier Andrée Rosemon, Cayenne, French Guiana; 237 Centre for Medical Informatics, The Usher Institute, University of Edinburgh, Edinburgh, UK; 238 Tallaght University Hospital, Dublin, Ireland; 239 Lions Gate Hospital, Vancouver, Canada; 240 Flevoziekenhuis, Almere, The Netherlands; 241 St James’s Hospital, Dublin, Ireland; 242 St Joseph’s Health Center, Sherbrooke, Canada; 243 Centre Hospitalier Universitaire d’Angers, Angers, France; 244 Houston Methodist Hospital, Houston, TX USA; 245 Oslo University Hospital, Oslo, Norway; 246 Cleveland Clinic, Ohio, OH USA; 247 Medical College of Wisconsin, Wisconsin, USA; 248 Hôpital de la Conception, Marseille, France; 249 Centre Hospitalier de Tourcoing, Tourcoing, France; 250 Centre Hospitalier De Chateaudun, Route De Jallans, 28200 Chateaudun, France; 251 Reinier de Graaf Gasthuis, Delft, The Netherlands; 252 Centre Hospitalier Universitaire Rennes (Hôpital Sud), Rennes, France; 253 Tohoku Medical and Pharmaceutical University, Sendai, Japan; 254 Plata Medical Foundation Private Community Hospital, Mar Del, Plata, Argentina; 255 Hospitales Puerta de Hierro, Jalisco, Mexico; 256 Canisius Wilhelmina Ziekenhuis, Nijmenjen, The Netherlands; 257 Centre Hospitalier Pierre Oudot, Bourgoin-Jallieu, France; 258 North York General Hospital, Toronto, Canada; 259 Hospital Universiti Sains Malaysia (Mix medical surgical ICU), Kota Bharu, Malaysia; 260 Adult ICU Saiful Anwar Hospital, Malang, Indonesia; 261 University of California San Francisco - Fresno, Fresno, USA; 262 Hospital Santa Maria, Centro Hospitalar Universitário Lisboa Norte, Amadora, Portugal; 263 Centre Hospitalier Techer, Calais, France; 264 Centre Hospitalier Régional et Universitaire de Tours, Tours, France; 265 Liverpool School of Tropical Medicine, Liverpool, UK; 266 University of Kansas Medical Center, Kansas, USA; 267 The Montreal Children’s Hospital, Montreal, Canada; 268 Vancouver General Hospital, Vancouver, Canada; 269 Ospedale San Gerardo, Monza, Italy; 270 Hôpital Foch, Suresnes, France; 271 Interdepartmental Division of Critical Care Medicine, University of Toronto, Toronto, Canada; 272 Bon Secours Hospital, Cork, Ireland; 273 Hospital Verge de la Cinta, Tortosa, Spain; 274 Hospital Escola da Universidade Federal de Pelotas, Pelotas, Brazil; 275 Hôpital Albert Calmette, Lille, France; 276 Saiseikai Senri Hospital, Tochigi, Japan; 277 Manipal Hospital Whitefield, Bangalore, India; 278 Centre Hospitalier de Pau, Pau, France; 279 Hôpital privé d’Antony, Antony, France; 280 São João Hospital Centre, Porto, Portugal; 281 San Pedro de Alcantara Hospital, Cáceres, Spain; 282 Beth Israel Deaconess Medical Center, Boston, USA; 283 Ochsner Clinic Foundation, New Orleans, USA; 284 Department of Internal Medicine No2, Lugansk State Medical University, Lugansk, Ukraine; 285 Klinikum Passau, Germant, Germany; 286 Cleveland Clinic Abu Dhabi, Abu Dhabi, United Arab Emirates; 287 University of Padua, Padua, Italy; 288 Children’s Hospital of Eastern Ontario, Ottawa, Canada; 289 Mater Misericordiae University, Dublin, Ireland; 290 Centre Hospitalier Henri Duffaut, Avignon, France; 291 The Baruch Padeh Medical Center Poriya, Tiberias, Israel; 292 Lankenau Institute of Medical Research, Wynnewood, USA; 293 The Hospital for Sick Children (SickKids), Toronto, Canada; 294 University Hospital of Tubingen, Tubingen, Germany; 295 CUB-Hôpital Erasme, Anderlecht, Belgium; 296 Permai Hospital, Johor, Malaysia; 297 Schools of Medicine, University of Michigan, Ann Arbor, USA; 298 Hospital Garcia de Orta, Almada, Portugal; 299 Department of Infectious Diseases, Institut Hospital del Mar d’Investigacions Mèdiques (IMIM), Infectious Pathology and Antimicrobial Research Group (IPAR), Hospital del Mar, Universitat Autònoma de Barcelona (UAB), CEXS-Universitat Pompeu Fabra, Barcelona, Spain; 300 Wexford General Hospital, Wexford, Ireland; 301 Baylor Scott, Dallas, USA; 302 Clinica Alemana DeSantiago, Santiago, Chile; 303 Centre Hospitalier du Pays d’Aix, Aix-en-Provence, France; 304 Institute of Microbiology and Infection, University of Birmingham, Birmingham, UK; 305 Department of Molecular and Clinical Cancer Medicine, University of Liverpool, Liverpool, UK; 306 Centre Hospitalier Universitaire Ambroise-Paré, Boulogne-Billancourt, France; 307 Roslin Institute, University of Edinburgh, Easter Bush, EH25 9RG Edinburgh, UK; 308 Grigore T, Popa University of Medicine and Pharmacy, Bucharest, Romania; 309 Erasmus Medical Centre, Rotterdam, The Netherlands; 310 University Children’s Hospital, University Medical Center Hamburg-Eppendorf, Hamburg, Germany; 311 Hospital de Curry Cabral - Internal Medicine, Lisbon, Portugal; 312 Centre Hospitalier Universitaire Toulouse (Larrey), Toulouse, France; 313 Hospital de Amor, Sao Paulo, Brazil; 314 Middlemore Hospital (Canties Manukan Health), Otahuhu, New Zealand; 315 Centre Hospitalier de Soissons, Soissons, France; 316 Red Deer Regional Hospital, Red Deer, Canada; 317 Big Data Institute, Department of Medicine, University of Oxford, Nuffield, Oxford, UK; 318 McLeod Healthcare System, Florence, USA; 319 Providence Saint John’s Health Centre, Santa Monica, USA; 320 Kluang Hospital, Johor, Malaysia; 321 Institute of Infection, Veterinary and Ecological Sciences, Faculty of Health and Life Sciences, NIHR Health Protection Research Unit, University of Liverpool, Liverpool, UK; 322 Kintampo Health Research Centre, Kintampo, Ghana; 323 AZ Maria Middelares, Gent, Belgium; 324 Drammen Hospital, Drammen, Norway; 325 Universidad de Las Américas, Quito, Ecuador; 326 Lancaster General Health, Pennsylvania , USA; 327 PICU Saiful Anwar Hospital, Malang, Indonesia; 328 Nagoya University Hospital, Nagoya, Japan; 329 Long COVID India - Terna Specialty Hospital and Research Centre, Mumbai, India; 330 Centre Hospitalier Le Mans, Le Mans, France; 331 Medical Research Council, University of Glasgow Centre for Virus Research, Glasgow, UK; 332 Sultanah Bahiyah Hospital, Kedah, Malaysia; 333 Tuanku Ja’afar, Negeri Sembilan, Seremban, Malaysia; 334 University of Alabama at Birmingham Hospital, Birmingham, USA; 335 Hokkaido University Hospital, Hokkaido, Japan; 336 Fukuoka University, Fukuoka, Japan; 337 US NHLBI PETAL Network, Boston, USA; 338 University of California - San Francisco (UCSF), San Francisco, USA; 339 St. Joseph’s Healthcare Hamilton, Hamilton, Canada; 340 Virology Reference Department, National Infection Service, Public Health England, Colindale Avenue, London, UK; 341 La Paz Hospital, Madrid, Spain; 342 Centre Hospitalier Annecy Genevois, Annecy, France; 343 Hippokration Hospital, Thessaloniki, Greece; 344 RSUP Fatmawati, South Jakarta, Indonesia; 345 Hiroshima University, Hiroshima, Japan; 346 Mie University Hospital, Tsu, Japan; 347 Hospital Aleman, Buenos Aires, Argentina; 348 Oxford University (ISARIC4C), Oxford, UK; 349 Mills Memorial Hospital, Terrace, Canada; 350 Raja Perempuan Zainab II Hospital, Kelantan, Malaysia; 351 Hospital Nuestra Señora de Gracia, Zaragoza, Spain; 352 Kyungpook National University Hospital, Daegu, South Korea; 353 Consortium IMGEN, Piaseczno, Poland; 354 Tawau Hospital, Sabah, Malaysia; 355 Melaka Hospital, Melaka, Malaysia; 356 Princess Margaret Hospital, Kwai Hung, China; 357 Nationwide Children’s Hospital, Columbus, USA; 358 Washington University in St. Louis, St Louis, Missouri USA; 359 Intensive Care Unit, Royal Infirmary Edinburgh, Edinburgh, UK; 360 University of Oklahoma Health Sciences Center, Oklahoma, USA; 361 Groupe Hospitalier Diaconesses Croix Saint-Simon, Paris, France; 362 Hospital for Tropical Diseases, Ho Chi Minh City, Vietnam; 363 Unity Health Toronto, Toronto, Canada; 364 McMaster University, Hamilton, Canada; 365 Lahad Datu Hospital, Sabah, Malaysia; 366 Department of Pharmacology, University of Liverpool, Liverpool, UK; 367 University Hospital of North Norway, Tromso, Norway; 368 Keimyung University Dong San Hospital, Daegu, South Korea; 369 Kimitsu Chuo Hospital, Chiba, Japan; 370 Nuffield Department of Medicine, Peter Medawar Building for Pathogen Research, University of Oxford, Oxford, UK; 371 Obihiro-Kosei General Hospital, Obihiro, Japan; 372 Marmara University Hospital, Istanbul, Turkey; 373 University Health Network, Toronto, Canada; 374 Apollo Hospitals Chennai, Chennai, India; 375 Hartford HealthCare, Hartford, USA; 376 University Airlangga Hospital (Paediatric), Surabaya, Indonesia; 377 Royal Alexandra Hospital, Edmonton, Canada; 378 Centre Hospitalier Alpes-Leman, Contamine-sur-Arve, France; 379 Centre Hospitalier Universitaire Toulouse (Rangueil), Toulouse, France; 380 Prof Dr R. D. Kandou Central Hospital (Adult), Manado, Indonesia; 381 Université de Paris, Paris, France; 382 Centre Hospitalier Universitaire de Nîmes, Nîmes, France; 383 The Roslin Institute, University of Edinburgh, Edinburgh, UK; 384 Wellington Regional Hospital, Wellington, New Zealand; 385 University of Alberta Adult ICU, Edmonton, Canada; 386 Centre Hospitalier Universitaire de Montpellier, Montpellier, France; 387 Centre Hospitalier Universitaire de Poitiers, Poitiers, France; 388 Queen Elizabeth Hospital, Sabah, Malaysia; 389 Severance Hospital, Seoul, South Korea; 390 Division of Informatics, Imaging and Data Science, School of Health Sciences, Faculty of Biology, Medicine and Health, Centre for Health Informatics, University of Manchester, Manchester Academic Health Science Centre, Manchester, UK; 391 Hôpital Henri-Mondor, Créteil, France; 392 University Institute of Cardiology and Respirology, Quebec, Canada; 393 Sultanah Nur Zahirah Hospital, Terengganu, Malaysia; 394 Centre Hospitalier Universitaire Gabriel Montpied, Clermont-Ferrand, France; 395 Institute of TB and Lung Diseases, Warsaw, Poland; 396 Waitemata District Health Board, Auckland, New Zealand; 397 Nottingham University Hospitals NHS Trust, Nottingham, UK; 398 Angeles University Foundation Medical Center, Angeles, Philippines; 399 Malawi-Liverpool Wellcome Trust, Blantyre, Malawi; 400 Saiseikai Utsunomiya Hospital, Tochigi, Japan; 401 University of Florida, Gainesville, USA; 402 Instituto Nacional del Niño San Borja, Lima, Peru; 403 Hospital de Clínicas, Buenos Aires, Argentina; 404 Hospital Emergencia Ate Vitarte, Lima, Peru; 405 Port Macquarie Base Hospital, Port Macquarie, Australia; 406 Klinik und Poliklinik für Innere Medizin II, University Hospital Regensburg, Kiel, Germany; 407 William Osler Health Sciences System - Etobicoke General Hospital, Toronto, Canada; 408 Prof Dr R. D. Kandou Central Hospital (Paediatric), Manado, Indonesia; 409 Hôpital Louis-Mourier, Colombes, France; 410 Hospital Vila Franca de Xira, Lisbon, Portugal; 411 Alberta Children’s Hospital, Calgary, Canada; 412 Department of Intensive Care Medicine, Multidisciplinary Intensive Care Research Organisation (MICRO), St. James’s Hospital, Dublin, Ireland; 413 Centre Hospitalier de Colmar, Colmar, France; 414 Emergency Department. Hospital, Universitario La Paz - IdiPAZ, Madrid, Spain; 415 Kingston Health Sciences Centre, Kingston, Canada; 416 International Islamic University Malaysia Medical Centre (IIUMMC), Pahang, Malaysia; 417 Waikato Hospital, Hamilton, New Zealand; 418 Auckland City Hospital, (DCCM 82), Auckland, New Zealand; 419 Mount Sinai Hospital, Toronto, Canada; 420 London Health Sciences Centre, London, Canada; 421 Wellcome Centre for Human Genetics, University of Oxford, Oxford, UK; 422 Centre Hospitalier de Cahors, Cahors, France; 423 Institute of Genetics and Molecular Medicine, MRC Human Genetics Unit, MRC, University of Edinburgh, Edinburgh, UK; 424 Centre Hospitalier Universitaire Rouen (Hôpital Charles Nicolle), Rouen, France; 425 Institute of Infection, Veterinary and Ecological Sciences, University of Liverpool, Liverpool, UK; 426 Hospital Clinic, Barcelona, Spain; 427 Tengku Ampuan Afzan Hospital, Pahang, Malaysia; 428 Florence Nightingale Faculty of Nursing, Midwifery and Palliative Care, Care for Long Term Conditions Division, King’s College London, London, UK; 429 Faculty of Medicine, University of British Columbia, Vancouver, Canada; 430 Baystate MC, Springfield, USA; 431 University Hospital Northern British Columbia, Prince George, Canada; 432 St-Pierre University Hospital, Brussels, Belgium; 433 Faculty of Medicine, Universitas Indonesia, Cipto Mangunkusumo Hospital, Kota Depok, Indonesia; 434 Queen Mary Hospital, Pok Fu Lam, China; 435 Hôpital Pellegrin, Bordeaux, France; 436 Irish Critical Care Critical Clinical Trials Network, Dublin, Ireland UK; 437 Siriraj Piyamaharajkarun Hospital (SiPH), Bangkok, Thailand; 438 Oregon Health, Salem, USA; 439 Cleveland Clinic, Abu Dhabi, United Arab Emirates; 440 Division of Infection and Immunity, University College London, London, UK; 441 Department of Children’s Infectious Diseases, Warsaw, Poland; 442 Dr Sardjito Government Hospital (Paediatric), Yogyakarta, Indonesia; 443 RSUD Pasar Minggu, South Jakarta, Indonesia; 444 Mercy Hospital, Cork, Ireland; 445 Legacy Emanuel Medical Center, Portland, USA; 446 Kyung Pook National University Chilgok Hospital, Daegu, South Korea; 447 Department of Emergency and Critical Care Medicine, Graduate School of Biomedical and Health Sciences, Hiroshima University, Hiroshima, Japan; 448 Instituto do Coração da Universidade de São Paulo (INCOR), São Paulo, Brazil; 449 Misericordia Community Hospital, Edmonton, Canada; 450 Department of Medical Microbiology and Infection Control, Franciscus Gasthuis & Vlietland, Rotterdam, The Netherlands; 451 Joseph Brant Hospital, Burlington, Canada; 452 National Heart and Lung Institute, Imperial College London, London, UK; 453 Tufts Medical Centre, Boston, USA; 454 Mayo Clinic School of Medicine, Arizona, USA; 455 All India Institute of Medical Sciences (AIIMS), Rishikesh, India; 456 Hospital General San Francisco, Quito, Ecuador; 457 Auckland City Hospital (CVICU), Auckland, New Zealand; 458 Hospital Beatriz Ângelo, Loures, Portugal; 459 Niagara Health, Niagara, Canada; 460 Centre Hospitalier de Périgueux, Périgueux, France; 461 University Hospital Ostrava, Ostrava-Poruba, Czechia; 462 Humber River Hospital, Toronto, Canada; 463 Maastricht University Medical Centre, Maastricht, The Netherlands; 464 North Estonia Medical Centre, Tallin, Estonia; 465 RSUD Dr. Soetomo, Surabaya, Indonesia; 466 Baylor University Medical Centre, Dallas, USA; 467 Apollo Hospitals Chennai, Chennai, Tamil Nadu India; 468 Ministry of Health, and D’Or Institute of Research and Education (IDOR), National Institute of Infectious Disease Evandro Chagas, Oswaldo Cruz Foundation (INI-FIOCRUZ), Rio de Janeiro, Gaspar Viana Pavilion, Brazil; 469 Network for Improving Critical care Systems and Training, Colombo, Sri Lanka; 470 Clinic of Anesthesiology and Intensive Care, North Estonia Medical Centre, Tallinn, Estonia; 471 Hospital de Abrantes - ICU, Abrantes, Portugal; 472 Hôpital Européen Marseille, Marseille, France; 473 Centre Hospitalier Agen-Nérac, Agen, France; 474 Vall d’Hebron Institute of Research, Barcelona, Spain; 475 University of Manitoba, Manitoba, Canada; 476 Universidad de La Sabana, Chia, Colombia; 477 The Center for Diagnosis, Santo Domingo, Dominican Republic; 478 CHU Carémeau, Nimes, France; 479 Royal Melbourne Hospital, Melbourne, Australia; 480 Hôpital Laënnec - site de Quimper, Quimper, France; 481 Fundación Cardiovascular de Colombia, Floridablanca, Colombia; 482 Hospital Universitari Sant Joan D’Alacant, Alicante, Spain; 483 Department of Pediatrics and Virology, St Mary’s Medical School Bldg, Imperial College London, London, UK; 484 Department of Infectious Diseases I, Carol Davila University of Medicine and Pharmacy, Bucharest, Romania; 485 Centro Hospitalar Universitário do Algarve, Portimão, Portugal; 486 RSPI Prof Dr Sulianti Saroso, Jakarta, Indonesia; 487 The Heart Hospital Baylor Plano, Plano, USA; 488 Gelre Hospitals, Zutphen, The Netherlands; 489 Krankenhaus Barmherzige Br, Regensburg, Germany; 490 Baylor AllSaints Medical Centre, Fort Worth, USA; 491 Sozialmedizinisches Zentrum Sud, Vienna, Austria; 492 Institute of Infection, Veterinary and Ecological Sciences, Faculty of Health and Life Sciences, University of Liverpool, Liverpool, UK; 493 Centro Hospitalar de Leiria, Leiria, Portugal; 494 Institute of Translational Medicine, University of Liverpool, Liverpool, Merseyside, UK; 495 Tohoku University, Sendai, Japan; 496 Hyogo Prefectural Kakogawa Medical Center, Hyogo, Japan; 497 Tokyo Metropolitan Tama Medical Center, Tokyo, Japan; 498 Pamela Youde Nethersole Eastern Hospital, Chai Wan, China; 499 Grand River Hospital, Kitchener, Canada; 500 Hospital Sungai Buloh, Ministry of Health, Selangor, Malaysia; 501 Seoul National University Bundang Hospital, Seoul, South Korea; 502 Hospital Universitário Clementino Fraga Filho, Rio de Janeiro, Brazil; 503 Baerum Sykehus, Gjettum, Norway; 504 National Institute for Infectious Diseases Matei Bals, Bucharest, Romania; 505 Carol Davila University of Medicine and Pharmacy, Bucharest, Romania; 506 Division of Structural Biology, The Wellcome Centre for Human Genetics, University of Oxford, Headington, Oxford, OX3 7BN UK; 507 Sturgeon Community Hospital, St Albert, Canada; 508 Department of Medicine, University of Cambridge, Cambridge, Cambridgeshire UK; 509 University Hospital in Krakow, Krakow, Poland; 510 Centre Hospitalier Universitaire Grenoble-Alpes_FU, Grenoble, France; 511 Hospital das Clinicas da Faculdade de Medicina da Universidade de Sao Paulo, Sao Paulo, Brazil; 512 Kyoto Medical Centre, Kyoto, Japan; 513 Yokohama City University Medical Center, Yokohama, Japan; 514 Fatmawati Hospital, Jakarta, Indonesia; 515 Virus Reference Department, National Infection Service, Blood Borne Virus Unit, Public Health England, London, UK; 516 Complexo Hospitalar Dr. Clementino Fraga, João Pessoa city, Brazil; 517 Centre Hospitalier Louis Raffalli, Manosque, France; 518 University of Nebraska Medical Center, Omaha, USA; 519 Division of Critical Care, University of Cape Town and Groote Schuur Hospital, Cape Town, South Africa; 520 Clínica Internacional, Lima, Peru; 521 Albury Wodonga Health, Albury, Australia; 522 Hôpital Robert-Debré AP-HP, Paris, France; 523 Dunedin Public Hospital, Dunedin, New Zealand; 524 ADRZ, Amsterdam, The Netherlands; 525 Meander Medical Centre, Amersfoort, The Netherlands; 526 Adrz, Goes, The Netherlands; 527 Noordwest-Ziekenhuisgroep, DenHelder, The Netherlands; 528 Grey Nun’s Community Hospital, Edmonton, Canada; 529 Beatrix ziekenhuis, Gorinchem, The Netherlands; 530 Royal Columbian Hospital, Vancouver, Canada; 531 Department of Intensive Care, Franciscus Gasthuis, Rotterdam, The Netherlands; 532 Queen Elizabeth Hospital, Yau Ma Tei, China; 533 Kyoto Prefectural University of Medicine, Kyoto, Japan; 534 Kouritu Tousei Hospital, Seto City, Japan; 535 MedStar Washington Hospital Centre, Washington, USA; 536 Clinica Pasteur National - University of Comahue, Neuquén, Argentina; 537 Sultanah Aminah Hospital, Johor, Malaysia; 538 National Infection Service, Public Health England, London, UK; 539 Mazankowski Heart Institute, Edmonton, Canada

**Keywords:** Biomedical engineering, Epidemiology, Risk factors, Infectious diseases

## Abstract

By September 2022, more than 600 million cases of SARS-CoV-2 infection have been reported globally, resulting in over 6.5 million deaths. COVID-19 mortality risk estimators are often, however, developed with small unrepresentative samples and with methodological limitations. It is highly important to develop predictive tools for pulmonary embolism (PE) in COVID-19 patients as one of the most severe preventable complications of COVID-19. Early recognition can help provide life-saving targeted anti-coagulation therapy right at admission. Using a dataset of more than 800,000 COVID-19 patients from an international cohort, we propose a cost-sensitive gradient-boosted machine learning model that predicts occurrence of PE and death at admission. Logistic regression, Cox proportional hazards models, and Shapley values were used to identify key predictors for PE and death. Our prediction model had a test AUROC of 75.9% and 74.2%, and sensitivities of 67.5% and 72.7% for PE and all-cause mortality respectively on a highly diverse and held-out test set. The PE prediction model was also evaluated on patients in UK and Spain separately with test results of 74.5% AUROC, 63.5% sensitivity and 78.9% AUROC, 95.7% sensitivity. Age, sex, region of admission, comorbidities (chronic cardiac and pulmonary disease, dementia, diabetes, hypertension, cancer, obesity, smoking), and symptoms (any, confusion, chest pain, fatigue, headache, fever, muscle or joint pain, shortness of breath) were the most important clinical predictors at admission. Age, overall presence of symptoms, shortness of breath, and hypertension were found to be key predictors for PE using our extreme gradient boosted model. This analysis based on the, until now, largest global dataset for this set of problems can inform hospital prioritisation policy and guide long term clinical research and decision-making for COVID-19 patients globally. Our machine learning model developed from an international cohort can serve to better regulate hospital risk prioritisation of at-risk patients.

## Introduction

### Clinical background

On the last day of 2019, the WHO received information about 44 cases of pneumonia-like disease in Wuhan city, China^1^. By 5 September 2022, more than 600 million cases of SARS-CoV-2 infection had been reported across all continents, regions, and most countries, resulting in nearly 6.5 million deaths^2^.

COVID-19, the disease caused by infection with SARS-CoV-2, has a high mortality rate in hospitalised patients with deaths predominantly caused by respiratory failure^3^. It continues to this day to be a challenging global pandemic with significant morbidity and mortality^4^. As Knight et al.^5^ indicate, prognostic models that can predict outcomes among COVID-19 patients can be used to support clinical decision-making regarding hospital treatment and prioritisation. One such score is the 4C score that includes data about patient comorbidity, abnormal physiology, and inflammation using routinely measured data, bedside observations, and biochemistry tests^6^. While in most cases COVID-19 is a mild illness, those at highest risk of death and severe complications usually are hospitalised some time after onset^7^.

Pulmonary embolism (PE) is among the most severe and preventable complications of COVID-19 characterized by increased D-dimer levels and high thrombosis risk that has been repeatedly reported across different countries^8^. Studies suggest PE incidence rates above 15% in the ICU for COVID-19 patients and early recognition of its risk factors can help in identifying urgent treatment with anticoagulation therapy to those most in clinical need^4,9^. Recent international studies additionally suggest COVID-19 as a key risk factor for pulmonary embolism both in the short- and long-term^9,10^. Existing PE prediction models are limited in part because they were developed for non-COVID-19 patients and traditional risk factors for PE may not be as predictive. If risk models can be developed for assessing occurrence of PE in COVID-19 patients across different countries, that can be an important step forward in preventing this serious complication of COVID-19, especially given the current epidemiological situation^9^.

As for risk factors that contribute the most to the occurrence of mortality and pulmonary embolism in COVID-19 patients, age has been established as the dominant predictor of mortality^11^. Furthermore, studies have described other risk factors of COVID mortality such as cardiovascular disease, chronic respiratory disease, diabetes, hypertension, smoking, and obesity^12^.

### Technical background

Machine learning has been applied to different COVID-19 related questions. Large amounts of patient data are being generated during the COVID-19 pandemic which can be useful for predictive modelling. Using machine learning with large amounts of complex patient data could generate accurate and patient-specific predictions and assist clinicians.

Previous research includes^13^ exploring in-hospital mortality with logistic regression on just 191 patients and^14^ have followed with multi-center validation with 299 patients for internal training and 145 patients for external validaton.^15^ have looked at regression-based predictions of all-cause mortality with hospital admission time as a predictor and using hazard models yet their results have also been limited due to a smaller dataset restricting generalisability. All of these studies have used a combination of demographics, comorbidities, symptoms, laboratory tests, and self-reported onset times.

In this study, we investigated how pulmonary embolism and all-cause mortality vary across subgroups of a large and international cohort. We also show how predictive certain clinical factors gathered from patients with COVID-19 can be to the respective outcomes. In studies looking at predicting thromboembolism more broadly, a defining limitation for impactful and generalisable application of machine learning methods has been a small patient sample and a lack of systematic comparison of algorithms^16^. Applying a diverse set of methods to one of the largest and most diverse datasets on hospitalised patients with COVID-19 can help find the best mechanism for risk prioritisation of patients in a timely way and may help reduce mortality and risk of PE in those with COVID-19.

## Results

Variable distributions can be seen in Tables 1, 2, 3, and 4. A detailed collection of figures for variable distribution across age groups can be found in the Supplementary.

**Table 1:** Baseline characteristics stratified by occurrence of PE (median and IQR used for lab measurements).

Characteristic	PE (N = 5656)		Non-PE	
	Missing (%)	Mean (SD)/count (%)	Missing (%)	Mean (SD)/count (%)
Age	0.8	62.6 (15.6)	4.1	56.4 (20.9)
Sex (male)	0.1	3733 (66.0)	2.1	385,038 (48.4)
Alpha variant (post)	–	3,733 (66.0)	2.1	385,038 (48.4)
Ethnicity				
White	–	309 (5.5)	–	12,030 (1.5)
South Asian	–	32 (0.6)	–	9224 (1.2)
Malay	–	0 (0.0)	–	3,812 (0.5)
Latin American	–	24 (0.4)	–	2719 (0.3)
Other	–	5171 (91.4)	–	757,656 (95.3)
Country				
South Africa	–	0 (0.0)	–	432,596 (54.4)
United Kingdom	–	4076 (72.1)	–	269,073 (33.9)
Spain	–	577 (10.2)	–	14,764 (1.9)
Norway	–	15 (0.3)	–	7448 (0.9)
Other	–	988 (17.4)	–	71,853 (9.0)
Country income				
High	–	5513 (97.5)	–	323,889 (40.8)
Upper middle	–	72 (1.3)	–	449,782 (56.6)
Lower middle	–	71 (1.2)	–	20,210 (2.5)
Low	–	0 (0.0)	–	918 (0.1)
Region				
Sub-saharan Africa	–	0 (0.0)	–	433,522 (54.5)
Europe and Central Asia	–	5,264 (93.1)	–	314,416 (39.6)
South Asia	–	49 (0.9)	–	17,413 (2.2)
East Asia	–	39 (0.7)	–	10,421 (1.3)
North America	–	195 (3.4)	–	9,666 (1.2)
Other	–	107 (1.9)	–	9,498 (1.2)
Comorbidities				
AIDS/HIV	15.5	19 (0.3)	28.3	27,895 (3.5)
Asthma	10.6	644 (11.4)	26.4	51,714 (6.5)
Chronic cardiac disease	8.3	1002 (17.7)	26.2	80,348 (10.1)
Chronic haematological	11.2	171 (3.0)	64.7	10,275 (1.3)
Chronic kidney disease	8.8	516 (9.1)	27.0	46,054 (5.8)
Chronic neurological	9.6	369 (6.5)	63.8	28,648 (3.6)
Chronic pulmonary	7.9	707 (12.5)	26.5	52,006 (6.5)
Dementia	9.8	214 (3.8)	64.5	27,374 (3.4)
Diabetes	10.4	1196 (21.1)	24.5	152,728 (19.2)
Hypertension	10.3	2219 (39.2)	27.1	226,285 (28.5)
Liver disease	7.3	169 (3.0)	62.3	8960 (1.1)
Malignant neoplasm	8.7	508 (9.0)	27.1	27,811 (3.5)
Malnutrition	15.1	59 (1.0)	67.3	5,582 (0.7)
Obesity	16.2	1,167 (20.6)	57.0	53,227 (6.7)
Rheumatologic	9.9	485 (8.6)	64.9	27,954 (3.5)
Smoking	45.5	1317 (23.3)	71.5	74,205 (9.3)

PE here also includes positive cases of deep vein thrombosis and thromboembolism.

**Table 2:** Baseline characteristics stratified by occurrence of PE (continued).

Characteristic	PE (N = 5656)		Non-PE	
	Missing (%)	Mean (SD)/count (%)	Missing (%)	Mean (SD)/count (%)
Symptoms				
Symptomatic	2.3	5400 (95.5)	63.4	276,645 (34.8)
Abdominal pain	19.1	310 (5.5)	70.6	21,510 (2.7)
Confusion	14.4	624 (11.0)	70.5	45,559 (5.7)
Bleeding	19.0	95 (1.7)	70.8	4307 (0.5)
Chest pain	17.0	1,058 (18.7)	70.4	32,811 (4.1)
Conjunctivitis	23.2	15 (0.3)	72.0	1012 (0.1)
Cough	10.7	3434 (60.7)	67.7	153,548 (19.3)
Diarrhoea	14.1	886 (15.7)	69.9	39,718 (5.0)
Ear pain	43.4	7 (0.1)	77.0	765 (0.1)
Fatigue	18.5	2134 (37.7)	70.9	93,637 (11.8)
Headache	20.0	510 (9.0)	71.8	26,815 (3.4)
Fever	10.6	3,029 (53.6)	67.8	146,248 (18.4)
Lost sense of smell	24.7	403 (7.1)	77.4	12,736 (1.6)
Lost sense of taste	28.3	438 (7.7)	77.9	14,767 (1.9)
Lymphadenopathy	23.7	33 (0.6)	72.9	1264 (0.2)
Muscle/joint pain	20.5	903 (16.0)	71.8	40,627 (5.1)
Runny nose	26.0	111 (2.0)	73.0	8,244 (1.0)
Seizures	18.1	19 (0.3)	71.3	2764 (0.3)
Severe dehydration	64.9	245 (4.3)	85.3	13,732 (1.7)
Shortness of breath	6.8	4205 (74.3)	67.5	156,078 (19.6)
Skin rash	21.5	76 (1.3)	71.5	5,771 (0.7)
Sore throat	25.8	225 (4.0)	72.9	16,500 (2.1)
Vomiting	17.3	736 (13.0)	69.9	43,149 (5.4)
Wheezing	22.7	278 (4.9)	71.7	14,474 (1.8)
Lab measurements				
D-dimer ($\mu$g/mL)	92.0	1.1 (0.5, 2.5)	98.6	0.7 (0.4, 1.3)
ALT (IU/L)	64.1	36.0 (22.0, 60.0)	86.0	27.0 (17.0, 45.0)
Bilirubin ($\mu$mol/L)	70.5	11.0 (8.0, 15.6)	85.7	9.0 (7.0, 14.0)
CRP (mg/L)	35.7	115.0 (59.0, 191.8)	79.7	74.9 (29.0, 143.0)
Lymphocytes (10$^{3}\mu$L)	33.2	0.9 (0.6, 1.3)	78.4	0.9 (0.6, 1.3)
Neutrophils (10$^{9 \text L}$)	33.2	7.1 (4.8, 10.1)	78.4	5.5 (3.8, 8.2)
Platelets (10$^{9 \text L}$)	57.7	13.0 (11.5, 14.7)	88.0	12.8 (11.2, 14.3)
Blood Urea Nitrogen (mmol/L)	44.1	6.5 (4.7, 9.7)	79.8	6.4 (4.5, 10.0)
White Blood Cells (10$^{9 \text L}$)	30.0	8.8 (6.2, 12.1)	77.0	7.2 (5.3, 10.1)
Vital signs				
Diastolic BP (mmHg)	6.9	75.5 (14.8)	64.7	74.8 (15.2)
Systolic BP (mmHg)	6.8	130.2 (23.2)	64.7	130.3 (24.5)
Heart rate (bpm)	7.4	96.3 (21.1)	65.4	92.0 (21.5)
Oxygen saturation (%)	6.8	90.7 (11.3)	64.7	93.4 (9.3)
Respiratory rate (brpm)	10.1	25.1 (8.0)	65.7	22.8 (7.0)
Temperature ($^{\circ }$C)	7.1	37.2 (1.1)	64.2	37.2 (1.0)
Outcome				
Discharge	–	3,492 (61.7)	–	519,423 (65.4)
Death	–	1297 (22.9)	–	162,091 (20.4)
Other	–	531 (15.4)	–	54,930 (14.2)

PE here also includes positive cases of deep vein thrombosis and thromboembolism.

**Table 3:** Baseline characteristics stratified by death (median and IQR used for lab measurements).

Characteristic	Death (N = 163,388)		No death	
	Missing (%)	Mean (SD)/count (%)	Missing (%)	Mean (SD)/count (%)
Age	0.8	67.5 (16.1)	2.8	53.2 (21.0)
Sex (male)	0.2	87,679 (53.7)	0.5	282,993 (48.0)
Country				
South Africa	–	96,965 (59.3)	–	332,012 (56.3)
United Kingdom	–	54,540 (33.4)	–	193,780 (32.9)
Spain	–	123 (0.1)	–	7486 (1.3)
Norway	–	41 (< 0.1)	–	7216 (1.2)
Other	–	11,719 (7.2)	–	48,992 (8.3)
Country Income				
High	–	59,531 (36.4)	–	231,101 (39.2)
Upper Middle	–	98,064 (60.0)	–	345,013 (58.5)
Lower Middle	–	5725 (3.5)	–	12,654 (2.1)
Low	-	68 (<0.1)	–	717 (0.1)
Region				
Sub-saharan Africa	–	97,033 (59.4)	–	332,730 (56.4)
Europe and Central Asia	–	57,129 (35.0)	–	222,268 (37.7)
South Asia	–	5232 (3.2)	–	11,491 (1.9)
East Asia	–	678 (0.4)	–	8,322 (1.4)
North America	–	2099 (1.3)	–	6776 (1.1)
Other	–	1237 (0.8)	–	7999 (1.4)
Comorbidities				
AIDS/HIV	27.3	6,203 (3.8)	26.2	20,828 (3.5)
Asthma	25.2	10,425 (6.4)	24.6	40,402 (6.9)
Chronic Cardiac Disease	24.4	26,400 (16.2)	24.6	52,337 (8.9)
Chronic Haematological	65.1	2,901 (1.8)	65.8	7,095 (1.2)
Chronic Kidney Disease	25.6	16,693 (10.2)	25.3	28,559 (4.8)
Chronic Neurological	64.1	8,071 (4.9)	64.7	19,981 (3.4)
Chronic Pulmonary	25.0	16,224 (9.9)	24.7	34,869 (5.9)
Dementia	65.1	10,261 (6.3)	65.5	16,740 (2.8)
Diabetes	21.3	45,593 (27.9)	23.1	104,810 (17.8)
Hypertension	24.7	64,098 (39.2)	25.4	157,991 (26.8)
Immunosuppression	79.6	1364 (0.8)	81.9	3728 (0.6)
Liver Disease	62.4	2262 (1.4)	63.3	6269 (1.1)
Malignant Neoplasm	25.3	9036 (5.5)	25.5	17,719 (3.0)
Malnutrition	67.0	1717 (1.1)	67.1	3692 (0.6)
Obesity	55.1	11,176 (6.8)	56.3	39,153 (6.6)
Rheumatologic	65.2	7333 (4.5)	65.9	20,359 (3.5)
Smoking	72.5	17,655 (10.8)	71.5	53,764 (9.1)
Tuberculosis	55.5	2725 (1.7)	55.6	8688 (1.5)

**Table 4:** Baseline characteristics stratified by death (continued).

Characteristic	Death (N = 163,388)		No death	
	Missing (%)	Mean (SD)/count (%)	Missing (%)	Mean (SD)/count (%)
Symptoms				
Symptomatic	63.9	57,578 (35.2)	61.7	211,379 (35.9)
Abdominal Pain	71.4	3,452 (2.1)	69.1	17,537 (3.0)
Confusion	69.7	16,267 (10.0)	69.3	29,022 (4.9)
Bleeding	71.3	1099 (0.7)	69.3	3202 (0.5)
Chest Pain	71.4	4554 (2.8)	68.7	27,581 (4.7)
Conjunctivitis	72.8	220 (0.1)	70.6	792 (0.1)
Cough	68.2	31,110 (19.0)	66.0	117,745 (20.0)
Diarrhoea	70.5	6860 (4.2)	68.3	31,776 (5.4)
Ear Pain	76.7	100 (0.1)	75.4	629 (0.1)
Fatigue	71.7	19,510 (11.9)	69.4	70,064 (11.9)
Headache	73.4	2469 (1.5)	70.1	22,228 (3.8)
Fever	68.0	29,252 (17.9)	66.3	111,358 (18.9)
Lost Sense of Smell	79.6	1131 (0.7)	75.6	10,998 (1.9)
Lost Sense of Taste	80.2	1671 (1.0)	76.2	12,648 (2.1)
Lymphadenopathy	72.8	311 (0.2)	71.8	843 (0.1)
Muscle/Joint Pain	73.5	5706 (3.5)	70.1	33,181 (5.6)
Runny Nose	74.1	865 (0.5)	71.5	6,058 (1.0)
Seizures	71.2	573 (0.4)	70.1	2,109 (0.4)
Severe Dehydration	85.5	4,400 (2.7)	84.2	9,369 (1.6)
Shortness of Breath	67.6	37,074 (22.7)	65.9	116,392 (19.7)
Skin Rash	72.1	1593 (1.0)	70.1	4028 (0.7)
Sore Throat	74.0	2115 (1.3)	71.4	12,826 (2.2)
Vomiting	70.6	6506 (4.0)	68.4	35,578 (6.0)
Wheezing	72.3	4077 (2.5)	70.2	9779 (1.7)
Lab Measurements				
D-dimer ($\mu$g/mL)	99.1	1.1 (0.5, 2.5)	98.6	0.7 (0.4, 1.3)
ALT (IU/L)	85.3	36.0 (22.0, 60.0)	85.2	27.0 (17.0, 45.0)
Bilirubin ($\mu$mol/L)	84.3	11.0 (8.0, 15.6)	85.2	9.0 (7.0, 14.0)
CRP (mg/L)	78.9	115.0 (59.0, 191.8)	78.3	74.9 (29.0, 143.0)
Lymphocytes (10$^3\mu$L)	78.3	0.9 (0.6, 1.3)	76.7	0.9 (0.6, 1.3)
Neutrophils (10$^{9 \text L}$)	78.3	7.1 (4.8, 10.1)	76.7	5.5 (3.8, 8.2)
Platelets (10$^{9 \text L}$)	86.7	13.0 (11.5, 14.7)	87.3	12.8 (11.2, 14.3)
Blood Urea Nitrogen (mmol/L)	78.9	6.5 (4.7, 9.7)	78.5	6.4 (4.5, 10.0)
White Blood Cells (10$^{9 \text L}$)	76.6	8.8 (6.2, 12.1)	75.3	7.2 (5.3, 10.1)
Vital Signs				
Diastolic BP (mmHg)	63.1	72.9 (16.3)	64.3	75.5 (14.8)
Systolic BP (mmHg)	63.0	130.0 (26.3)	64.2	131.0 (24.0)
Heart Rate (bpm)	63.2	92.5 (22.3)	65.3	91.8 (21.2)
Oxygen Saturation ($\%$)	63.3	91.3 (10.2)	64.3	93.8 (9.2)
Respiratory Rate (brpm)	63.4	24.4 (7.7)	64.7	22.3 (6.6)
Temperature ($^{\circ }$C)	63.1	37.2 (1.1)	63.9	37.2 (1.0)
PE				
Yes	99.2	1365 (0.8)	99.3	4291 (0.7)

PE here also includes positive cases of deep vein thrombosis and thromboembolism.

**Figure 1:**
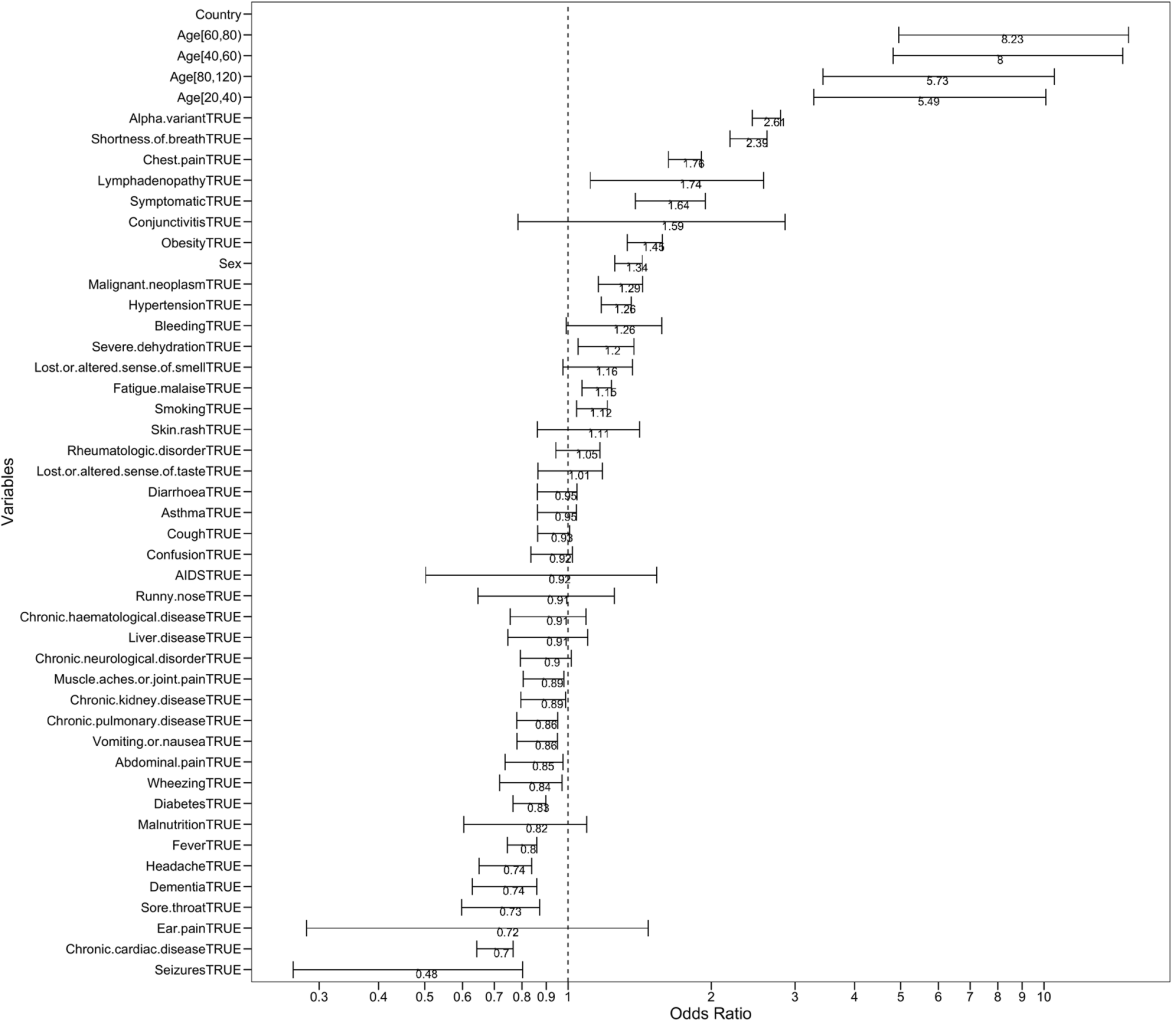
Adjusted odds ratios for PE with 95% confidence intervals.

**Figure 2:**
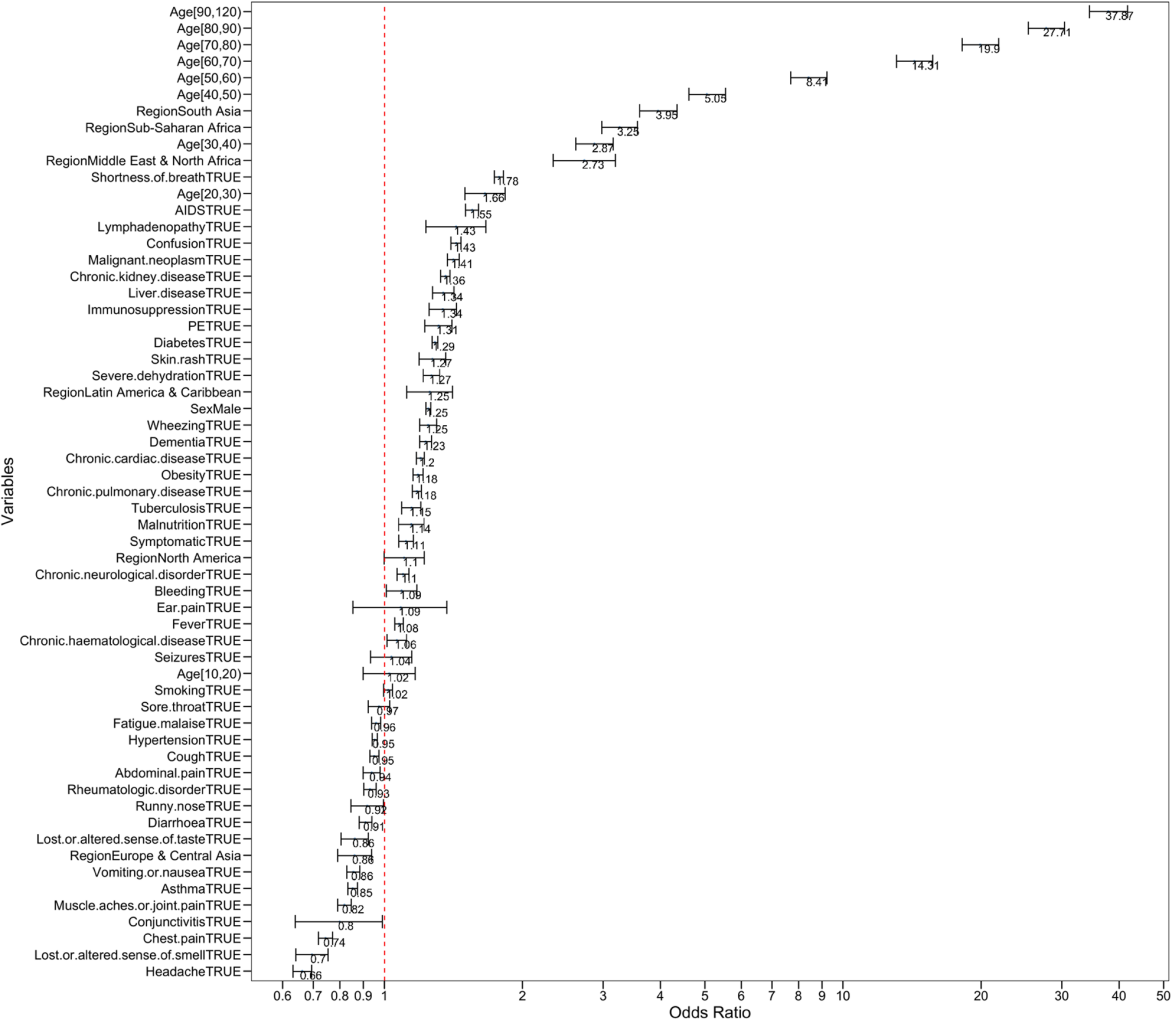
Adjusted odds ratios for death with 95% confidence intervals.

Several variables were highly correlated with PE and death (Supplementary Figures 3 and 4, Tables II and III). Multivariable logistic regression shows high association of country, age, alpha variant, and certain symptoms with PE and death (Figs. 1 and 2). Tables with *p*-values are included in Tables 5, 6, and 7.

**Table 5:** Adjusted odds ratios of features with 95% confidence intervals (only Spain and UK patients included for PE).

Feature	PE		Death	
	OR (95% CI)	*P* value	OR (95% CI)	*P* value
Age		< 0.005		< 0.005
< 20	1.0	< 0.005	1.0	< 0.005
20–40	5.5 (3.3, 10.1)	< 0.005	2.4 (2.3, 2.6)	< 0.005
40–60	8.0 (4.8, 14.6)	< 0.005	6.8 (6.4, 7.3)	< 0.005
60–80	8.2 (5.0, 15.1)	< 0.005	16.1 (15.1, 17.2)	< 0.005
> 80	5.7 (3.4, 10.5)	< 0.005	27.8 (26.0, 29.7)	< 0.005
Sex (male)	1.3 (1.3, 1.4)	< 0.005	1.2 (1.2, 1.3)	< 0.005
Region				< 0.005
Sub-saharan Africa	–	–	3.3 (3.0, 3.6)	< 0.005
Europe and Central Asia	–	–	0.9 (0.8, 1.0)	0.052
South Asia	–	–	4.0 (3.7, 4.4)	< 0.005
East Asia	–	–	1.0	–
North America	–	–	1.2 (1.1, 1.3)	< 0.005
MENA	–	–	2.8 (2.4, 3.3)	< 0.005
Alpha variant	2.6 (2.4, 2.8)	< 0.005		
Comorbidities				
AIDS/HIV	0.9 (0.5, 1.5)	0.553	1.5 (1.4, 1.5)	< 0.005
Asthma	0.9 (0.9, 1.0)	0.055	0.8 (0.8, 0.9)	< 0.005
Chronic cardiac disease	0.7 (0.6, 0.8)	< 0.005	1.2 (1.2, 1.2)	< 0.005
Chronic haematological	0.9 (0.8, 1.1)	0.320	1.1 (1.0, 1.1)	0.016
Chronic kidney disease	0.9 (0.8, 1.0)	< 0.005	1.4 (1.3, 1.4)	< 0.005
Chronic neurological	0.9 (0.8, 1.0)	0.087	1.1 (1.1, 1.1)	< 0.005
Chronic pulmonary	0.9 (0.8, 0.9)	< 0.005	1.2 (1.2, 1.2)	< 0.005
Dementia	0.7 (0.6, 0.8)	< 0.005	1.3 (1.2, 1.3)	< 0.005

**Table 6:** Adjusted odds ratios of features with 95% confidence intervals (only Spain and UK patients included for PE).

Feature	PE		Death	
	OR (95% CI)	*P* Value	OR (95% CI)	*P* Value
Diabetes	0.8 (0.8, 0.9)	< 0.005	1.3 (1.3, 1.3)	< 0.005
Hypertension	1.3 (1.2, 1.4)	< 0.005	1.0 (1.0, 1.0)	< 0.005
Liver disease	0.9 (0.7, 1.1)	0.200	1.3 (1.2, 1.4)	< 0.005
Malignant neoplasm	1.3 (1.2, 1.4)	< 0.005	1.4 (1.4, 1.5)	< 0.005
Malnutrition	0.8 (0.6, 1.1)	0.200	1.2 (1.1, 1.2)	< 0.005
Obesity	1.4 (1.3, 1.6)	< 0.005	1.2 (1.1, 1.2)	< 0.005
Rheumatologic	1.1 (0.9, 1.2)	0.137	0.9 (0.9, 1.0)	< 0.005
Smoking	1.1 (1.0, 1.2)	< 0.005	1.0 (1.0, 1.0)	0.126
Symptoms				
Symptomatic	1.6 (1.4, 1.9)	<0.005	1.1 (1.1, 1.1)	< 0.005
Abdominal pain	0.8 (0.7, 0.9)	<0.005	0.9 (0.9, 1.0)	< 0.005
Confusion	0.9 (0.8, 1.0)	0.009	1.5 (1.4, 1.5)	< 0.005
Bleeding	1.3 (1.0, 1.6)	0.011	1.1 (1.0, 1.2)	0.025
Chest pain	1.8 (1.6, 1.9)	<0.005	0.7 (0.7, 0.8)	< 0.005
Conjunctivitis	1.6 (0.8, 2.9)	0.260	0.8 (0.6, 1.0)	0.050
Cough	0.9 (0.9, 1.0)	0.391	0.9 (0.9, 1.0)	< 0.005
Diarrhoea	0.9 (0.9, 1.1)	0.568	0.9 (0.9, 0.9)	< 0.005
Ear pain	0.7 (0.3, 1.5)	0.245	1.1 (0.9, 1.4)	0.495
Fatigue	1.1 (1.1, 1.2)	< 0.005	1.0 (0.9, 1.0)	< 0.005
Headache	0.7 (0.7, 0.8)	< 0.005	0.7 (0.6, 0.7)	< 0.005
Fever	0.8 (0.7, 0.9)	< 0.005	1.1 (1.0, 1.1)	< 0.005
Lost sense of smell	1.2 (1.0, 1.4)	0.012	0.7 (0.6, 0.7)	< 0.005
Lost sense of taste	1.0 (0.9, 1.2)	0.490	0.9 (0.8, 0.9)	< 0.005
Lymphadenopathy	1.7 (1.1, 2.6)	< 0.005	1.4 (1.2, 1.7)	< 0.005
Muscle/joint pain	0.9 (0.6, 1.0)	< 0.005	0.8 (0.8, 0.8)	< 0.005
Runny nose	0.9 (0.6, 1.3)	0.832	0.9 (0.8, 1.0)	0.042
Seizures	0.5 (0.3, 0.8)	< 0.005	1.0 (0.9, 1.1)	0.784
Severe dehydration	1.2 (1.0, 1.4)	< 0.005	1.3 (1.2, 1.3)	<0.005

**Table 7:** Adjusted odds ratios of features with 95% confidence intervals (only Spain and UK patients included for PE).

Feature	PE		Death	
	OR (95% CI)	*P* Value	OR (95% CI)	*P* Value
Shortness of breath	2.4 (2.2, 2.6)	< 0.005	1.8 (1.7, 1.8)	< 0.005
Skin rash	1.1 (0.9, 1.4)	0.842	1.3 (1.2, 1.4)	< 0.005
Sore throat	0.7 (0.6, 0.8)	< 0.005	1.0 (0.9, 1.0)	0.181
Vomiting	0.9 (0.8, 1.0)	< 0.005	0.9 (0.8, 0.9)	< 0.005
Wheezing	0.8 (0.7, 1.0)	< 0.005	1.3 (1.2, 1.3)	< 0.005
PE	–	–	1.3 (1.2, 1.4)	< 0.005

**Figure 3:**
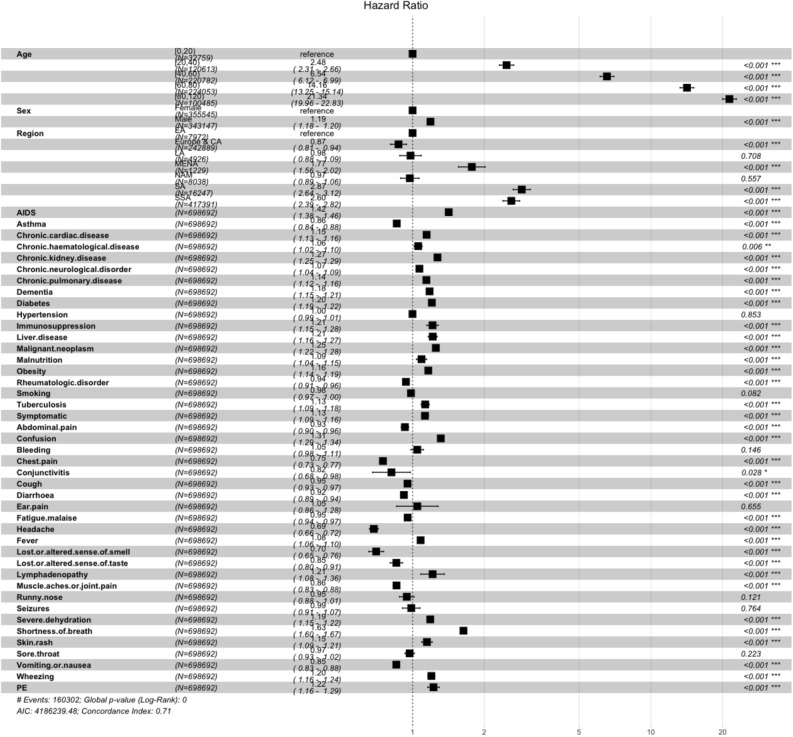
Adjusted hazard ratios for mortality with 95% confidence intervals.

The Cox proportional hazards model without regularisation yielded a C-index of 0.71 and the forest plot shows high hazard ratios for age, certain regions of admission, and specific symptoms (Fig. 3 and Tables 8 and 9).

**Table 8:** Adjusted hazard ratios for mortality.

Feature	HR	95% CI	*P* value
Age			
20–40	2.5	2.3, 2.7	< 0.005
40–60	6.5	6.1, 7.0	< 0.005
60–80	14.2	13.3, 15.1	< 0.005
> 80	21.3	20.0, 22.8	< 0.005
Sex (male)	1.2	1.2, 1.2	< 0.005
Region			
Sub-saharan Africa	2.6	2.4, 2.8	< 0.005
Europe and Central Asia	0.9	0.8, 0.9	< 0.005
South Asia	2.9	2.6, 3.1	< 0.005
North America	1.0	0.9, 1.1	0.557
MENA	1.8	1.6, 2.0	< 0.005
Comorbidities			
AIDS/HIV	1.4	1.4, 1.5	< 0.005
Asthma	0.9	0.8, 0.9	< 0.005
Chronic cardiac disease	1.2	1.1, 1.2	< 0.005
Chronic haematological	1.1	1.0, 1.1	0.006
Chronic kidney disease	1.3	1.3, 1.3	< 0.005
Chronic neurological	1.1	1.0, 1.1	< 0.005
Chronic pulmonary	1.1	1.1, 1.2	< 0.005
Dementia	1.2	1.2, 1.2	< 0.005
Diabetes	1.2	1.2, 1.2	< 0.005
Hypertension	1.0	1.0, 1.0	0.853
Immunosuppression	1.2	1.2, 1.3	< 0.005
Liver disease	1.2	1.2, 1.3	< 0.005
Malignant neoplasm	1.3	1.2, 1.3	< 0.005
Malnutrition	1.1	1.0, 1.2	< 0.005
Obesity	1.2	1.1, 1.2	< 0.005
Rheumatologic	0.9	0.9, 1.0	< 0.005
Smoking	1.0	1.0, 1.0	0.082
Tuberculosis	1.1	1.1, 1.2	< 0.005

**Table 9:** Adjusted hazard ratios for mortality (continued).

Feature	HR	95% CI	*P* value
Symptoms			
Symptomatic	1.1	1.1, 1.2	<0.005
Abdominal pain	0.9	0.9, 1.0	<0.005
Confusion	1.3	1.3, 1.3	<0.005
Bleeding	1.1	1.0, 1.1	0.146
Chest pain	0.8	0.7, 0.8	<0.005
Conjunctivitis	0.8	0.7, 1.0	0.028
Cough	1.0	0.9, 1.0	<0.005
Diarrhoea	0.9	0.9, 0.9	<0.005
Ear pain	1.1	0.9, 1.3	0.655
Fatigue	1.0	0.9, 1.0	<0.005
Headache	0.7	0.7, 0.7	<0.005
Fever	1.1	1.1, 1.1	<0.005
Lost sense of smell	0.7	0.7, 0.8	<0.005
Lost sense of taste	0.9	0.8, 0.9	<0.005
Lymphadenopathy	1.2	1.1, 1.4	<0.005
Muscle/joint pain	0.9	0.8, 0.9	<0.005
Runny nose	1.0	0.9, 1.0	0.121
Seizures	1.0	0.9, 1.1	0.764
Severe dehydration	1.2	1.2, 1.2	<0.005
Shortness of breath	1.6	1.6, 1.7	<0.005
Skin rash	1.2	1.1, 1.2	<0.005
Sore throat	1.0	0.9, 1.0	0.223
Vomiting	0.9	0.8, 0.9	<0.005
Wheezing	1.2	1.2, 1.2	<0.005
PE	1.2	1.2, 1.3	<0.005

**Figure 4:**
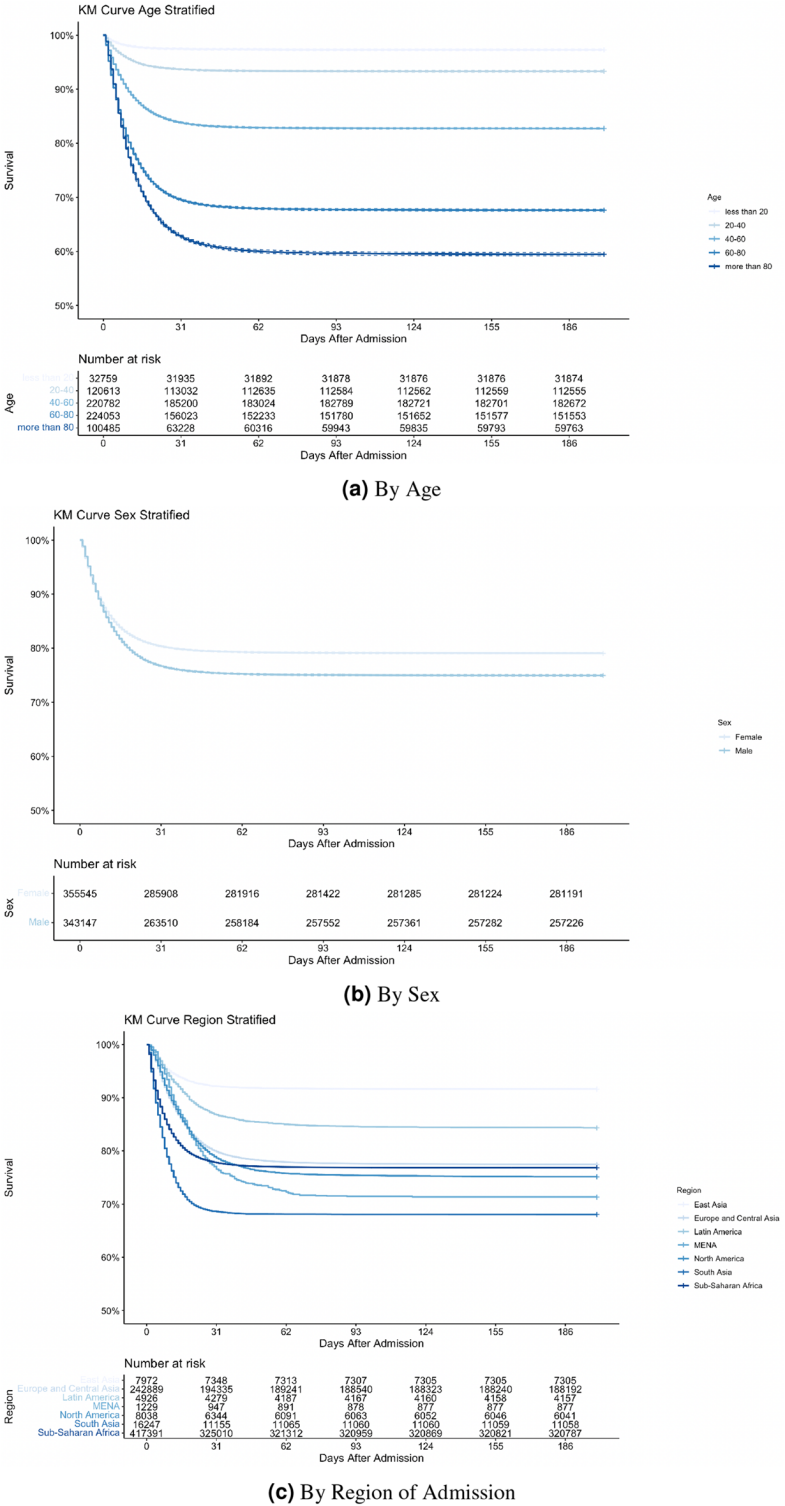
Kaplan–Meier survival curve for COVID-19 patients stratified by **a**) age, **b**) sex, and **c**) region.

The Kaplan-Meier curves for risk stratification across age, sex, and region groups show clear difference in risk with older men and those in South Asia and the Middle East with the lowest rates of survival (Fig. 4).

Tables 10, 11, and 12 show superior performance of the XGBoost model across all 3 test sets. Similarly, XGBoost maintains sensitive and accurate prediction of death compared to other alternative models (Table 13). The validation scores are for the combined UK and Spain set.

**Table 10:** Prediction model results for PE on test set with UK and Spain (F1-w is the weighted F1 score).

Models	Validation AUC	AUC	Accuracy	F1-w	Sensitivity
No undersampling					
No threshold					
Logistic regression	72.5	71.0	64.0	76.5	69.3
LDA	72.2	70.6	98.3	97.4	0.0
Naive Bayes	70.4	69.1	98.1	97.4	0.9
Random forest	73.6	73.5	65.4	77.5	69.7
Stacking Ensemble	63.0	67.3	65.5	77.6	69.1
Ensemble	73.0	71.8	63.7	76.2	70.8
Ensemble (XGBoost)	73.6	73.8	64.5	76.9	70.0
**XGBoost**	**75.6**	**75.9**	**72.3**	**82.3**	**67.5**
No undersampling					
With threshold					
Logistic Regression	72.5	66.7	63.0	75.7	70.5
LDA	72.2	66.5	66.7	78.5	66.3
Naive Bayes	70.4	65.3	61.8	74.9	68.9
Random forest	73.6	66.6	71.4	81.8	61.5
XGBoost	73.8	67.3	67.9	79.3	66.7
With undersampling					
No threshold					
Logistic Regression	72.4	71.0	63.9	76.5	69.1
LDA	72.2	70.6	95.9	96.4	9.8
Naive Bayes	70.4	69.0	82.7	89.0	34.2
Random forest	74.3	73.9	68.3	79.6	66.6
Stacking Ensemble	64.5	67.6	66.8	78.6	68.4
XGBoost	73.8	73.7	66.1	78.1	69.0
With undersampling					
With threshold					
Logistic Regression	72.4	66.3	64.7	77.1	67.9
LDA	72.2	66.7	63.2	75.9	70.3
Naive Bayes	70.4	65.2	61.5	74.6	68.9
Random forest	74.3	73.9	68.3	79.6	66.6
XGBoost	73.8	67.3	62.4	75.3	72.5

Best performing model performance values are in bold.

**Table 11:** Prediction model results for PE on UK test set (F1-w is the weighted F1 score).

Models	Validation AUC	AUC	Accuracy	F1-w	Sensitivity
No undersampling					
No threshold					
Logistic Regression	72.5	69.4	64.9	77.3	65.5
LDA	72.2	69.2	98.4	97.6	0.0
Naive Bayes	70.4	67.2	98.3	97.6	0.5
Random forest	73.6	71.2	65.6	77.8	66.0
Stacking ensemble	63.0	65.7	66.1	78.2	65.2
Ensemble	73.0	70.3	64.7	77.1	67.0
Ensemble (XGBoost)	73.6	71.6	64.9	77.3	66.1
**XGBoost**	**75.6**	**74.5**	**73.4**	**83.2**	**63.5**
No undersampling					
With threshold					
Logistic regression	72.5	65.3	63.9	76.5	66.8
LDA	72.2	65.3	68.7	80.0	61.9
Naive Bayes	70.4	63.6	61.9	75.0	65.5
Random forest	73.6	64.5	71.9	82.2	56.9
XGBoost	73.8	65.5	68.5	79.9	62.5
With undersampling					
No threshold					
Logistic Regression	72.4	69.3	64.8	77.2	65.2
LDA	72.2	69.2	97.0	97.0	4.8
Naive Bayes	70.4	67.2	83.3	89.5	29.7
Random forest	74.3	71.7	68.8	80.1	62.4
Stacking Ensemble	64.5	65.9	67.5	79.2	64.3
XGBoost	73.8	71.7	66.8	78.6	65.1
With undersampling					
With threshold					
Logistic Regression	72.4	64.8	65.6	77.8	64.0
LDA	72.2	65.5	64.5	77.0	66.6
Naive Bayes	70.4	63.5	61.6	74.8	65.5
Random forest	74.3	65.5	69.3	80.5	61.6
XGBoost	73.8	65.9	62.8	75.7	69.1

Best performing model performance values are in bold.

**Table 12:** Prediction model results for PE on Spain test set (F1-w is the weighted F1 score).

Models	Validation AUC	AUC	Accuracy	F1-w	Sensitivity
No undersampling					
No threshold					
Logistic Regression	72.5	74.5	48.5	61.6	96.5
LDA	72.2	73.6	96.2	94.4	0.0
Naive Bayes	70.4	77.6	95.7	94.3	3.5
Random forest	73.6	78.7	62.1	73.3	95.7
Stacking ensemble	63.0	75.7	56.4	68.6	96.5
Ensemble	73.0	74.4	46.5	60.0	95.4
Ensemble (XGBoost)	73.6	79.8	57.8	69.8	95.4
**XGBoost**	**75.6**	**78.9**	**59.6**	**67.2**	**95.7**
No undersampling					
With threshold					
Logistic regression	72.5	71.3	48.1	61.2	96.5
LDA	72.2	64.2	33.5	45.8	97.4
Naive Bayes	70.4	76.3	60.9	72.4	93.0
Random forest	73.6	78.4	64.1	74.9	93.9
XGBoost	73.8	76.0	57.1	69.2	96.5
With undersampling					
No threshold					
Logistic regression	72.4	74.6	49.5	62.6	96.5
LDA	72.2	73.9	78.7	85.0	44.3
Naive Bayes	70.4	77.6	71.8	80.5	66.1
Random forest	74.3	80.2	58.7	70.6	96.5
Stacking ensemble	64.5	75.0	55.1	67.5	96.5
XGBoost	73.8	79.3	54.9	67.4	96.5
With undersampling					
With threshold					
Logistic Regression	72.4	71.8	49.7	62.7	95.7
LDA	72.2	68.2	42.0	55.2	96.5
Naive Bayes	70.4	76.4	61.1	72.5	93.0
Random forest	74.3	76.9	58.7	70.6	96.5
XGBoost	73.8	74.7	54.4	66.9	96.5

Best performing model performance values are in bold.

**Table 13:** Death prediction model results for test set (F1-w is the weighted F1 score).

Models	Validation AUC	AUC	Accuracy	F1-w	Sensitivity
No undersampling					
No threshold					
Logistic regression	73.2	72.9	66.2	68.9	68.4
LDA	73.1	72.9	78.0	71.1	7.8
Naive Bayes	71.3	71.1	74.9	72.5	23.7
Random forest	74.1	73.9	65.5	68.4	71.5
Stacking ensemble	74.1	73.9	65.5	68.4	71.5
Ensemble	73.3	73.1	65.8	68.6	69.4
Ensemble (XGBoost)	74.4	74.3	65.1	68.1	73.0
**XGBoost**	**74.4**	**74.2**	**65.3**	**68.2**	**72.7**
No undersampling					
With threshold					
Logistic regression	73.2	67.0	63.7	66.8	72.9
LDA	73.1	67.1	63.8	66.9	72.8
Naive Bayes	71.3	66.9	62.6	65.8	74.7
Random forest	74.1	67.6	65.9	68.8	70.6
Ensemble	73.3	67.1	62.8	66.0	74.8
Ensemble (XGBoost)	74.4	67.9	66.3	69.1	70.7
XGBoost	74.4	67.9	66.1	69.0	71.1

Best performing model performance values are in bold.

The model also maintains high predictive performance across various subgroups of the patient population stratified across sex and age (Tables 14 and 15).

To further evaluate our model, we test it on held-out test data with specific patient population subgroups including men, women, and different age groups as can be seen in Tables 14 and 15. Our model shows reliable prediction for PE and mortality in both men and women without a significant difference in performance for each group, whereas for age groups there is greater variation in results as compared to sex differences but it remains relatively consistent in predictive performance.

**Table 14:** Prediction model results stratified across sex and age groups for PE (F1-w is the weighted F1 score).

Models	AUC	Accuracy	F1-w	Sensitivity
Sex				
Male				
Logistic regression	71.1	55.9	69.9	77.7
XGBoost	76.0	68.0	79.1	73.0
Female				
Logistic regression	69.3	73.7	83.7	53.4
XGBoost	74.3	77.3	86.0	57.2
Age				
20–40				
Logistic regression	79.0	65.8	78.0	81.0
XGBoost	78.2	74.1	83.5	68.8
40–60				
Logistic Regression	65.7	48.7	63.2	75.9
XGBoost	74.0	59.0	71.9	78.2
60–80				
Logistic regression	69.6	58.6	72.0	71.5
XGBoost	72.3	66.1	77.6	67.7

**Table 15:** Prediction model results stratified across sex and age groups for death (F1-w is the weighted F1 score).

Models	AUC	Accuracy	F1-w	Sensitivity
Sex				
Male				
Logistic regression	72.4	64.4	67.0	71.2
XGBoost	74.0	64.2	66.8	74.6
Female				
Logistic regression	72.9	67.9	70.8	65.1
XGBoost	74.0	66.3	69.6	70.4
Age				
20–40				
Logistic regression	67.8	93.4	90.2	0.0
XGBoost	70.2	93.1	90.5	4.2
40–60				
Logistic regression	62.9	77.8	76.2	18.1
XGBoost	65.0	74.3	75.2	32.1
60–80				
Logistic regression	61.7	47.6	46.4	85.8
XGBoost	63.6	46.9	44.9	89.2

**Figure 5:**
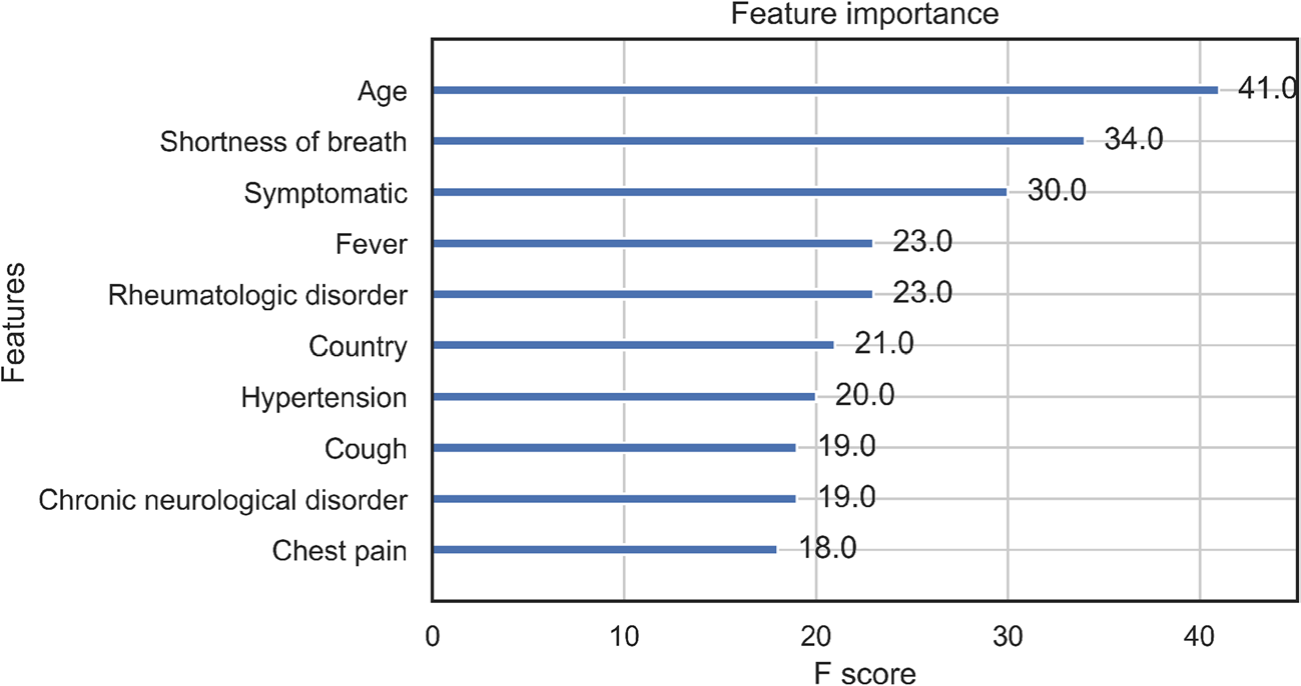
Feature importance from XGBoost PE prediction model using F1-score gain method (average contribution of each feature to predictive performance).

**Figure 6:**
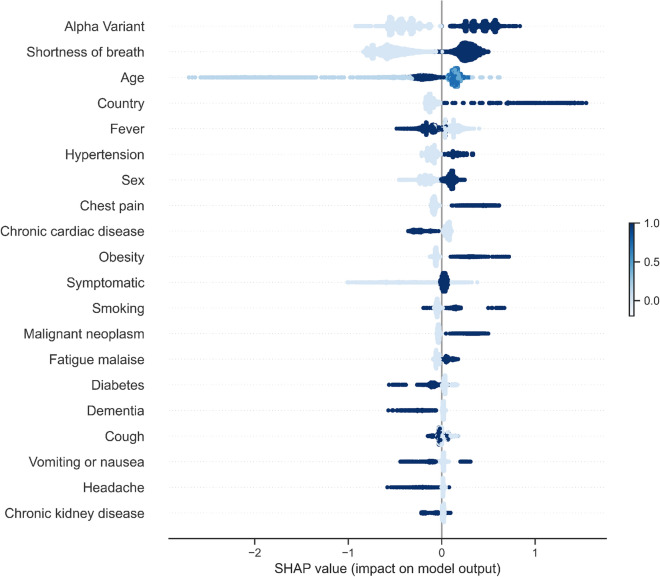
XGBoost feature importance with SHAP for PE. The values in the legend being higher or darker colour in the plot correspond to higher values of that feature contributing to the prediction either for stronger positive prediction (more colour points for the feature on the right side of the vertical line) or stronger negative prediction of outcome otherwise.

**Figure 7:**
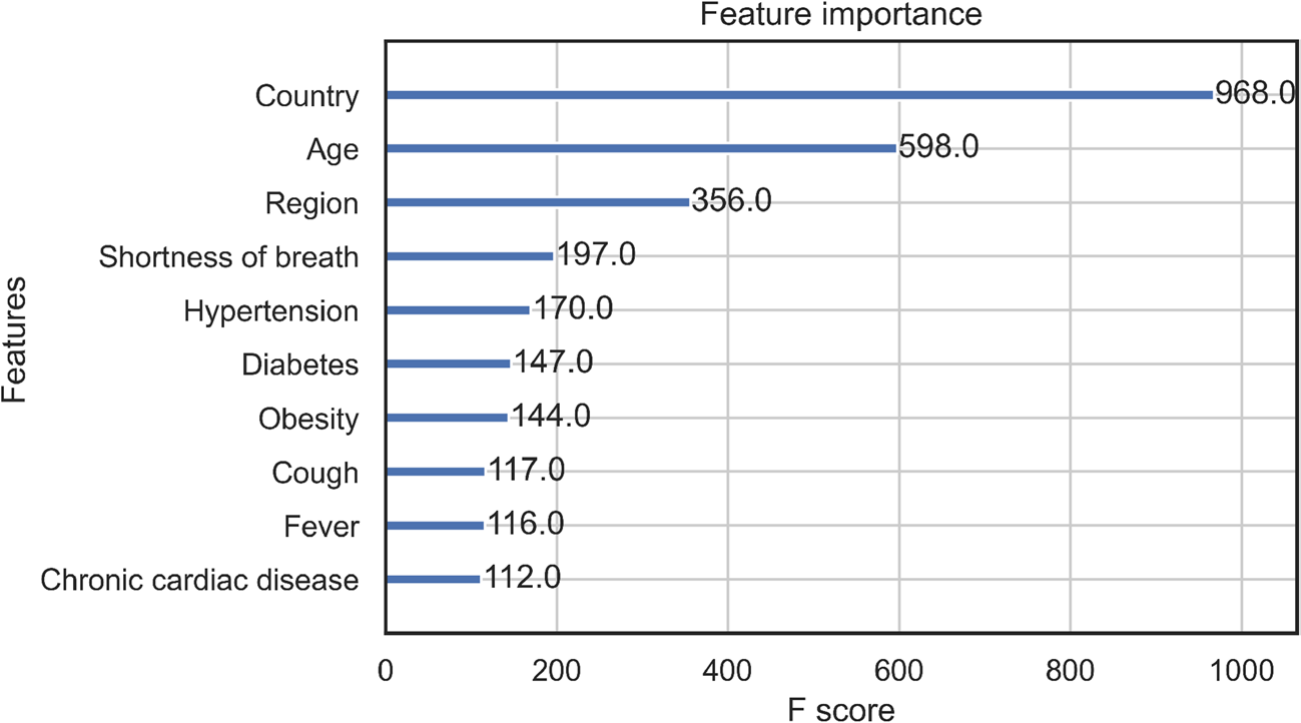
Feature importance from XGBoost mortality prediction model using F1-score gain method (average contribution of each feature to predictive performance).

**Figure 8:**
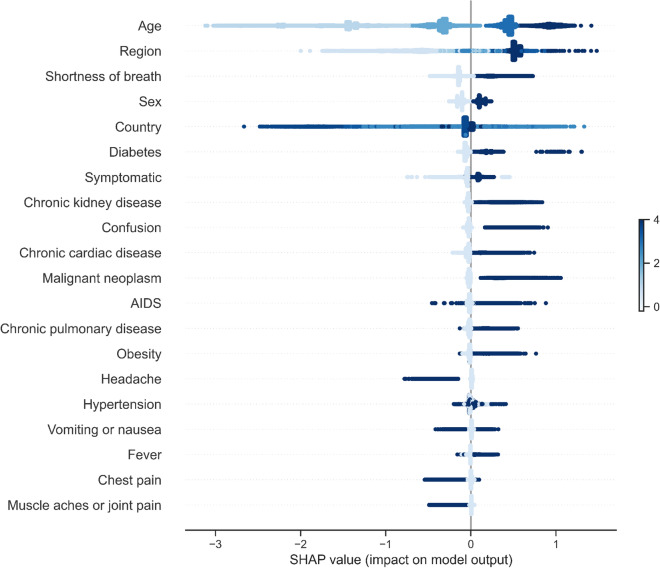
XGBoost feature importance with SHAP for mortality. The values in the legend being higher or darker colour in the plot correspond to higher values of that feature contributing to the prediction either for stronger positive prediction (more colour points for the feature on the right side of the vertical line) or stronger negative prediction of outcome otherwise.

**Figure 9:**
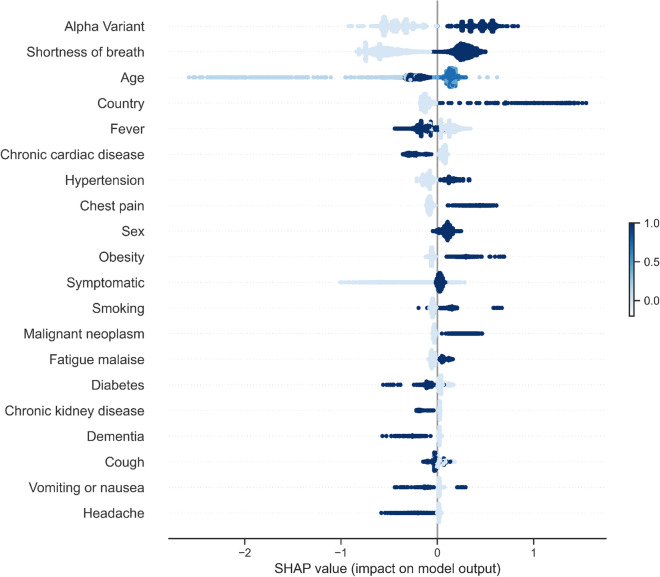
XGBoost feature importance with SHAP for PE (only men). The values in the legend being higher or darker colour in the plot correspond to higher values of that feature contributing to the prediction either for stronger positive prediction (more colour points for the feature on the right side of the vertical line) or stronger negative prediction of outcome otherwise.

**Figure 10:**
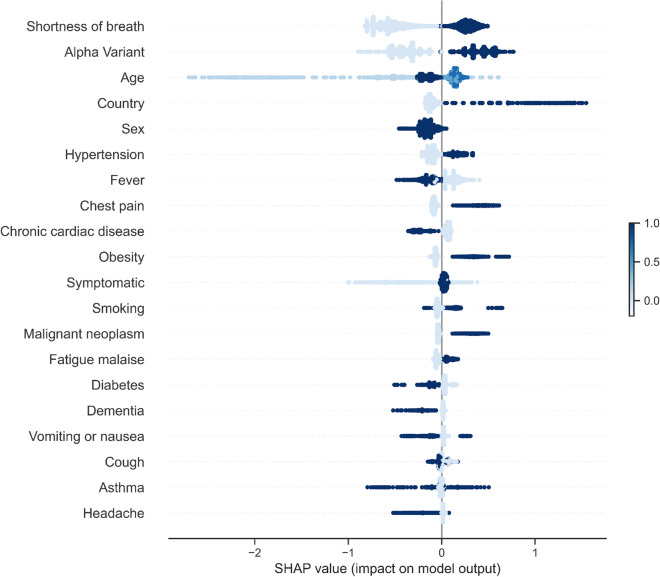
XGBoost feature importance with SHAP for PE (only women). The values in the legend being higher or darker colour in the plot correspond to higher values of that feature contributing to the prediction either for stronger positive prediction (more colour points for the feature on the right side of the vertical line) or stronger negative prediction of outcome otherwise.

**Figure 11:**
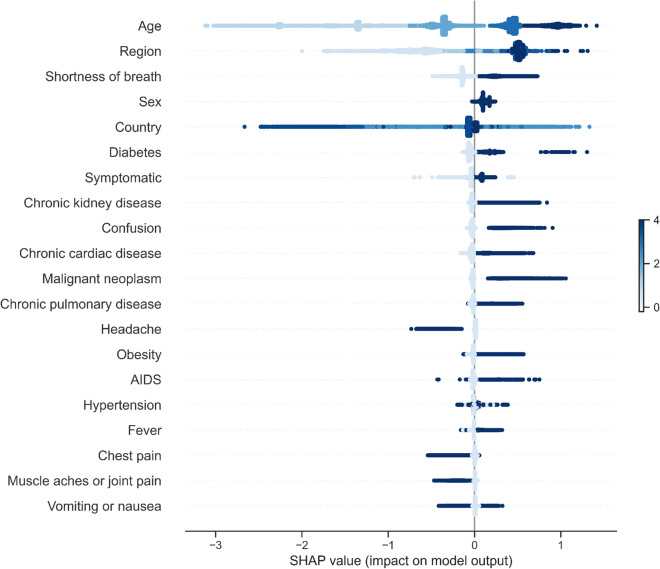
XGBoost feature importance with SHAP for mortality (only men). The values in the legend being higher or darker colour in the plot correspond to higher values of that feature contributing to the prediction either for stronger positive prediction (more colour points for the feature on the right side of the vertical line) or stronger negative prediction of outcome otherwise.

**Figure 12:**
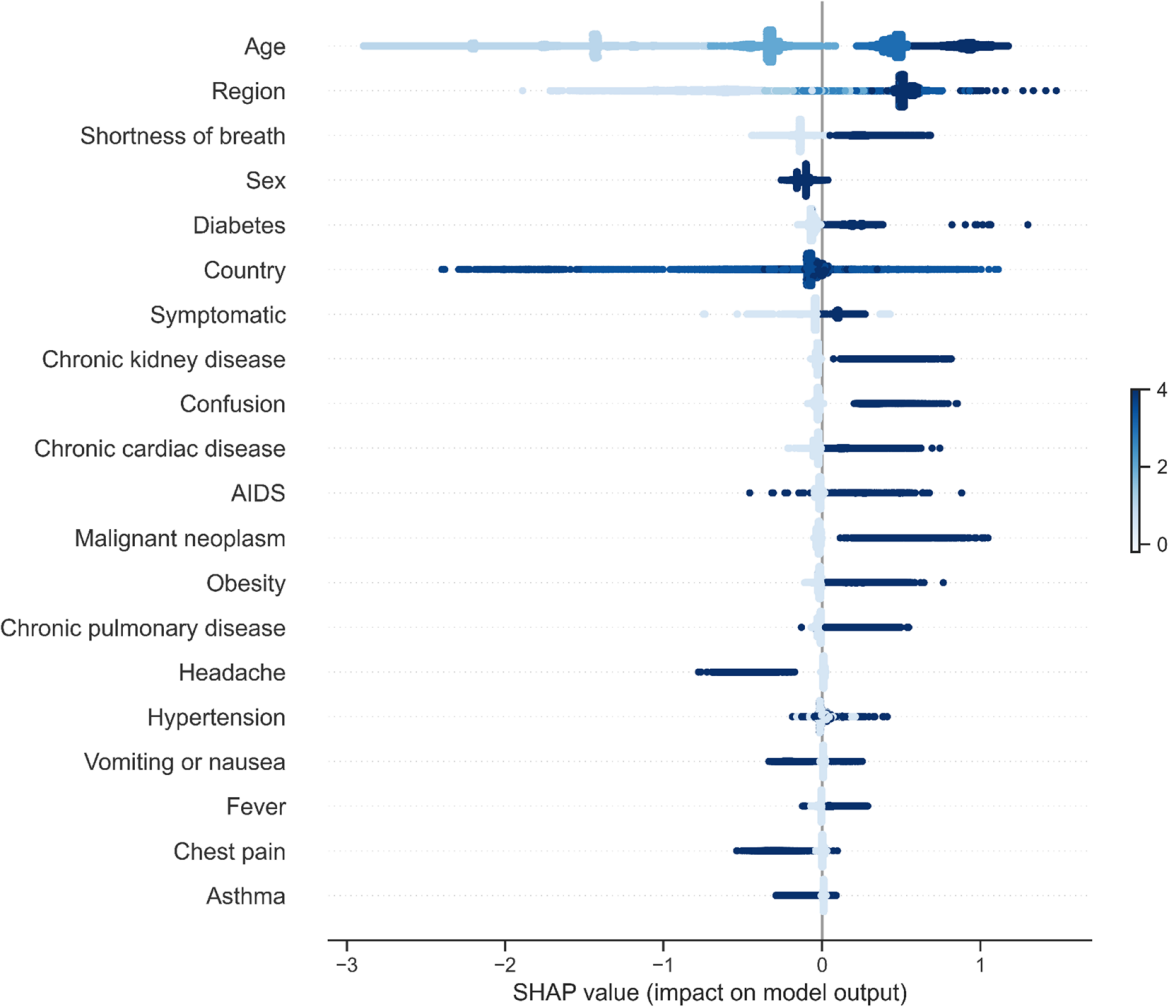
XGBoost feature importance with SHAP for mortality (only women). The values in the legend being higher or darker colour in the plot correspond to higher values of that feature contributing to the prediction either for stronger positive prediction (more colour points for the feature on the right side of the vertical line) or stronger negative prediction of outcome otherwise.

Taking the best performing XGBoost model and applying 2 different feature importance methods, average f1-score gain across splits and Shapley values, we obtain the results seen in Figs. 5, 6, 7, and 8. A feature importance stratification on a held-out test set of only men and only women separately for either PE or mortality prediction is also included in Figs. 9, 10, 11, and 12. As further clarification for the SHAP plot, darker colour indicates that a higher value of that feature contributes to the prediction either positively (if on the right hand side of the vertical line) or negatively (if on the left hand side of the vertical line). Higher placement of the feature vertically in the plot means it has a higher mean Shapley value and hence contributes more to correct predictions in the model.

## Discussion

To our knowledge, this multi-center dataset is the largest international cohort of hospitalised COVID-19 patients available. Our analysis showed that patients with PE are older, more often male, white, from higher income countries, and are more likely to suffer from: asthma, chronic cardiac disease, chronic kidney disease, chronic neurological disease, chronic pulmonary disease, hypertension, cancer, obesity, rheumatologic conditions, or smoke.

The occurrence of pulmonary embolism in our study population was 0.7% and our results showed a significant association between confirmed PE and mortality when compared with patients without PE as has been similarly found in patients without COVID-19^17^.

Accordingly, our logistic regression models for PE and death showed that different age-groups experience different risks of either outcome. The age group 40-80 was at highest odds of having PE, and those >60 of dying as can be seen in the Kaplan-Meier curve in Fig. 4a. Symptomatic COVID-19 patients were almost 3 times more likely to experience PE while also being more likely to die. Within symptoms and comorbidities, shortness of breath, chest pain, obesity, and bleeding were associated with higher odds of a PE, followed by hypertension and loss of smell. The regionality of the data must be addressed in the higher odds of death in South Asia, Middle East and North Africa (MENA), and South Africa compared to Europe and Central Asia as the hospital centers in those communities have different challenges and circumstances when it comes to fighting the pandemic. Symptoms like shortness of breath, confusion, severe dehydration, and wheezing were present in COVID-19 patients with higher odds of death, and comorbidities such as malignant neoplasm, diabetes, and chronic kidney or liver disease also lead to higher risk of death. For both correlation and odds of PE and death, men were more at risk. This is shown in the Kaplan–Meier curves for survival stratified across sex in Fig. 4b.

The hazard ratios confirmed those over the age of 60 were at highest risk of death, especially those COVID-19 patients who experienced shortness of breath, severe dehydration, confusion, and had pre-existing chronic conditions. Regionality of hospital admission was once again an important risk factor for death. Interestingly, patients with PE, chest pain, asthma, or fever seemed to have lower risk associated which could be due to earlier and easier detection of these symptoms and conditions in the progression of the disease.

Seeking to combine this clinically insightful information for outcome prediction, we developed a fast prediction model with XGBoost for both PE and death in COVID-19 hospitalised patients, and tested it in different countries separately. We also showed that appropriate class weighting can help with class imbalance and even outperform ensemble resampling methods without having to sacrifice the interpretability of the model (Tables 10, 13). The differences between measured performance on UK and Spain test sets as evaluated by sensitivity and accuracy are due to different class-imbalance ratios and positive case distributions between the datasets. It is important to note that the metric to focus on for our purposes are the validation and test AUC which remain consistent between the two datasets at around 75% as it is the most robust metric in the face of extreme class-imbalance. Since the class-imbalance varies between the two datasets as well, other metrics like sensitivity and accuracy will be significantly impacted despite attempts to dampen it but due to only a few percentages of positive cases, the potency of our approaches can only be limited. The best-performing model for PE prediction evaluated across separate held-out UK-only data, Spain-only data, and UK and Spain data combined is XGBoost without undersampling and without rigid thresholding using robust class weighting. As for death, the XGBoost again outperformed all other models including the ensemble with XGBoost on some metrics.

Since our XGBoost model outperformed other methods, we also showed that the best method for handling class imbalance is through robust class weighting and compared it to other methods for imbalance handling like ensembles and resampling methods. Another advantage of this method is that it avoids introducing bias like in the case of resampling. Finally, XGBoost provides feature importances which was useful for explaining clinical risk prediction of the model to healthcare professionals and policy-makers.

Exploring two different interpretability methods for XGBoost, average gain across splits and Shapley values, showed that the time of dominant presence of the alpha variant, age, fever, shortness of breath, and hypertension were the key predictors for PE, followed by region of admission, sex, and chest pain. Recent work has alluded to an association between the alpha variant and occurrence of thromboembolisms in mice but further research relevant to human samples is missing^18^. Age was a complex non-linear predictor with different age groups corresponding to varying risks. The clear colour separation for the Shapley values for age in Fig. 8 showed how each age group has a clearly separable predictive value for mortality with older groups having higher risk but which is not the case for PE as younger age groups can be more predictive of higher PE risk. Furthermore, Shapley values analysis identified obesity, smoking, and the presence of cough as important predictors for PE whereas the default XGBoost method does not. The most predictive features for all-cause mortality were age, region of hospital admission, sex, diabetes, and shortness of breath whereas the default method highlights hypertension and obesity in addition. For mortality, higher values of region corresponded to samples from South Asia and South Africa. Comparing all of the top identified predictors across these models for all outcomes can be seen in Tables 16 and 17 where certain symptoms and comorbidities have been identified to be universally predictive risk factors right at-admission without any additional measurements having to be taken for PE and mortality risk assessment.

**Table 16:** Features of Significant Importance for PE and Mortality Prediction According to Different Models (For XGBoost Top 20 SHAP Value Features Were Taken as Important, and for Logistic Regression and Cox model significance was taken as $p<$0.005).

Feature	PE		Death		
	LR	XGBoost	LR	Cox	XGBoost
Age	$\checkmark$	$\checkmark$	$\checkmark$	$\checkmark$	$\checkmark$
Sex	$\checkmark$	$\checkmark$	$\checkmark$	$\checkmark$	$\checkmark$
Region	$\checkmark$	$\checkmark$	$\checkmark$	$\checkmark$	$\checkmark$
Alpha variant	$\checkmark$	$\checkmark$			
Comorbidities					
AIDS/HIV	X	X	$\checkmark$	$\checkmark$	$\checkmark$
Asthma	X	X	$\checkmark$	$\checkmark$	X
**Chronic Cardiac Disease**	$\checkmark$	$\checkmark$	$\checkmark$	$\checkmark$	$\checkmark$
Chronic Haematological	X	X	$\checkmark$	X	X
**Chronic Kidney Disease**	$\checkmark$	$\checkmark$	$\checkmark$	$\checkmark$	$\checkmark$
Chronic Neurological	X	X	$\checkmark$	$\checkmark$	X
**Chronic Pulmonary**	$\checkmark$	$\checkmark$	$\checkmark$	$\checkmark$	$\checkmark$
**Dementia**	$\checkmark$	$\checkmark$	$\checkmark$	$\checkmark$	X
**Diabetes**	$\checkmark$	$\checkmark$	$\checkmark$	$\checkmark$	$\checkmark$
**Hypertension**	$\checkmark$	$\checkmark$	$\checkmark$	X	$\checkmark$
Liver Disease	X	X	$\checkmark$	$\checkmark$	X
**Malignant Neoplasm**	$\checkmark$	$\checkmark$	$\checkmark$	$\checkmark$	$\checkmark$
Malnutrition	$\checkmark$	X	$\checkmark$	$\checkmark$	X
**Obesity**	$\checkmark$	$\checkmark$	$\checkmark$	$\checkmark$	$\checkmark$
Rheumatologic	X	X	$\checkmark$	$\checkmark$	X
**Smoking**	$\checkmark$	$\checkmark$	X	X	X

Ticks correspond to significance of feature for that model and for that outcome and X corresponds to lack of significance. Features in bold are those found to be significant for both mortality and PE prediction.

**Table 17:** Features of Significant Importance for PE and Mortality Prediction According to Different Models (For XGBoost Top 20 SHAP Value Features Were Taken as Important, and for Logistic Regression and Cox model significance was taken as *p* < 0.005).

	PE		Death		
Feature	LR	XGBoost	LR	Cox	XGBoost
*Symptoms*					
**Symptomatic**	$\checkmark$	$\checkmark$	$\checkmark$	$\checkmark$	$\checkmark$
Abdominal Pain	$\checkmark$	X	$\checkmark$	$\checkmark$	X
Confusion	$\checkmark$	X	$\checkmark$	$\checkmark$	$\checkmark$
Bleeding	$\checkmark$	X	$\checkmark$	$\checkmark$	X
**Chest Pain**	$\checkmark$	$\checkmark$	$\checkmark$	$\checkmark$	$\checkmark$
Conjunctivitis	X	X	$\checkmark$	$\checkmark$	X
Cough	X	$\checkmark$	$\checkmark$	$\checkmark$	X
Diarrhoea	X	X	$\checkmark$	$\checkmark$	X
Ear Pain	X	X	X	X	X
**Fatigue**	$\checkmark$	$\checkmark$	$\checkmark$	$\checkmark$	X
**Headache**	$\checkmark$	$\checkmark$	$\checkmark$	$\checkmark$	$\checkmark$
**Fever**	$\checkmark$	$\checkmark$	$\checkmark$	$\checkmark$	$\checkmark$
Lost Sense of Smell	$\checkmark$	X	$\checkmark$	$\checkmark$	X
Lost Sense of Taste	X	X	$\checkmark$	$\checkmark$	X
Lymphadenopathy	X	X	$\checkmark$	$\checkmark$	X
**Muscle/Joint Pain**	$\checkmark$	X	$\checkmark$	$\checkmark$	$\checkmark$
Runny Nose	X	X	$\checkmark$	X	X
Seizures	$\checkmark$	X	X	X	X
Severe Dehydration	$\checkmark$	X	$\checkmark$	$\checkmark$	X
**Shortness of Breath**	$\checkmark$	$\checkmark$	$\checkmark$	$\checkmark$	$\checkmark$
Skin Rash	X	X	$\checkmark$	$\checkmark$	X
Sore Throat	$\checkmark$	X	X	X	X
**Vomiting**	$\checkmark$	$\checkmark$	$\checkmark$	$\checkmark$	$\checkmark$
Wheezing	$\checkmark$	$\checkmark$	$\checkmark$	$\checkmark$	X
PE	–	–	$\checkmark$	$\checkmark$	X

Ticks correspond to significance of feature for that model and for that outcome and X corresponds to lack of significance. Features in bold are those found to be significant for both mortality and PE prediction. (continued).

The pulmonary embolism and mortality prediction model can help with management of COVID-19 as it uses standard demographics, comorbidity, and symptom data collected at admission for identifying patients most at risk of developing PE which may enable an earlier start of targeted anticoagulation therapy. Our mortality risk prediction model can also help with patient population risk assessment and prioritisation across different regions of the world.

A strength of the current study is that a combination of machine learning and traditional statistical modeling can offer a more reliable system for predictive risk forecasting. XGBoost provides at-admission prediction of both events, while odds and hazards ratios obtained from logistic regression and the Cox proportional hazards model give us an insight into stratified risk and global feature importance. We systematically compare our XGBoost model with different risk prediction algorithms. Our model also outperforms recently published results across a variety of metrics like AUROC and sensitivity despite being developed on a much larger and more heterogeneous and diverse dataset while being robust to class imbalance^19^. With existing scores built on non-COVID-19 data like The National Early Warning Score 2, there is insufficient information available on their reliability in the COVID-19 setting, and some have been found to underestimate mortality^20^. Our model is able to deploy at admission for both PE and death risk prediction and can help supplant these needs rapidly.

The study, however, has several limitations. First, almost 60% of patients who died did so in South Africa, and over 70% of PE cases were located in the UK. This may be due to limited access to d-dimer tests or CT scans. There were no mandatory diagnostic criteria in the ISARIC CRF for PE. The absence of a control group of patients without COVID-19 in this dataset prevented estimation of specificity. The patient cohort comprised of hospitalised patients with confirmed COVID-19 who had a mortality rate of 21.7%. These models are not for use in the community and could still perform differently in populations at lower risk of death and across different regions of the world. As part of future work, dependent on sufficient data, PE and death could be modelled with a comprehensive multi-state statistical framework, which incorporates the interrelations among survival, PE, and death states.

In conclusion, the set of decisions taken must include different stakeholders like patients, clinicians, hospital administrators, researchers, and data procurers so that trade-offs can be identified and context-informed decisions can be taken to address them, especially if our models could have missed harms or benefits to different groups and communities.

## Methods

### Data

In this work, we use data of COVID-19 patients from The International Severe Acute Respiratory and Emerging Infection Consortium (ISARIC), a repository that standardises and secures data on COVID-19 assembled from a global cohort over 2 years of the pandemic as of January 2022. It includes so far data on over 800,000 patients from 53 countries. These data capture the global experience of the first 2 years of the pandemic^21^. The clinical characterisation protocol underwent ethical review by the World Health Organization Ethics Review Committee and ethics approval was obtained for each participating country and site according to local requirements. Ethics Committee approval was given by the WHO Ethics Review Committee (RPC571 and RPC572, 25 April 2013). Institutional approval was additionally obtained by participating sites including the South Central-Oxford C Research Ethics Committee in England (Ref. 13/SC/0149), the Scotland A Research Ethics Committee (Ref. 20/SS/0028) for the UK and the Human Research Ethics Committee (Medical) at the University of the Witwatersrand in South Africa as part of a national surveillance programme (M160667), which collectively represent the majority of the data. Other institutional and national approvals are in place as per local requirements. This is a secondary analysis of data collected, with appropriate local permissions, and each institution signed a Terms of Submission in which committed that they had the appropriate permissions in place. All methods were performed in accordance with the relevant guidelines and regulations.

The study population consisted of all patients with either clinically diagnosed or laboratory confirmed COVID-19 admitted to the participating hospitals. The aim of the recruiting sites was to use a consecutive sample.

The dataset contains 800,459 patients and 182 variables. The mean age of patients was 56.4 (20.9), 48.6% were male, and the majority of cases were from South Africa (54.0%) and the United Kingdom (34.1%). 65.3% of patients were discharged alive and 20.4% died. We grouped countries with fewer than 60 individuals into a single category. Out of all patients, 5450 (0.7%) experienced a pulmonary embolism, 73 experienced thromboembolism, and 143 experienced deep vein thrombosis. We define our outcome of interest as the main pulmonary embolism (PE) diagnosis for subsequent analysis. 4653 (82.1%) of the PE cases were recorded in the United Kingdom (UK) and Spain which based on our knowledge makes our study the largest study of its kind for PE to date. Due to similar data collection patterns and recording, we used data from these two countries only for PE modelling as they contain the vast majority of reported PE cases.

### Data preprocessing

**Figure 13:**
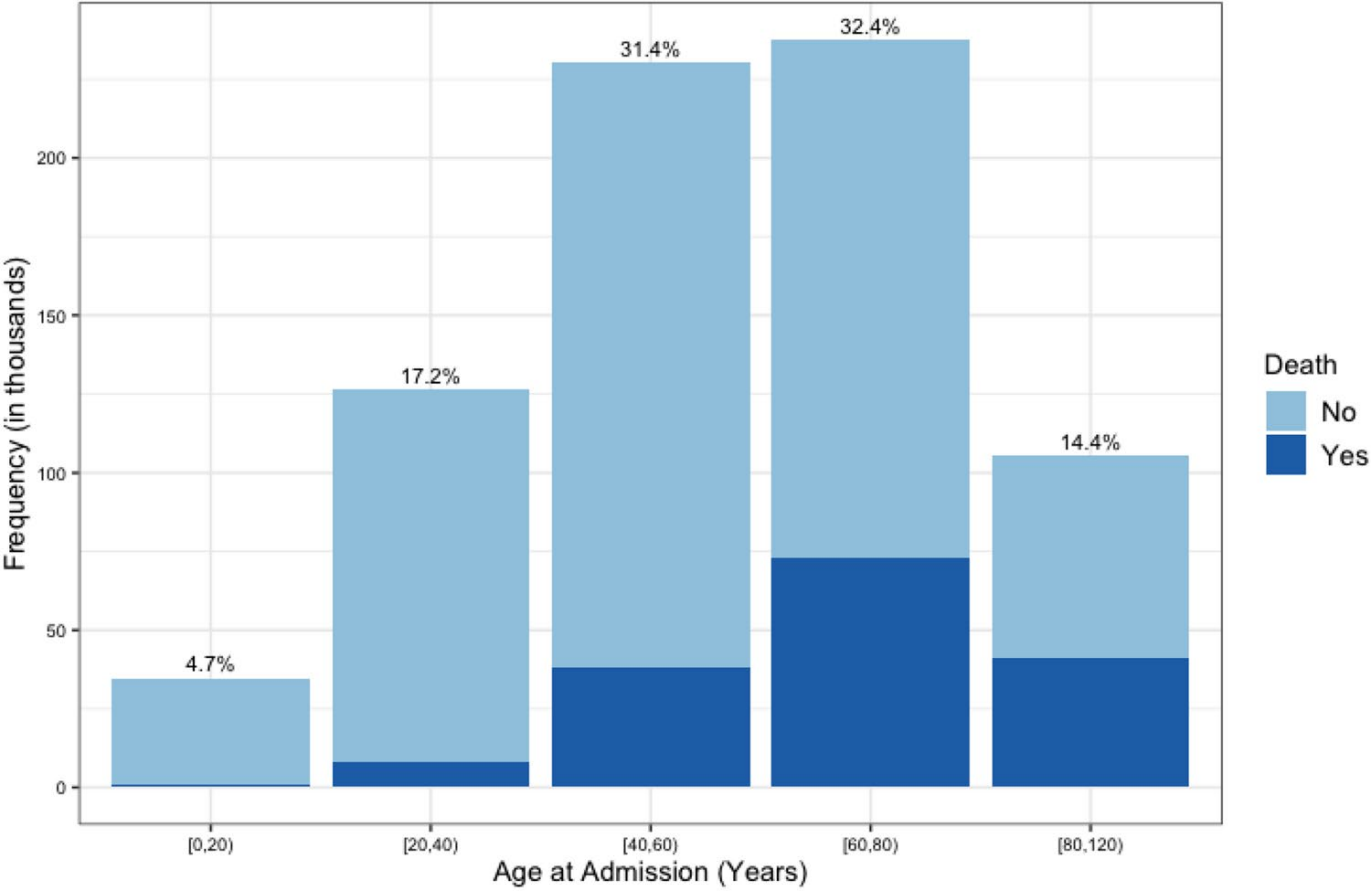
Age distribution for all patients stratified by death outcome.

**Figure 14:**
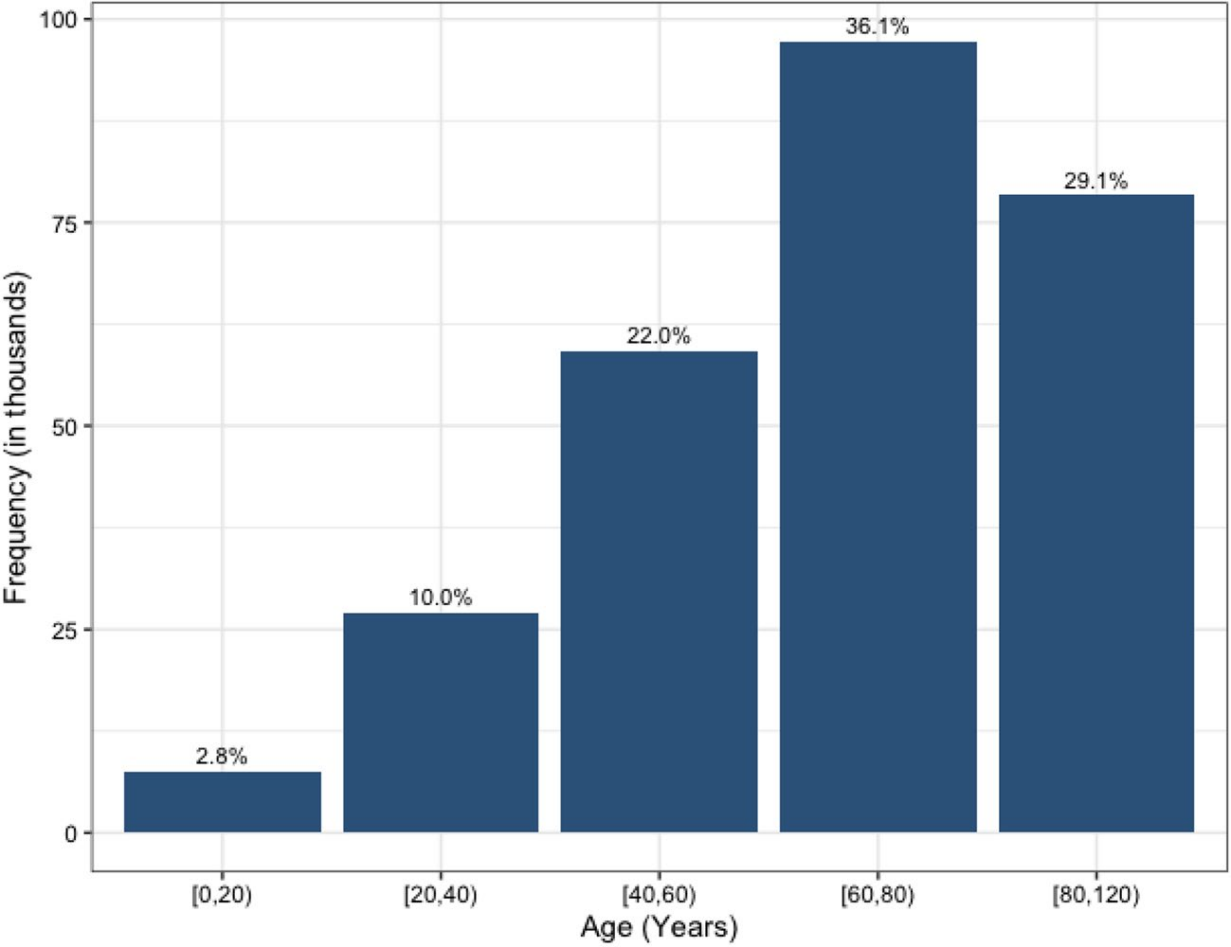
Age distribution for UK and Spain patients.

Since treatment information does not have reliable timestamps for most patients, the following variables were used in the analysis for PE: demographics (including age, sex, country), comorbidities (hypertension, diabetes, smoking etc.), and symptoms (coughing, fever, fatigue etc.). The presence of diagnosis during domination by the alpha variant was also included (after December 2020) due to its possible association with incidence of PE. In our modelling of PE, we used data from patients from the UK and Spain only and did not use laboratory measurements or imputation methods. 269,373 patients and 45 variables remained for PE, and 734,282 patients and 55 variables for death. Age was grouped into 5 categories (0–20, 20–40, 40–60, 60–80, 80–120) with the distributions seen in Figs. 13 and 14 below. The *symptomatic* variable represents any symptoms reported for a patient. For number of days from admission to event (death), we removed outliers of more than 200 days and those in the negatives, thereby removing 1342 patients.

**Figure 15:**
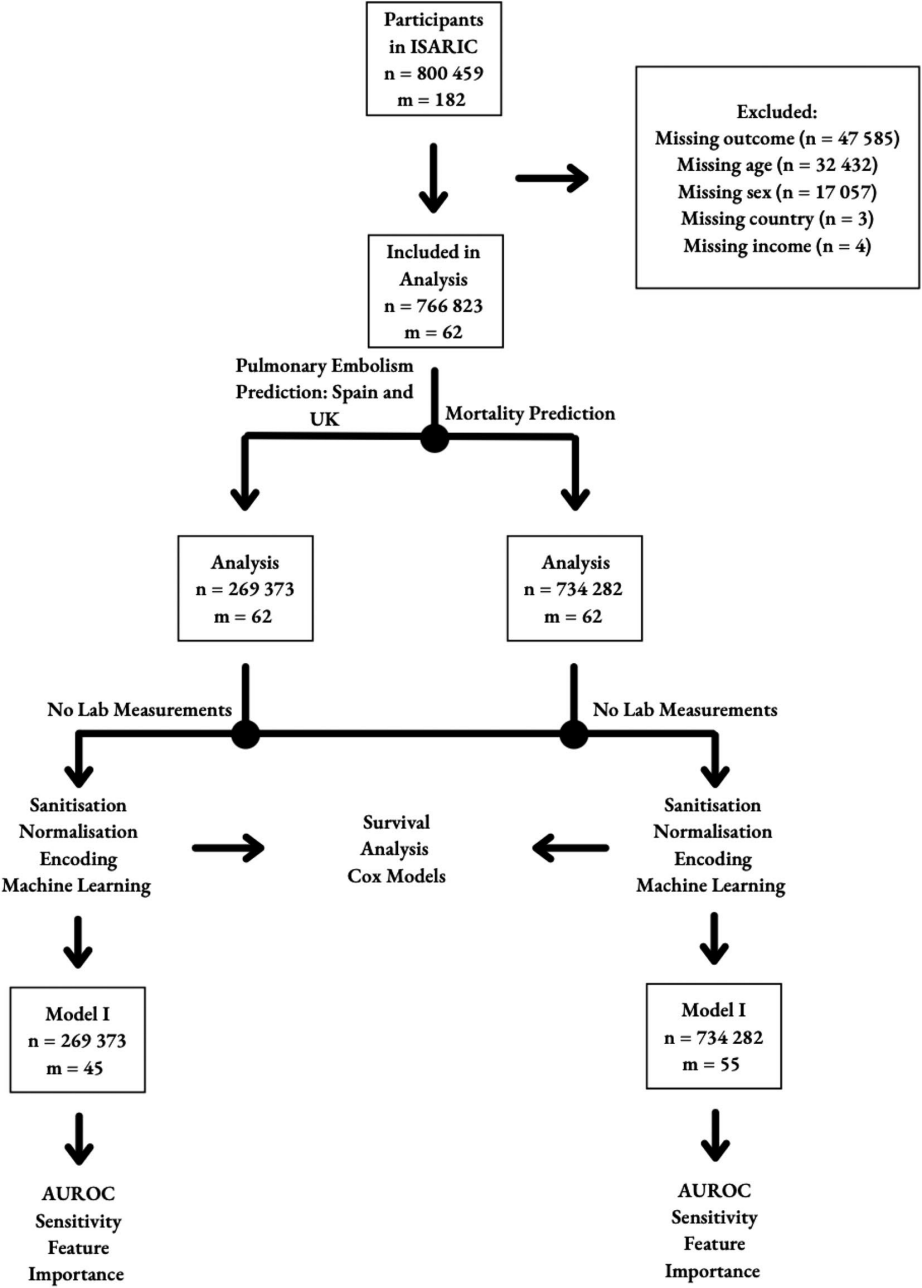
Flowchart of framework with machine learning model to predict the risk of PE and mortality at admission.

Prior to processing the data, for PE prediction, we held out 3 test sets of 20% of the total dataset for independent testing, one of which would only include patients from Spain, one only including patients from the UK, and another including both. For mortality prediction testing, we held out 20% of the total dataset sample. A workflow diagram for data processing and system design is shown in Fig. 15.

Stratified Kaplan–Meier curves by age, sex, and region of admission were plotted using Cox proportional hazards models while machine learning methods were applied for prediction of PE or death.

### Baseline and machine learning methods

The reference groups for statistical analysis for age were those under 20 years old, for region it was East Asia, and for country variable it was Norway. For Cox proportional hazards model, proportionality assumption was verified through visualisation of the survival curves and observing parallel behaviour as seen in Fig. 4. We investigated several prediction methods for PE occurrence and death, including logistic regression, Linear Discriminant Analysis (LDA), naive Bayes classifier, random forests, ADABoosting algorithms, and the high-performing extreme gradient boost machine (XGBoost)^22,23^. Previous studies looking at tree-based algorithms such as XGBoost have highlighted its capacity to learn the correlations between covariates well when it comes to mortality prediction in COVID-19 patients while also being somewhat interpretable^24^. We applied all of these methods for the purposes of a systematic comparison using 5-fold cross-validation, several hold-out test sets stratified across countries and regions, and evaluated with multiple metrics. A list of methods applied can be seen in Table 18 with details in Supplementary.

**Table 18:** Machine learning methods investigated.

Models	Brief description
Logistic regression	Generalised linear model
Linear Discriminant Analysis	Normal distribution, linear
Naive Bayes	Independence, probabilistic
Random Forest	Decision tree ensemble
XGBoost	Gradient-boosted decision trees
Ensemble	AdaBoost models ensemble
Ensemble with XGBoost	XGBoost models ensemble

As is often the case in disease prediction, there is class imbalance with about 1.7% of UK and Spain patients having been diagnosed with PE and around 20.4% having died in the case population. To address this, we use other metrics mentioned above in the evaluation of our models besides accuracy as it does not capture the true predictive performance of our models and we rely more on sensitivity and the F1 score. We also use a different threshold for prediction after probability estimation instead of the default 0.5 to achieve cost-sensitivity, and we apply random undersampling at a minority:majority ratio of 1:4 as has been highlighted in other work^25,26^. We evaluate these methods both separately and in combination to investigate the best approach for this set of prediction problems.

To address imbalance in predictions, we applied either undersampling, thresholding, or both. As for death, due to a much softer imbalance, undersampling was not necessary. We also added class-weighting to our XGBoost model using inverse proportions and compared it with the other methods to address class imbalance. All confusion matrices and parameter details for each model can be found in the Supplementary.

Furthermore, we build an ensemble that combines AdaBoosted decision trees with robust undersampling using different subsets for resampled training so as to address the imbalance and compare this cost-sensitive model with our best performing model and add further confidence in its ability to generalise in an imbalanced scenario^27^. We extend the ensemble learning methods by using our own XGBoost model in the ensemble structure instead of the standard AdaBoosted decision trees. The number of trees was a tunable hyperparameter listed in the Supplementary (Tables IV–VI). We compare our cost-sensitive class-weighted XGBoost machine learning model with these resampling ensembles to show improved performance without the need of introducing bias like in the case of resampling while maintainting interpretability.

### Model validation and evaluation

We proceed to tune our machine learning models and validate them using stratified 5-fold cross-validation with Bayesian optimisation. We repeated the optimisation procedure for 50 iterations after which we evaluated the model on the independent test set with the following metrics: AUROC, Accuracy, Weighted F1 Score, and Sensitivity. The details can be found in the Supplementary.

While existing studies referenced in the Introduction mention some approaches to feature importance estimation for COVID-19 mortality and outcome prediction as well as for other problems, rarely does one find several interpretability methods compared and contrasted in one scenario. We implemented both tree-based F-score interpretability methods as well as Shapley values analysis, logistic regression and Cox regression, and hope to draw interesting conclusions from each and their comparisons^28,29^. A full explanation for the Shapley values method and its details can be found in the Supplementary materials.

## Role of the funding source

The funder had no role in study design, data collection, data analysis, data interpretation, writing of the report, and decision to submit the paper for publication.

### Supplementary Information

Supplementary Information.

## Data Availability

The ISARIC-WHO CCP, case report form and consent forms are openly available on the ISARIC website at https://isaric.org/re search/covid-19-clinical-research-resources/clinical-characterisation-protocol-ccp/. Informed consent for data collection, sharing and/or analysis was obtained from individual participants or their representatives when required by local ethics committees. Some committees approved a waiver of consent due to the public benefit of the research and the minimal risk to participants. The data that underpin this analysis are highly detailed clinical data on individuals hospitalised with COVID-19. Due to the sensitive nature of these data and the associated privacy concerns, they are available via a governed data access mechanism following review of a data access committee. Data can be requested via the IDDO COVID-19 Data Sharing Platform (http://www.iddo.org/covid-19). The Data Access Application, Terms of Access and details of the Data Access Committee are available on the website. Briefly, the requirements for access are a request from a qualified researcher working with a legal entity who have a health and/or research remit; a scientifically valid reason for data access which adheres to appropriate ethical principles. The full terms are at https://www.iddo.org/document/covid-19-data-access-guidelines. A small subset of sites who contributed data to this analysis have not agreed to pooled data sharing as above. In the case of requiring access to these data, please contact the corresponding author in the first instance who will look to facilitate access. GR declares receiving a grant from United States National Institute of Health, R01 Grant: Emerging Zoonotic Malaria in Malaysia: Strenghtening Surveillance and Evaluating Population Genetics Structure to Improve Regional Risk Prediction Tool and travel support from the European Society of Clinical Microbiology and Infectious Disease (ESCMID) for observership at European Centre for Disease Prevention and Control (ECDC). All authors declare no competing interests.
