# Supplementary material for: At-admission prediction of mortality and pulmonary embolism in an international cohort of hospitalised patients with COVID-19 using statistical and machine learning methods
Source: Sci Rep. 2024 Jul 16;14:16387. doi: 10.1038/s41598-024-63212-7 (PMC11252333; doi:10.1038/s41598-024-63212-7)
Supplement: Supplementary file 1 — Supplementary Information. [file 41598_2024_63212_MOESM1_ESM.pdf]

I. Libraries and Packages

No minimum sample size was calculated. All significance tests were two-tailed. Analyses were performed using R (version 3.6.2, R Foundation for Statistical Computing, Vienna, Austria) with packages including binom, Epi, ggplot2, lme4, sjstats, tableone, and tidyverse and using Python (version 3.8.0) with packages including imblearn, matplotlib, skopt, xgboost, seaborn, shap, pandas, numpy, and sklearn for machine learning analysis.

The code to reproduce the results as well as the models can be obtained from the following link: https://github.com/Munib5/ISARIC-COVID-19.git. The notebooks contain detailed step-by-step guidance on applying the models and processing the data.

II. Bayesian Optimisation Method

Assume a Gaussian Process (GP) defined by the property that any finite set of N points $\{\mathbf{x}_n \in \mathcal{X}\}_{n=1}^{N}$ induces a multivariate Gaussian distribution on $\mathbb{R}^N$:

$$f : \mathcal{X} \rightarrow \mathbb{R}$$

Assume that the observations are of the form $\{\mathbf{x}_n, y_n\}_{n=1}^{N}$, where $y_n \sim \mathcal{N}\left(f\left(\mathbf{x}_n\right), \nu\right)$ and ν is the variance of noise. This prior and the observations induce a posterior over functions; the acquisition function, which is denoted by $a : \mathcal{X} \rightarrow \mathbb{R}^+$, determines what point in $\mathcal{X}$ should be evaluated next via optimization $\mathbf{x}_{\text{next}} = \operatorname{argmax}_{\mathbf{x}} a(\mathbf{x})$. In other words, an acquisition function is a function of the posterior distribution that describes the utility for all values of hyperparameters. The acquisition functions depend on the previous observations, as well as the GP hyperparameters; the dependence noted as $a\left(\mathbf{x}; \left\{\mathbf{x}_n, y_n\right\}, \theta\right)$. Under the prior, the acquisition functions depend on the model solely through its predictive mean function $\mu\left(\mathbf{x}; \left\{\mathbf{x}_n, y_n\right\}, \theta\right)$ and predictive variance function $\sigma^2\left(\mathbf{x}; \left\{\mathbf{x}_n, y_n\right\}, \theta\right)$ with the best current value as $\mathbf{x}_{\text{best}} = \operatorname{argmin}_{\mathbf{x}_n} f\left(\mathbf{x}_n\right)$ and the cumulative distribution function of the standard normal as $\Phi(\cdot)$. The strategy is to maximize the expected improvement (EI) over the current best and use the highest utility hyperparameter values to compute the next loss.

When maximising the EI one samples from points for which one expects either a higher utility, or points previously unexplored. This approach helps to save both time and computational resources in finding the optimal combination of hyperparameters without trying out all possible combinations. The algorithm can be shortly described as:

1) Given observed values $f(\mathbf{x})$, update the posterior using the GP model
2) Find $\mathbf{x}_{\text{new}}$ that maximises the EI: $\mathbf{x}_{\text{new}} = \arg\max EI(\mathbf{x})$
3) Compute the loss for the point $\mathbf{x}_{\text{new}}$

III. Metrics

The metrics used to evaluate the models include:

1) Area under receiver-operating-characteristic curve (AUROC): an ROC curve is a plot of true positives (TP) as a function of false positives (FP) where each point on the ROC curve represents a sensitivity/specificity pair corresponding to a particular decision threshold. The area under the ROC curve is a summary measure of sensitivity and specificity [**?**].
2) Accuracy, ratio between correctly classified examples and the total number of cases in the dataset. In our case, can be misleading because of class imbalance where simply assigning all examples to the majority class is a way of achieving high accuracy
$$\frac{TP + TN}{TP + TN + FP + FN}$$
3) Weighted F1 score, harmonic mean of precision and recall which penalises extreme values of each weighted by class proportions due to imbalance
$$2 \cdot \frac{PRE \cdot REC}{PRE + REC} = \frac{2TP}{2TP + FP + FN}$$
4) Sensitivity, the probability of a positive prediction for patients with disease (i.e. the conditional probability of correctly identifying diseased patients)
$$\frac{TP}{TP + FN}$$

PRE refers to precision (or positive predicted value) is the ratio of correctly identified positive examples and the total number of predicted positives:

$$\frac{TP}{TP + FP},$$

TP is true positive (correctly classified positive), TN is true negative (correctly classified negative), FP is false positive (falsely classified positive), and FN is false negative (falsely classified negative) cases.

IV. Interpretability Methods

Every classification made by a decision tree can be associated with a corresponding decision path and the F-score is just the number of times a feature is used to split the data across all trees. We use the *shap* library and built on the game-theoretic concept of treating features in the final model as players in a voting game. The method is applied on the entire test set and is based on ideas from game theory [28], [29]. In short, the following equation is used to calculate the Shapley value φ for feature i:

$$\varphi_i(v) = \sum_{S \subset N \setminus \{i\}} \frac{|S|!(n - |S| - 1)!}{n!} (v(S \bigcup \{x_i\}) - v(S)) \tag{1}$$

Where features have their value calculated by taking the difference between the results of the characteristic function v on N (the set of all features) and S (the subset of N without feature i). The Shapley value of a particular feature i is then

calculated by taking the average of the marginal contributions of all possible combinations.

V. Machine Learning Methods

VI. Class Imbalance

PE predictions for XGBoost in Figure 1. On the left we have the XGBoost prediction incapable of learning a clear probability boundary between the heavily imbalanced classes using default parameters and setups.

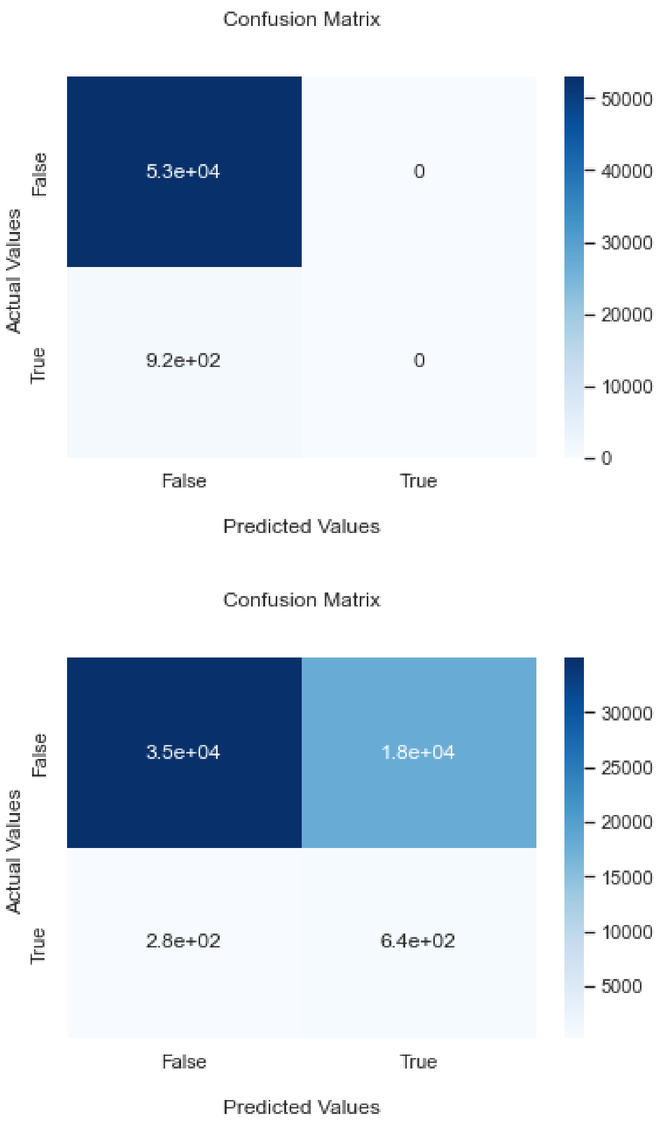

Fig. 1. Confusion Matrices of XGBoost Before And After Imbalance Adjustment

Another example of the need for thresholding can be seen in the prediction probabilities on the training set of the logistic regression model in Figure 2 below. Clearly, the 0.5 default probability threshold will not prove sufficient to capturing the discrimination between the two classes and a lower one would be more suitable. The most optimal threshold, however, would still require increasing the presence of false positives as there is an overlap in the probability densities of the two classes. In our case, luckily, our main care is the level of sensitivity coupled with the AUROC which would capture the majority of true positive cases in rare disease occurrence.

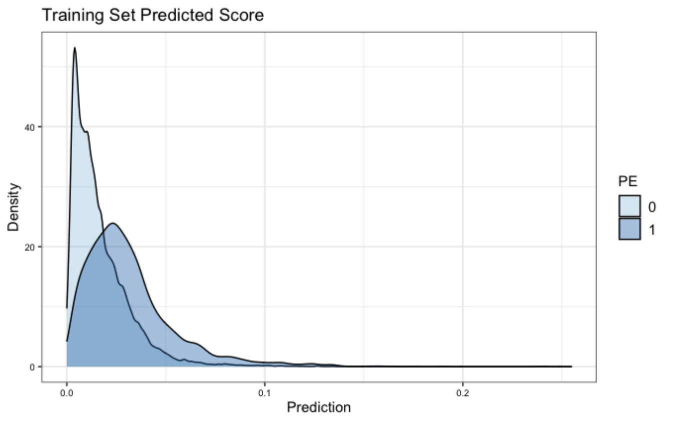

Fig. 2. Probability Prediction Density of Logistic Regression for PE Reveals Trade-off of Sensitivity and Specificity

VII. Correlations

A more detailed representation of the top correlation coefficients is included in Tables II and III.

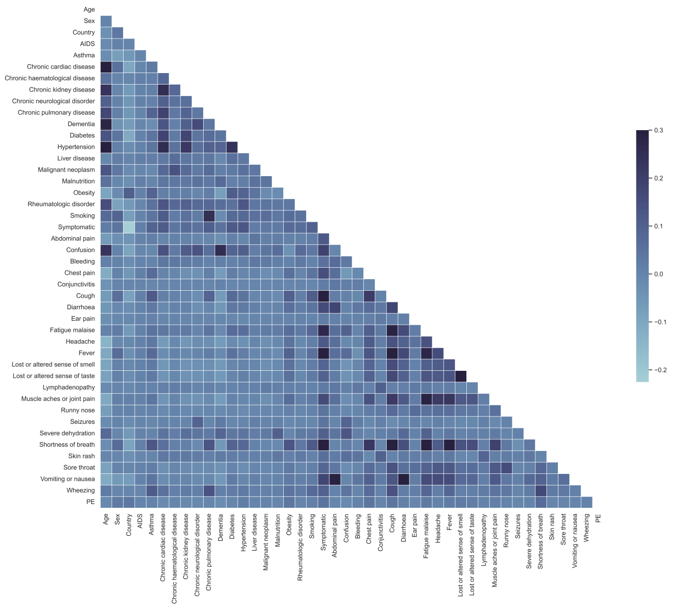

Fig. 3. Correlation of Features with PE (Only Spain and UK Data)

TABLE I
MACHINE LEARNING METHODS DEPLOYED DURING STUDY

**Models**	**Brief Description**
Logistic Regression	Maps a linear relationship taking into account correlations between covariates
Linear Discriminant Analysis	Maps a linear relationship assuming the covariates are independent and normally distributed
Naive Bayes	A probabilistic estimator assuming conditional independence between covariates ignoring correlations
Random Forest	An ensemble of decision trees whose predictions are aggregated for the final prediction
XGBoost	Using extreme gradient-boosting to improve ensembles of random forests for prediction
Ensemble	Using AdaBoosted decision trees, similar to XGBoost but with different boosting mechanism, in an ensemble
Ensemble with XGBoost	Using our XGBoost as the base estimator in the ensemble hierarchy instead of AdaBoost

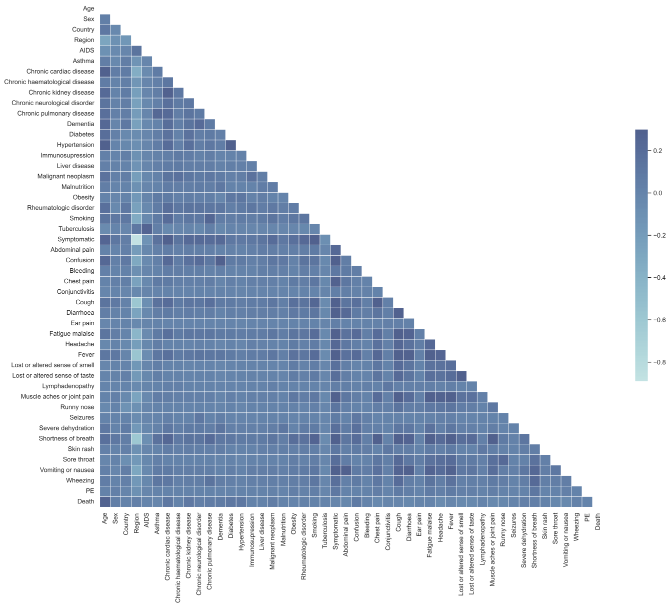

Fig. 4. Correlation of Features with Death

TABLE II
CORRELATIONS OF FEATURES WITH PE (LEFT) AND WITH DEATH (RIGHT)

**Feature**	**PE**	**Death**
D-dimer	0.132	0.012
Shortness of Breath	0.069	0.026
C-Reactive Protein	0.059	0.162
Respiratory Rate	0.048	0.130
Chest Pain	0.043	-0.040
Symptomatic	0.042	-0.011
Neutrophils	0.041	0.099
Cough	0.040	-0.014
Obesity	0.038	0.001
White Blood Cells	0.032	0.090
Heart Rate	0.031	0.014
Fatigue	0.028	-0.002
Sex	0.026	0.044
ALT	0.025	0.009
Fever	0.024	-0.014
Loss of Smell	0.024	-0.040
Loss of Taste	0.022	-0.035
Hypertension	0.018	0.110
Muscle and Joint Pain	0.016	-0.042
Diarrhoea	0.012	-0.024
Smoking	0.010	0.021
Diastolic Blood Pressure	0.010	-0.071
Bilirubin	0.008	0.056
Headache	0.005	-0.054
Wheezing	0.005	0.025
Lymphadenopathy	0.004	0.005
Asthma	0.003	-0.008
Bleeding	0.003	0.006
Malignant Neoplasm	0.002	0.055
Severe Dehydration	0.002	0.033
AIDS	0.001	0.004

VIII. AGE SKEW FOR UK AND SPAIN PATIENTS

It is important to note that the patient populations from Spain and UK are different, especially in their age distribution. When we look at Figures 5 and 6, we see that the patients in Spain are far more likely to be in the 40-80 years band while those in the UK in the <40 and >80 years categories. As age can be an impactful predictor for both PE occurrence and death, it is to be expected that the model results for these two patient populations can differ.

TABLE III
CORRELATIONS OF UK AND SPAIN PATIENT FEATURES WITH PE (LEFT) AND ALL PATIENTS WITH DEATH (RIGHT) (CONTINUED)

**Feature**	**PE**	**Death**
Runny Nose	0.001	-0.022
Haematological Disease	-0.001	0.020
Liver Disease	-0.001	0.012
Rheumatologic Disorder	-0.001	0.023
Tuberculosis	-0.001	0.006
Conjunctivitis	-0.001	-0.007
Sore Throat	-0.001	-0.027
Vomiting	-0.001	-0.039
Platelets	-0.001	0.087
Pulmonary Disease	-0.002	0.064
Systolic Blood Pressure	-0.002	-0.006
Ear Pain	-0.003	-0.006
Lymphocytes	-0.003	-0.018
Skin Rash	-0.004	0.014
Urean	-0.006	0.220
Diabetes	-0.007	0.102
Malnutrition	-0.007	0.020
Abdominal Pain	-0.007	-0.023
Temperature	-0.007	-0.009
Seizures	-0.008	-0.001
Neurological Disorder	-0.010	0.033
Kidney Disease	-0.013	0.092
Age	-0.014	0.278
Confusion	-0.014	0.085
Cardiac Disease	-0.019	0.096
Dementia	-0.024	0.075
Oxygen Saturation	-0.035	-0.109

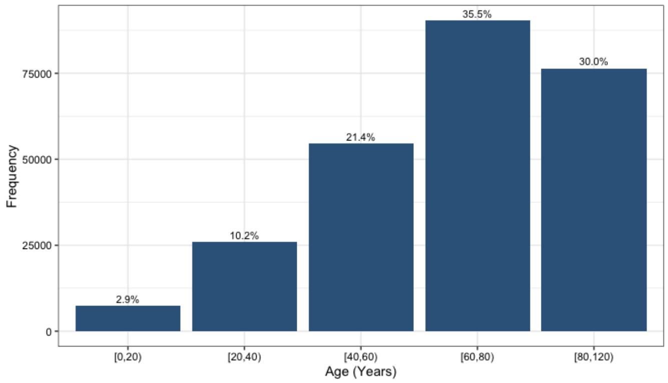

Fig. 5. Age Distribution for UK Patients

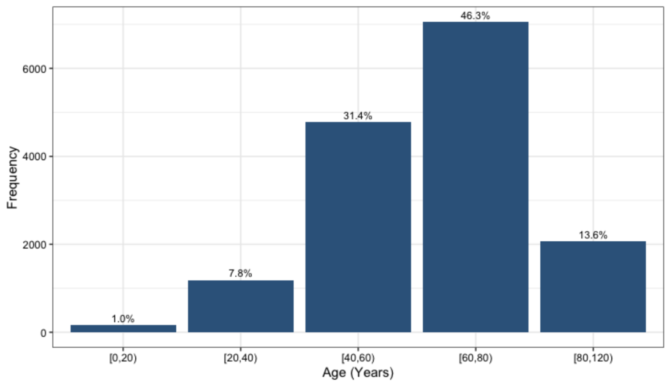

Fig. 6. Age Distribution for Spain Patients

IX. MACHINE LEARNING MODEL SPECIFICATIONS FOR OPTIMISATION

REFERENCES

[1] WHO. Novel coronavirus (2019-ncov): situation report, 11. (2020).

[2] University, J. H. Covid-19 dashboard by the center for systems science and engineering (csse) (2022).

[3] Yang, X. *et al.* Clinical course and outcomes of critically ill patients with sars-cov-2 pneumonia in wuhan, china: a single-centered, retrospective, observational study. *The Lancet Respiratory Medicine* **8**, 475–481 (2020).

[4] Liao, S.-C., Shao, S.-C., Chen, Y.-T., Chen, Y.-C. & Hung, M.-J. Incidence and mortality of pulmonary embolism in covid-19: a systematic review and meta-analysis. *Critical care* **24**, 1–5 (2020).

[5] Knight, S. R. *et al.* Prospective validation of the 4c prognostic models for adults hospitalised with covid-19 using the isaric who clinical characterisation protocol. *Thorax* (2021).

[6] Jones, A. *et al.* External validation of the 4c mortality score among covid-19 patients admitted to hospital in ontario, canada: a retrospective study. *Scientific reports* **11**, 1–7 (2021).

[7] Tabata, S. *et al.* Clinical characteristics of covid-19 in 104 people with sars-cov-2 infection on the diamond princess cruise ship: a retrospective analysis. *The Lancet Infectious Diseases* **20**, 1043–1050 (2020).

[8] Susen, S. *et al.* Prevention of thrombotic risk in hospitalized patients with covid-19 and hemostasis monitoring. *Critical care* **24**, 1–8 (2020).

[9] Whiteley, W. & Wood, A. Risk of arterial and venous thromboses after covid-19. *The Lancet Infectious Diseases* (2022).

[10] Katsoularis, I. *et al.* Risks of deep vein thrombosis, pulmonary embolism, and bleeding after covid-19: nationwide self-controlled cases series and matched cohort study. *bmj* **377** (2022).

[11] Marcos, M. *et al.* Development of a severity of disease score and classification model by machine learning for hospitalized covid-19 patients. *PloS one* **16**, e0240200 (2021).

[12] Venturini, S. *et al.* Classification and analysis of outcome predictors in non-critically ill covid-19 patients. *Internal Medicine Journal* **51**, 506–514 (2021).

[13] Zhou, F. *et al.* Clinical course and risk factors for mortality of adult inpatients with covid-19 in wuhan, china: a retrospective cohort study. *The lancet* **395**, 1054–1062 (2020).

[14] Xie, J. *et al.* Development and external validation of a prognostic multivariable model on admission for hospitalized patients with covid-19. (2020).

[15] Alaa, A., Qian, Z., Rashbass, J., Benger, J. & van der Schaar, M. Retrospective cohort study of admission timing and mortality following covid-19 infection in england. *BMJ open* **10**, e042712 (2020).

[16] van de Sande, D. *et al.* Predicting thromboembolic complications in covid-19 icu patients using machine learning. *Journal of Clinical and Translational Research* **6**, 179 (2020).

[17] Gómez, C. A. *et al.* Mortality and risk factors associated with pulmonary embolism in coronavirus disease 2019 patients: a systematic review and meta-analysis. *Scientific reports* **11**, 1–13 (2021).

[18] Law, N., Chan, J., Kelly, C., Auffermann, W. F. & Dunn, D. P. Incidence of pulmonary embolism in covid-19 infection in the ed: ancestral, delta, omicron variants and vaccines. *Emergency Radiology* 1–5 (2022).

Dataset	Model		Parameters
UK<br>Spain	Logistic<br>Regression	C<br>Regularisation<br>Solver	0.1<br>Lasso (l1)<br>liblinear
UK<br>Spain	Naive<br>Bayes	Smoothing	alpha = 0.0
UK<br>Spain	Linear<br>Discriminant<br>Analysis	Shrinkage<br>Solver	0.17<br>Eigen
UK<br>Spain	Random<br>Forest	Estimators<br>Features<br>Max Depth<br>Minimum Splits<br>Minimum Leaf<br>Bootstrap	150<br>sqrt<br>10<br>5<br>10<br>False
UK<br>Spain	XGBoost	Estimators<br>Learning Rate<br>Max Depth<br>Minimum Splits<br>Maximum Delta<br>Tree Method	150<br>0.1<br>3<br>0.5<br>0<br>hist
UK<br>Spain	AdaBoost Ensemble<br>Ensemble<br>(XGBoost)	Estimators<br>Estimators	150<br>80

TABLE IV
MODEL ARCHITECTURE DETAILS FOR PE

Dataset	Model		Parameters
UK<br>Spain	Logistic<br>Regression	C<br>Regularisation<br>Solver	1.0<br>Lasso (l1)<br>liblinear
UK<br>Spain	Naive<br>Bayes	Smoothing	alpha = 1e-5
UK<br>Spain	Linear<br>Discriminant<br>Analysis	Shrinkage<br>Solver	0.1<br>Eigen
UK<br>Spain	Random<br>Forest	Estimators<br>Features<br>Max Depth<br>Minimum Splits<br>Minimum Leaf<br>Bootstrap	150<br>sqrt<br>None<br>10<br>10<br>True
UK<br>Spain	XGBoost	Estimators<br>Learning Rate<br>Max Depth<br>Minimum Splits<br>Maximum Delta<br>Tree Method	200<br>0.3<br>2<br>0.06<br>0<br>hist

TABLE V
MODEL ARCHITECTURE DETAILS FOR PE (WITH UNDERSAMPLING)

[19] Ikemura, K. *et al.* Using automated machine learning to predict the mortality of patients with covid-19: Prediction model development study. *Journal of medical Internet research* **23**, e23458 (2021).

[20] Alballa, N. & Al-Turaiki, I. Machine learning approaches in covid-19 diagnosis, mortality, and severity risk prediction: A review. *Informatics in Medicine Unlocked* **24**, 100564 (2021).

[21] Akhvlediani, T. *et al.* Isaric clinical characterisation group. *Global outbreak research: Harmony not hegemony. Lancet Infect. Dis* **20**, 770–772 (2020).

[22] Kumari, R. & Srivastava, S. K. Machine learning: A review on binary classification. *International Journal of Computer Applications* **160** (2017).

[23] Chowdhury, M. E. *et al.* An early warning tool for predicting mortality risk of covid-19 patients using machine learning. *Cognitive Computation* 1–16 (2021).

[24] Baqui, P. *et al.* Comparing covid-19 risk factors in brazil using machine learning: the importance of socioeconomic, demographic and structural factors. *Scientific reports* **11**, 1–10 (2021).

[25] Ling, C. X. & Sheng, V. S. Cost-sensitive learning and the class imbalance problem. *Encyclopedia of machine learning* **2011**, 231–235 (2008).

[26] Lemaître, G., Nogueira, F. & Aridas, C. K. Imbalanced-learn: A python toolbox to tackle the curse of imbalanced datasets in machine learning. *The Journal of Machine Learning Research* **18**, 559–563 (2017).

[27] Liu, T.-Y. Easyensemble and feature selection for imbalance data sets. In *2009 international joint conference on bioinformatics, systems biology and intelligent computing*, 517–520 (IEEE, 2009).

[28] Lundberg, S. M. *et al.* From local explanations to global understanding with explainable ai for trees. *Nature machine intelligence* **2**, 56–67 (2020).

[29] Ibrahim, L., Mesinovic, M., Yang, K.-W. & Eid, M. A. Explainable prediction of acute myocardial infarction using machine learning and

Dataset	Model		Parameters
UK<br>Spain	Logistic<br>Regression	C<br>Regularisation<br>Solver	0.01<br>Lasso (l1)<br>liblinear
UK<br>Spain	Naive<br>Bayes	Smoothing	alpha = 0.0
UK<br>Spain	Linear<br>Discriminant<br>Analysis	Shrinkage<br>Solver	0.0<br>lsqr
UK<br>Spain	Random<br>Forest	Estimators<br>Features<br>Max Depth<br>Minimum Splits<br>Minimum Leaf<br>Bootstrap	150<br>auto<br>None<br>10<br>10<br>False
UK<br>Spain	XGBoost	Estimators<br>Learning Rate<br>Max Depth<br>Minimum Splits<br>Maximum Delta<br>Tree Method	350<br>0.1<br>4<br>0.45<br>1<br>hist
UK<br>Spain	AdaBoost Ensemble<br>Ensemble<br>(XGBoost)	Estimators<br>Estimators	20<br>50

TABLE VI
MODEL ARCHITECTURE DETAILS FOR MORTALITY

shapley values. *IEEE Access* **8**, 210410–210417 (2020).
